# Chromosome-level genome assembly of *Aristolochia contorta* provides insights into the biosynthesis of benzylisoquinoline alkaloids and aristolochic acids

**DOI:** 10.1093/hr/uhac005

**Published:** 2022-02-11

**Authors:** Xinyun Cui, Fanqi Meng, Xian Pan, Xiaoxiao Qiu, Sixuan Zhang, Caili Li, Shanfa Lu

**Affiliations:** Medicinal Plant Cultivation Research Center, Institute of Medicinal Plant Development, Chinese Academy of Medical Sciences & Peking Union Medical College, Haidian District, Beijing 100193, China; Medicinal Plant Cultivation Research Center, Institute of Medicinal Plant Development, Chinese Academy of Medical Sciences & Peking Union Medical College, Haidian District, Beijing 100193, China; Medicinal Plant Cultivation Research Center, Institute of Medicinal Plant Development, Chinese Academy of Medical Sciences & Peking Union Medical College, Haidian District, Beijing 100193, China; Medicinal Plant Cultivation Research Center, Institute of Medicinal Plant Development, Chinese Academy of Medical Sciences & Peking Union Medical College, Haidian District, Beijing 100193, China; Medicinal Plant Cultivation Research Center, Institute of Medicinal Plant Development, Chinese Academy of Medical Sciences & Peking Union Medical College, Haidian District, Beijing 100193, China; Medicinal Plant Cultivation Research Center, Institute of Medicinal Plant Development, Chinese Academy of Medical Sciences & Peking Union Medical College, Haidian District, Beijing 100193, China; Medicinal Plant Cultivation Research Center, Institute of Medicinal Plant Development, Chinese Academy of Medical Sciences & Peking Union Medical College, Haidian District, Beijing 100193, China

## Abstract

Aristolochic acids (AAs) and their derivatives are present in multiple Aristolochiaceae species that have been or are being used as medicinal materials. During the past decades, AAs have received increasing attention because of their nephrotoxicity and carcinogenicity. Elimination of AAs from medicinal materials using biotechnological approaches is important for improving medication safety. However, it has not been achieved because of the limited information available on AA biosynthesis. Here, we report a high-quality, reference-grade genome assembly of the AA-containing vine *Aristolochia contorta.* The total size of the assembly is 209.27 Mb, and it is assembled into 7 pseudochromosomes. Synteny analysis, *K*s distribution, and 4DTv suggest an absence of whole-genome duplication (WGD) events in *Aristolochia contorta* after the angiosperm-wide WGD. Based on genomic, transcriptomic, and metabolic data, pathways and candidate genes were proposed for benzylisoquinoline alkaloid (BIA) and AA biosynthesis in *A. contorta*. Five *O*-methyltransferase genes, including *AcOMT1–3*, *AcOMT5*, and *AcOMT7*, were cloned and functionally characterized. The results provide a high-quality reference genome for AA-containing species of Aristolochiaceae. They lay a solid foundation for further elucidation of AA biosynthesis and regulation and for the molecular breeding of Aristolochiaceae medicinal materials.

## Introduction

The safety of aristolochic acids (AAs) and their derivatives has been a matter of concern for researchers worldwide. In the 1990s, the occurrence of “Belgian weight-loss drug (which contained *Aristolochia fangchi*)”, “Gentian purging liver pills (which once contained *A. manshuriensis*)” and other events, as well as the increasing number of cases of renal function damage caused by AA-containing Chinese patent medicines, prompted researchers to find that AAs had obvious nephrotoxicity and carcinogenicity [1, 2]. AAs induce gene mutations to directly damage renal tubular epithelial cells [1], and their nitro and methoxy groups are the key determinants of nephrotoxicity [3]. In 2008, the International Agency for Research on Cancer (IARC) of the World Health Organization (WHO) listed AAs and AA-containing plants as category 1 carcinogens (renal cancer) [4]. AAs exist mainly in Aristolochiaceae genera such as *Aristolochia*, *Asarum* [5], *Saruma* [6], and *Thottea* [7], many of which have been used as medicinal materials. For instance, in the Chinese market, 22 Aristolochiaceae herbs, more than 300 Chinese patent medicines, and 1000 prescriptions that contain Aristolochiaceae herbs have been used [8]. After the discovery of AA nephrotoxicity and carcinogenicity, the use of Aristolochiaceae herbs met with great challenges. AA-containing herbs began to be deleted from the Chinese Pharmacopoeia in 2005. In the most recent Chinese Pharmacopoeia, the vast majority of AA-containing herbs have been deleted, and only one remains (*Asarum sieboldii*) [9].

Over the years, many methods have been used for attenuation of AAs in medicinal herbs, such as processing, concerted application, and traditional breeding. However, these methods have a number of disadvantages, including being unsuitable for quantitative detection, difficult to supervise, or time-consuming and costly. More importantly, these methods cannot eliminate AAs from the herbs. Modern biotechnological tools, such as the CRISPR/Cas9 technique, show promise for the elimination of AAs from sources of medicinal materials. These tools require an understanding of the AA biosynthetic pathway and key enzyme genes. Unfortunately, there is little information available on this pathway, which was analyzed only in a few studies in the 1960s [10–13]. In addition, only one gene (TyrDC) that is probably involved upstream of the pathway has been cloned [14]. In the AA biosynthetic pathway proposed in the 1960s, norlaudanosoline, orientaline, orientalinone, and orientalinol were considered to be intermediates of AA biosynthesis. However, norlaudanosoline was detected only in *Macleaya cordata* [15] and has been proposed to be virtually nonexistent in plants in recent studies. In addition, orientaline, orientalinone, and orientalinol have never been found in Aristolochiaceae plants. Therefore, it is important to revisit the proposed AA biosynthetic pathway. Recently, the biosynthesis of benzylisoquinoline alkaloids (BIAs) has been intensively studied [16]. Because the AAs are BIA derivatives [17], elucidation of BIA biosynthetic pathways provides a foundation for speculation about the AA biosynthetic pathway.

*A. contorta* is a vine from the family Aristolochiaceae in Piperales with significant medicinal and economic value. It is a garden plant that is widely distributed in northern China, Korea, Japan, and Russia. *A. contorta* has been an original plant in traditional Chinese medicinal materials and was included in the official Chinese Pharmacopoeia. All of its organs, including roots, stems, leaves, flowers, and fruits, have been used medicinally for centuries, mainly to treat cough, asthma, and excessive phlegm [18]. Modern pharmacological studies have shown that *A. contorta* has anti-inflammatory, antihypertensive, antibacterial, anti-tumor, antifertility, analgesic, sedative, and other activities [19–21]. Phytochemical analysis showed that it contains abundant bioactive compounds, including alkaloids (such as AAs [22], aristolactams [23], magnoflorine and allantoin [24]), monoterpenes and sesquiterpenes (such as caryophyllene [25] and aristolactone [26]), β-sitosterol [24], pinitol, daucosterol [27], and other components.

With the aim of elucidating the biosynthetic pathways of AAs, we sequenced and assembled the whole genome of *A. contorta* using Illumina paired-end reads, Nanopore sequencing, and Hi-C technology. Genomic studies of *A. contorta* provide insight into the evolutionary position of the magnoliids and the absence of whole-genome duplication (WGD) events in *Aristolochia*. By combining genome sequencing with analysis of the transcriptome and metabolome, we identified candidate genes involved in the biosynthetic pathways of sanguinarine, berberine, and magnoflorine in *A. contorta*. In addition, possible biosynthetic pathways of aristolochic acids were proposed for further validation. The *A. contorta* genome assembly provides insights into the phylogenomics of magnoliids and AA biosynthesis. It also lays a solid foundation for the molecular breeding of Aristolochiaceae medicinal materials.

## Results and discussion

### Genome sequencing, assembly and annotation

The sample used for whole genome sequencing was from an accession of *A. contorta* growing in the Medicinal Botanical Garden of the Institute of Medicinal Plant Development in Beijing, China. The size of the *A. contorta* genome was estimated to be 290.64 Mb, with repeat and heterozygosity percentages of 47.14% and 0.83%, respectively, as estimated by *k*-mer analysis based on next-generation sequencing data (Fig. S1). Illumina HiSeq technology, Oxford Nanopore Technologies (ONT), and Hi-C technology were integrated to sequence and assemble a chromosome-level genome assembly. A total of 26.86 Gb of reads were obtained from Illumina HiSeq, and the sequencing depth was 83.04×. A total of 38.59 Gb of clean data with an N50 of 42.26 kb were obtained after filtering 46.75 Gb of raw ONT reads generated using the PromethION platform. The longest ONT read was 798.932 kb, and the average depth of sequencing was approximately 183×. The primary assembly of ONT reads was performed with Canu, wtdbg, and SMARTdenovo, and the assembly was then corrected using the Illumina data. These steps resulted in a primary assembled genome of 210.53 Mb, which was composed of 173 contigs, with a contig N50 of 2.63 Mb (Table 1).

**Table 1:** Statistics and characteristics of the *A. contorta* genome

**Assembly feature**	**Statistics**
Estimated genome size (by k-mer analysis) (Mb)	290.64
*Nanopore sequencing*	
Contig number	173
Contig N50 (Mb)	2.63
Genome size (Mb)	210.53
*Hi-C assembly*	
Scaffold number	84
Scaffold N50 (Mb)	30.38
Longest scaffold (Mb)	38.74
Contig number	224
Contig N50 (Mb)	2.32
Assembled genome size (Mb)	209.27
Assembly % of genome	99.4

During construction of the Hi-C library, a total of 149 355 995 raw paired-end reads were generated, from which 12 799 624 valid interaction reads were obtained using HiC-Pro. Then, 99.4% of the genome sequences were anchored into 7 (2n = 14) pseudochromosomes using LACHESIS software. The intense intra-chromosomal interaction signals indicated a high-quality Hi-C assembly (Fig. S2). The final genome assembly of *A. contorta* was 209.27 Mb (99.4% coverage of the genome). It was composed of 214 contigs with a contig N50 of 2.32 Mb and a scaffold N50 of 30.38 Mb. Among the contigs, 147 (198.56 Mb) were anchored with an exact order and direction (Tables 1, S1 and S2).

**Figure 1:**
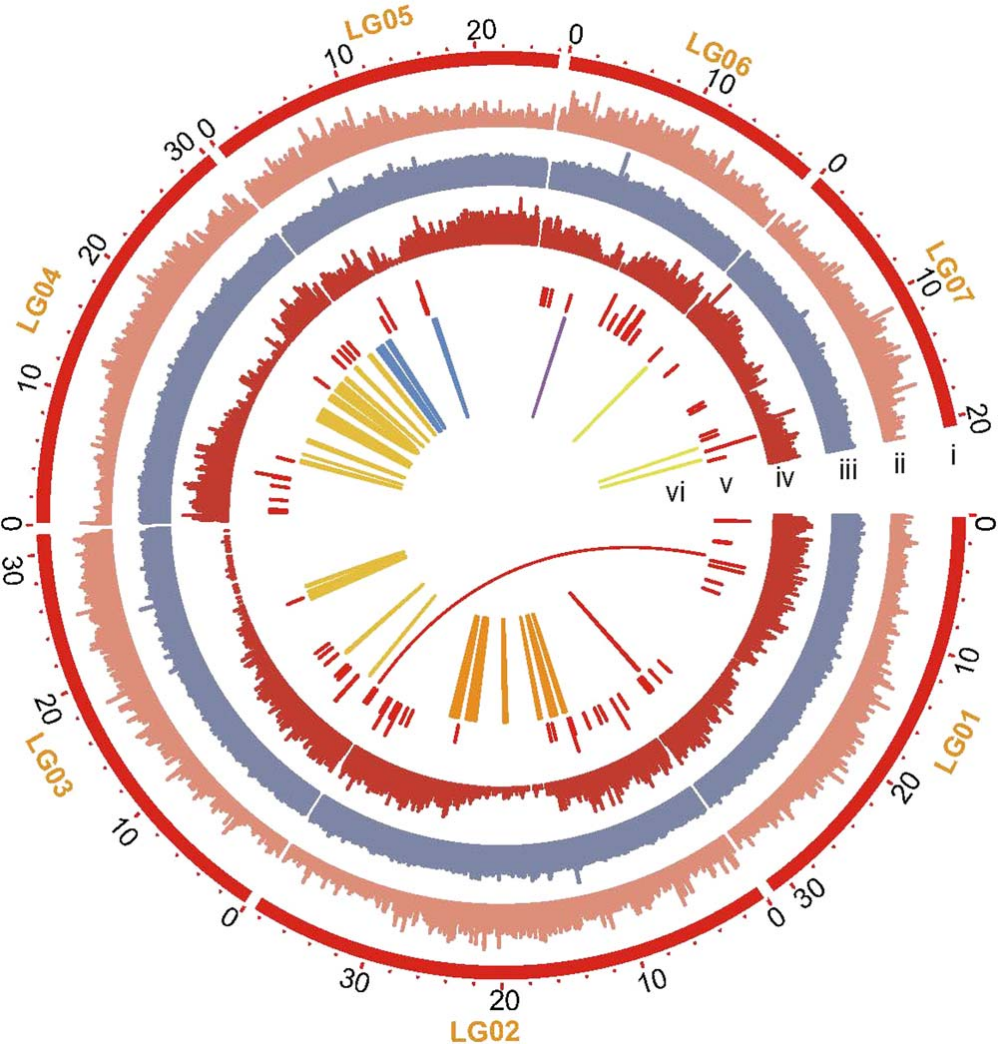
Overview of *A. contorta* genomic features and intragenomic synteny information. (i) Assembled pseudochromosomes; (ii) Gene density; (iii) GC content; (iv) Repetitive sequence density; (v) Non-coding RNA density; and (vi) Genome syntenic blocks. Syntenic blocks in different chromosomes are distinguished by colors.

BUSCO and CEGMA analyses showed that 90.28% and 97.38% of the conserved core genes of eukaryotes, respectively, were identified in the *A. contorta* genome (Table S3). Moreover, mapping of the high-quality 350-bp Illumina reads to the assembled genome showed that the mapping rate was 96.85%. These results suggest the high completeness and contiguity of the final genome assembly (Fig. 1).

Based on the construction of a species-specific repetitive sequence database, 80.56 Mb of repetitive sequences were identified, constituting 38.26% of the whole genome. Among these sequences, approximately 33.05 Mb were long terminal repeats (LTRs), accounting for 41.04% of all repetitive sequences. This result suggests that LTRs are the dominant repetitive sequences in the *A. contorta* genome. Further analysis of LTRs showed that Copia (21.99 Mb) and Gypsy (10.87 Mb) were the most common subtypes, accounting for 27.31% and 13.49%, respectively (Table S4). Ab initio prediction, homology-based prediction, and unigenes from RNA-seq were used to predict protein-coding genes (Fig. S3). After integration and modification, 18 311 protein-coding genes were obtained (Table S5). They were composed of 27.95 Mb of exons with an average gene length of 1.53 kb (Table S6). Noncoding RNA prediction identified 46 microRNAs, 475 tRNAs, and 404 rRNAs. GeneWise and genBlastA were used to find 770 pseudogenes. Functional analysis of the assembled genome using the Kyoto Encyclopedia of Genes and Genomes (KEGG), eukaryotic orthologous groups (KOG), gene ontology (GO), TrEMBL, and nonredundant protein sequence (Nr) databases annotated 97.02% of the predicted genes (Table S7).

### Phylogenomic analysis

*A. contorta* belongs to the magnoliids. Currently, there are controversial views on the taxonomic status of magnoliids. To address this problem, the genome assembly of *A. contorta* was compared with 17 other sequenced genomes from two ANA-grade angiosperm species, *Amborella trichopoda* and *Nymphaea colorata*; eight eudicot species, *Papaver somniferum*, *M. cordata*, *Nelumbo nucifera*, *Vitis vinifera*, *Glycine max*, *Arabidopsis thaliana*, *Coffea canephora* and *Helianthus annuus*; three magnoliid species, *Piper nigrum*, *Cinnamomum kanehirae*, and *Liriodendron chinense*; three monocots, *Lemna minor* from the order Alismatales, *Oryza sativa* from the order Poales, and *Dendrobium officinale* from the order Asparagales; and the gymnosperm species *Ginkgo biloba*, which served as an outgroup.

**Figure 2:**
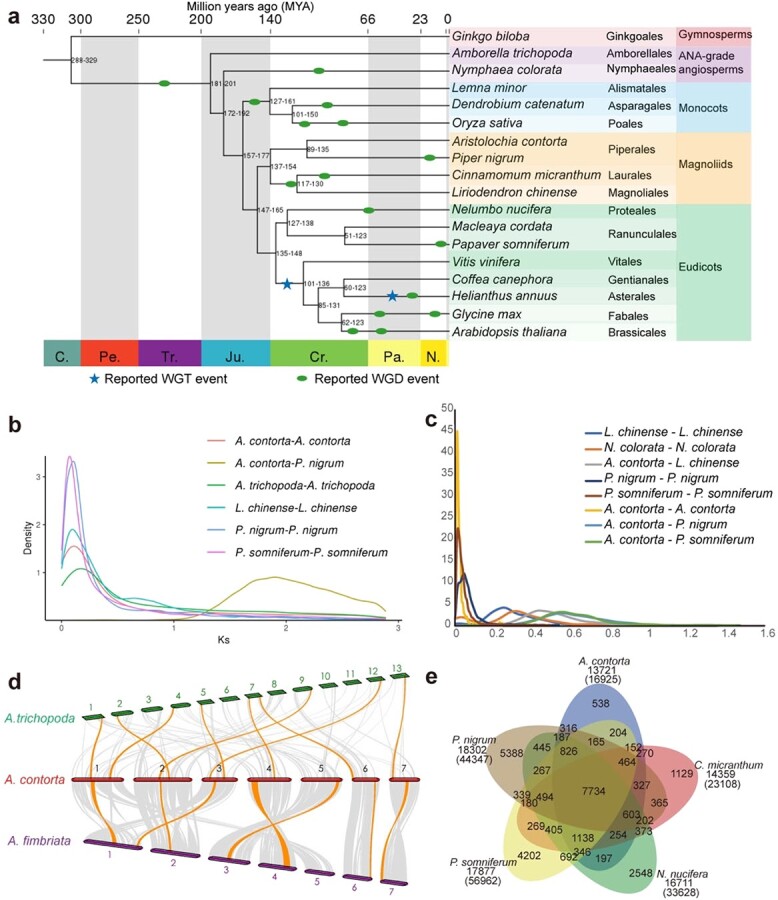
Evolutionary and comparative genomic analyses. **a** Phylogenetic tree of 18 plant species with speculated divergence times at each node. The timings of reported WGT and WGD events are superimposed on the tree. **b** Distribution of *K*s distances. **c** Distribution of 4DTv distances. **d** Synteny analysis of *A. contorta*, *A. trichopoda*, and *A. fimbriata.*  **e** Venn diagram representing the clusters of gene families in *A. contorta* and four related plants.

A phylogenomic analysis of 641 single-copy orthologous gene sequences placed monocots as a sister clade to the magnoliids + eudicots clade with 100% bootstrap support (Figs. 2a and S4). This result is in agreement with results from a phylotranscriptomic analysis of 92 streptophytes and land plants [28], an angiosperm phylogeny of 26 species [29], a phylogenomic analysis of the stout camphor tree [30], and a phylogenomic analysis of one thousand plant transcriptomes [31]. However, it contrasts with the classification of the sister relationship between magnoliids and a clade including monocots and eudicots in APG IV (Angiosperm Phylogeny Group, http://www.mobot.org/MOBOT/research/APweb/) and with results from phylogenomic analyses of angiosperms, such as *Magnolia biondii* [32], *L. chinense* [33], and *Phoebe bournei* [34]. In addition, Qin et al*.* performed an analysis of genome structural rearrangements in *Aristolochia fimbriata* and various other plant species [35]. Their results showed that eudicots were sister to the magnoliid + monocot clade [35].

To further investigate the taxonomic status of magnoliids, we constructed a phylogenetic tree using the chloroplast genomes of *A. contorta* and 35 other plant species. The results showed that magnoliids were sister to the monocot + eudicot clade (Fig. S5). This result is inconsistent with the classification in the phylogenomic analysis of 18 representative plant genomes, above. Phylogenetic discordance for the position of magnoliids is probably due to incomplete lineage sorting [36] that resulted from the rapid radiation of early-diverging lineages within angiosperms (such as the magnoliids, monocots, and eudicots) that occurred within a very short period of time of <5 million years [32]. Ancient hybridization and parallel evolution that probably occurred during plant evolution may also have interfered with the results of phylogenetic analysis [35]. In addition, deviation may also be caused by aspects of lineage sampling, such as incomplete taxon sampling and the selection of Gramineae that could increase the likelihood of long branch attraction [37]. Moreover, different analytical methods (maximum likelihood, Bayesian [34], concatenation, coalescent, supermatrix, and ASTRAL methods [36]) and different sequence types (amino acid, nucleotide, and partitioned codons) could cause phylogenetic discrepancies. In fact, phylogenomic analyses of *A. fimbriata* and different taxon samples using the ASTRAL and supermatrix methods showed obscure topologies of monocots, eudicots, and magnoliids [35]. Therefore, pure phylogenomic analysis is not the best way to resolve the evolutionary position of magnoliids. With the increasing number of sequenced genomes and the availability of more rigorous analytical methods and balanced taxon sampling, more powerful phylogenomic evidence will be obtained for the placement of magnoliids. We estimated the time of divergence between magnoliids and eudicots to be 147–165 million years ago (MYA) using the MCMC tree with fossil calibration. This estimate is supported by 95% highest posterior density. In addition, *A. contorta* and *Piper nigrum* (black pepper, also in the order Piperales), which diverged 89–135 MYA, had the closest evolutionary relationship. Piperales was classified as a sister group to a clade comprising Magnoliales and Laurales among the magnoliids (Fig. 2a). Piperales first diverged from Magnoliales–Laurales approximately 137–154 MYA (Fig. 2a).

It is worth noting that the phylogenetic relationships within the order Piperales are still uncertain. APG IV accepted three families (Saururaceae, Piperaceae, and Aristolochiaceae) in Piperales. However, recent studies have shown that it is more reasonable to consider Piperales as an order with six families: Saururaceae, Piperaceae, Aristolochiaceae, Asaraceae, Hydnoraceae, and Lactoridaceae [38]. The reference-grade genome of *Aristolochia contorta* can lay the foundation for further resolving the phylogenetic positions of magnoliids in angiosperms and Aristolochiaceae in Piperales.

### Analysis of whole-genome duplication

Many plants have experienced shared ancestral whole-genome duplication (WGD) events or lineage-specific WGD events, which make important contributions to species diversification [39]. For instance, an ancient WGD occurred just before the divergence of Laurales and Magnoliales, and a more recent WGD was shared by all lineages of Lauraceae [34, 36]. To assess the WGD events in *A. contorta*, we performed a comparative analysis of a range of species, including *P. somniferum* (in which a WGD event occurred ~7.8 MYA) [40], *L. chinense* (in which a WGD event occurred ~116 MYA) [33], *P. nigrum* (in which a recent WGD event occurred ~17 MYA) [41] and *V. vinifera* (a representative of the closest modern species to the ancestral eudicot karyotype [AEK]) [42, 43]. Synonymous substitution rate (*K*s) distributions and fourfold synonymous (degenerative) third-codon transversion (4DTv) (Fig. 2b, Fig. 2c) confirmed the WGT-γ in *V. vinifera* [42] and recent species-specific WGD events in *P. somniferum, L. chinense, Nymphaea colorata, P. nigrum* and *N. nucifera* [33, 40, 41, 44, 45]*.* Intragenomic synteny analysis of *A. contorta* using MCScanX showed 50 syntenic regions throughout the whole genome; these included 1202 genes that represented 6.56% of all genes. The dot and line plot of the synteny analysis showed that 99% of the replications were intra-chromosomal duplications (Fig. 1 and Fig. S6). Very sparse syntenic blocks existed in the *A. contorta* genome, consistent with a syntenic analysis of the *A. fimbriata* genome [35]. Based on the *K*s distributions and 4DTv rates, a major peak (*K*s value of 0.2; 4DTv value of 0.01) was observed in the *A. contorta K*s and 4DTv curve (Fig. 2b, Fig. 2c). This kind of peak could be caused by tandem repeats [46] but not indicate the occurrence of a recent WGD event in *A. contorta*. In addition, *Amborella trichopoda* and *A. fimbriata* are known not to have undergone any lineage-specific WGDs after their divergence from the last common ancestor of extant flowering plants [35, 47]. Comparative genomic synteny analysis of *A. contorta* with *A. trichopoda* and *A. fimbriata* revealed 1:1 and 1:1 syntenic depth ratios in the comparisons of *A. contorta* with *A. trichopoda* and *A. fimbriata*, respectively (Fig. 2d). These results strongly suggest the absence of a lineage-specific whole-genome duplication event in *A. contorta*. In addition, transcriptome phylogenetic analysis of 1000 plants showed that *Aristolochia elegans* had also not undergone any WGD after the angiosperm-wide WGD [31]. Because no WGD was identified in any of the three *Aristolochia* species (*A. contorta*, *A. fimbriata* and *A. elegans*) analyzed to date, it is very likely that no *Aristolochia* lineage-specific WGD event has occurred in the evolutionary history of the genus*.*

Because of a lack of WGD events and subsequent subgenome rearrangements, the genomes of *Aristolochia* species are relatively concise, and this can help us to identify WGDs in magnoliids and other early angiosperms through genome comparison. By comparison of intergenomic syntenic blocks of *A. fimbriata* and *P. nigrum*, Qin et al. [35] identified another two ancient WGDs in *P. nigrum* that were not reported in the genome article [41]. Here we performed comparative genomic synteny analysis for *A. contorta* and *P. nigrum*. The dot plot clearly showed a syntenic depth ratio of 1:8 in the comparison of *A. contorta* and *P. nigrum* (Fig. S7). The results are consistent with those obtained by Qin et al. [35]. *A. contorta* has a smaller genome than *A. trichopoda* and *A. fimbriata*. It can serve as another useful reference for phylogenomic studies of angiosperms. Moreover, the highly conserved synteny between *Amborella* and *Aristolochia* provides insights into the reconstruction of the ancestral angiosperm genome [35]. We expect that the release of more *Aristolochia* genomes will enable the construction of a highly credible ancestral angiosperm genome. This will promote the analysis of quantitative changes and evolutionary schemes of *A. contorta* genes retained from the ancestral genome after the angiosperm-wide WGD.

### Comparative genomic analysis

In order to identify potential genes encoding enzymes in the biosynthetic pathways of AAs or other important bioactive compounds, the *A. contorta* genome assembly was compared with 11 other sequenced genomes from two basal angiosperms, *Amborella trichopoda* and *Nymphaea colorata*; four eudicot species, *P. somniferum*, *M. cordata*, *N. nucifera*, and *V. vinifera*; three magnoliid species, *P. nigrum*, *C. kanehirae*, and *L. chinense*; and two monocots, *O. sativa* and *Zea mays*. A total of 47 786 gene families were identified, including 3495 shared gene families and 201 gene families that were unique to the *A. contorta* genome (Fig. S8). Gene copy number analysis showed that *A. contorta* contained 63.7% single-copy genes and 16.9% two-copy genes (Fig. S9). Patterns of gene-family sharing were studied among *A. contorta*, *P. somniferum*, *L. chinense*, *N. nucifera*, and *Cinnamomum micranthum* (Fig. 2e). A total of 11 contracted and 25 expanded gene families were predicted (Fig. S10). According to GO enrichment annotations and KEGG pathway classification, the expanded families were enriched in sesquiterpene biosynthesis. This coincides with the fact that *A. contorta* plants have pungent odors and contain large amounts of volatile oil, the largest proportion of which is sesquiterpenoids. Among these compounds, caryophyllene is the main component, accounting for one third of the total volatile oil [25]. It has anti-asthmatic effects and is one of the active ingredients for the treatment of aged chronic bronchitis, as well as a local anesthetic. Borneol has diaphoretic, excitement, and antispasmodic effects, whereas pinene has antitussive and expectorant properties and is antifungal [48]. The efficacy of *A. contorta* in traditional Chinese medicine may be closely related to the pharmacological activities of these volatile components that it contains.

In order to study the unique functions of genes in *A. contorta*, positive selection analysis was performed among *L. chinense, C. micranthum, A. contorta, M. cordata,* and *N. nucifera*. It resulted in the identification of 94 *A. contorta* genes that were subjected to significant positive selection. GO enrichment analysis of these genes indicated that most of them were related to cellular nitrogen compound metabolic process, metal ion binding, methyltransferase activity, nucleolus, and rRNA processing. Classification of positively selected genes by KEGG pathways identified those for basal transcription factors, RNA degradation, ether lipid metabolism, and so forth. In particular, the term cellular nitrogen compound metabolic process may represent the metabolism of *A. contorta*–specific compounds, AAs, *in vivo*. Methyltransferase activity was one of the most enriched molecular function GO terms. It may be related to new functions of *O*-methyltransferases (OMTs), *N*-methyltransferases (NMTs), and other methyltransferases in *A. contorta*.

In addition, specific gene families in *A. contorta* were analyzed using GO and KEGG enrichment analysis (Fig. S11). There were several terms related to secondary metabolism in GO enrichment analysis of unique genes, including cellular aromatic compound metabolic process, sesquiterpene biosynthetic process, methylation, oxidoreductase activity, sesquiterpene synthase activity, S-adenosylmethionine-dependent methyltransferase activity, and others. Various *OMT*, *NMT*, and norcoclaurine synthase (*NCS*) genes were identified as unique genes related to the GO terms. KEGG analysis showed that the specific gene families were enriched in limonene and pinene degradation, glucosinolate biosynthesis, and the biosynthesis of stilbenoids, diarylheptanoids, and gingerols. In addition, a variety of cytochrome P450 gene families, such as CYP706, CYP86B, CYP82C, and CYP71 involved in secondary metabolism, were annotated as specific gene families of *A. contorta*. These results indicate the significance of *A. contorta* OMTs and CYPs in the biosynthesis and metabolism of unique compounds such as AAs and BIAs.

### Identification and characterization of the *AcOMT* gene family

**Figure 3:**
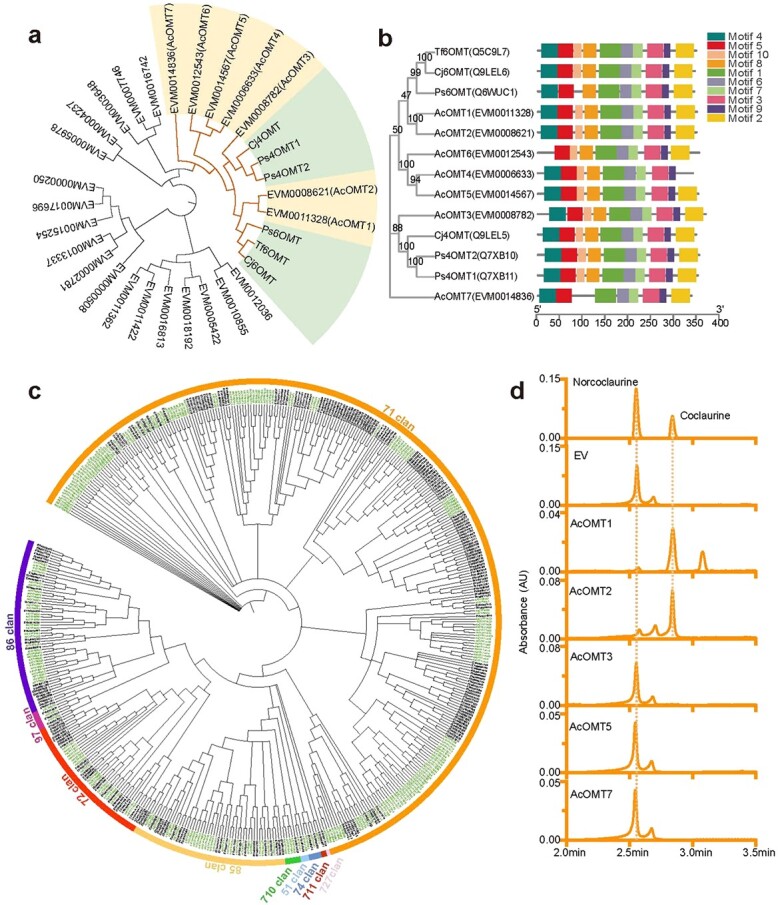
Phylogenetic analyses of AcOMTs and AcCYPs and functional identification of norcoclaurine 6-*O*-methyltransferase. **a** Phylogenetic tree of AcOMTs and the reference proteins Ps6OMT, Ps4′OMT1, Ps4′OMT2, Cj6OMT, Cj4′OMT, and Tf6OMT constructed with MEGA 7.0 using default parameters and 1000 bootstraps. Ps, *Papaver somniferum*; Cj, *Coptis japonica*; Tf, *Thalictrum flavum*. **b** Distribution of conserved motifs on seven selected AcOMT proteins and six reference proteins. **c** Neighbor-joining phylogenetic tree of cytochrome P450 genes. *A. contorta*: black; *A. thaliana*: green. **d** UPLC analysis of purified enzyme incubated with norcoclaurine as a substrate *in vitro*. Top, norcoclaurine and coclaurine standard; EV, *E. coli* carrying the empty vector; AcOMT1–AcOMT7, *E. coli* expressing the corresponding proteins.

*O*-methylation in the biosynthesis of multiple secondary metabolites is primarily performed by *S*-adenosyl-_L_-methionine (SAM)-dependent *O*-methyltransferases (OMTs) encoded by the *OMT* gene family. Plant OMTs deliver the methyl group of SAM to the hydroxyl group of alkaloids, flavonoids, phytoalexins, and lignin, resulting in the production of their methyl ether derivatives [49]. The addition of a methyl group to an alkaloid structure has a significant effect on its chemical properties and biological activity [50]. To study the functions of the *OMT* gene family in the biosynthesis of BIAs and their derivatives in *A. contorta*, genome-wide identification of *AcOMTs* was performed. The results indicated that there were 25 *AcOMT* genes in *A. contorta*. Neighbor-joining phylogenetic analysis of 25 AcOMT proteins and six BIA-related OMTs from *P. somniferum* (Ps6OMT, Ps4′OMT1, and Ps4′OMT2), *Coptis japonica* (Cj6OMT and Cj4′OMT) and *Thalictrum flavum* (Tf6OMT) [51–55] using MEGA 7.0 with default parameters and 1000 bootstraps identified seven full-length AcOMT homologs: AcOMT1 (EVM0011328), AcOMT2 (EVM0008621), AcOMT3 (EVM0008782), AcOMT4 (EVM0006633), AcOMT5 (EVM0014567), AcOMT6 (EVM0012543), and AcOMT7 (EVM0014836) (Fig. 3a). MEME analysis of the seven identified AcOMTs and six BIA-related OMTs from other plant species showed ten conserved motifs (Fig. 3b). The results suggested that these OMTs could have similar functions in BIA biosynthesis, although their actual catalytic functions remain to be investigated.

### Identification and characterization of the *AcCYP* gene family

The cytochrome P450 superfamily (CYP) is a large superfamily of monooxygenases. It plays an important role in many secondary metabolic pathways, such as pathways for the biosynthesis of terpenoids, flavonoids, fatty acids, lignin, alkaloids, and other metabolites [56–59]. CYPs mainly catalyze the hydroxylation of substrate molecules. However, some of them are involved in the reactions of C–C phenol-coupling, C–O phenol-coupling, and methylenedioxy bridge formation, which are unusual but crucial for the biosynthesis of metabolites, particularly in the biosynthetic pathway of BIAs [60, 61] and very probably in the biosynthetic pathway of AAs.

A total of 241 full-length *CYP* genes were identified from the genome of *A. contorta*. This is comparable to the number in *A. thaliana*, which contains 247 *CYP* genes. The 241 nonredundant *A. contorta* CYPs were identified to family or subfamily (Table S8). Conserved motif analysis showed that the PERF motif, K-helix region, I-helix region, and heme-binding motif were present in the P450 protein sequences of *A. contorta* (Fig. S12). To investigate the evolutionary relationships among P450 protein sequences from *A. contorta*, a phylogenetic tree of 241 AcCYPs and 247 AtCYPs was constructed (Fig. 3c). The results showed that AcCYP proteins could be divided into 10 clans. Among them, the 71 clan contains the A-type CYPs, whereas the others contain non-A-type CYPs. The 71 clan is also the largest clan, with 156 AcCYPs (64.73%). All of the families and subfamilies previously found to take part in the biosynthesis of BIAs, such as the CYP80B and CYP80G subfamilies and the CYP719 family, belong to the 71 clan. Analysis of the distribution of *AcCYP* genes around the genome showed that 235 *AcCYP* genes were spread across the 7 pseudochromosomes (Fig. S13), and pseudochromosome LG02 had the largest number of *AcCYP* genes (68). In addition, members of the CYP80 gene family were located on pseudochromosomes LG04, LG05, LG06, and LG07, and a member of the CYP719 gene family was located on pseudochromosome LG05.

### Metabolome profiling and analysis of AA contents in *A. contorta*

**Figure 4:**
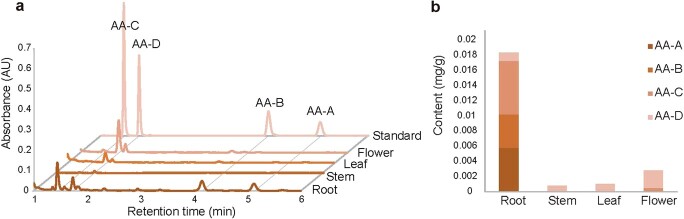
AA contents in different tissues of *A. contorta*. **a** Representative UPLC chromatograms of roots, stems, leaves, and flowers of *A. contorta*. **b** The contents (mg/g) of AAs, including aristolochic acid A (AA-A), aristolochic acid B (AA-B), aristolochic acid C (AA-C), and aristolochic acid D (AA-D), in roots, stems, leaves, and flowers of *A. contorta*.

To date, phytochemical studies on the alkaloids of *A. contorta* have focused mainly on AAs and aristolactams (ALs). The AAs identified include aristolochic acids I–VII, E, F, G, and their derivatives, such as aristolochic acid Ia, aristolochic acid IVb, and 7-hydroxyaristolochic acid I. The ALs identified include aristolactams I, II, III, IV and their derivatives, such as aristolactam IIIa and aristololactam Ia-N-β-D-glucopyranoside. In addition, caryophyllene, pinene, borneol, aristolactone, aristolone, β-sitosterol, allantoin, magnoflorine, pinitol, and daucosterol have also been identified in *A. contorta*. However, there are few reports on BIAs and intermediate compounds of the AA biosynthetic pathway. To comprehensively identify the metabolites produced in *A. contorta*, the metabolome in four tissues of *A. contorta* was analyzed. This resulted in the identification of 1147 compounds, including protopine, norsanguinarine, morphine, noscapine, anonaine, and other BIAs (Table S9). In addition, the contents of AAs in different parts of *A. contorta* plants were determined using UPLC. The results showed that AAs accumulated primarily in roots, followed by flowers. The accumulation of AAs, particularly aristolochic acid A and aristolochic acid B, was much lower in stems and leaves (Fig. 4).

### Identification of candidate genes involved in BIA biosynthetic pathways in *A. contorta*

BIAs are a group of plant-specific metabolites with multiple structures that have a long history of investigation [62]. The pharmacological activities and biosynthetic pathways of various BIA compounds, such as antimicrobial sanguinarine and berberine, anti-diabetic magnoflorine, and analgesic morphine, have been comprehensively studied in multiple plant species, including *P. somniferum*, *M. cordata*, and *Coptis japonica* [15, 63–66]. BIA compounds have a collective upstream biosynthetic pathway, which includes the condensation of tyrosine derivatives dopamine and 4-hydroxyphenylacetaldehyde and four other enzymatic steps to yield the critical branch node intermediate (*S*)-reticuline [52]. The biosynthesis of BIAs, including morphinan (morphine and codeine), benzophenanthridine (sanguinarine), and protoberberine (berberine) [62], proceeds from the intermediate (*S*)-reticuline. To date, little is known about the biosynthetic pathways of BIAs in *A. contorta*.

**Figure 5:**
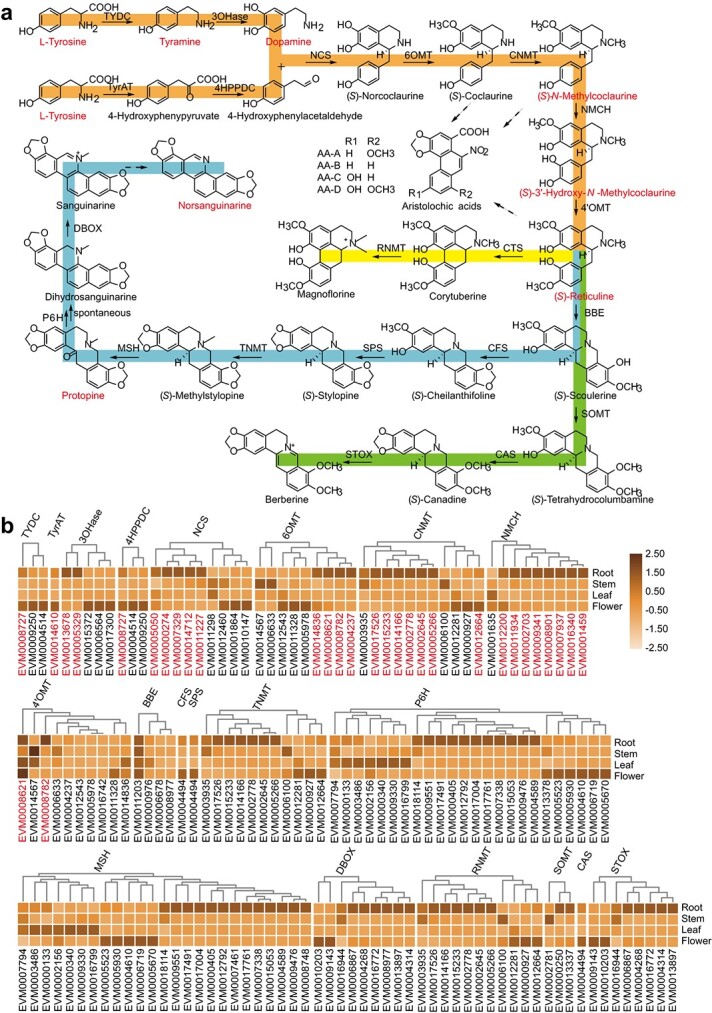
Prediction of BIA biosynthetic pathways and candidate genes in *A. contorta.*  **a** Prediction of the biosynthetic pathways of (*S*)-reticuline (orange), magnoflorine (yellow), norsanguinarine (blue), and berberine (green) in *A. contorta*. Compounds detected in the metabolic data are shown in red. **b** Expression profile of 91 candidate genes potentially involved in BIA biosynthesis. Gene expression values (FPKM) were scaled by column *Z*-score. Twenty-nine nonredundant genes that are probably involved in AA biosynthesis are shown in red. Heat map values represent relative gene expression in each tissue after column normalization. TYDC, tyrosine decarboxylase; 3OHase, tyrosine 3-hydroxylase; TyrAT, tyrosine aminotransferase; 4HPPDC, 4-hydroxyphenylacetaldehyde; NCS, norcoclaurine synthase; 6OMT, norcoclaurine 6-*O*-methyltransferase; CNMT, coclaurine *N*-methyltransferase; NMCH, (*S*)-*N*-methylcoclaurine 3′-hydroxylase; 4′OMT, 3′-hydroxy-*N*-methylcoclaurine 4′-*O*-methyltransferase; BBE, berberine bridge enzyme; CTS, corytuberine synthase; RNMT, reticuline *N*-methyltransferase; SOMT, scoulerine 9-*O*-methyltransferase; CAS, canadine synthase; STOX, tetrahydroprotoberberine oxidase; CFS, cheilanthifoline synthase; SPS, stylopine synthase; TNMT, tetrahydroprotoberberine *N*-methyltransferase; MSH, *N*-methylstylopine 14-hydroxylase; P6H, protopine 6-hydroxylase; DBOX, dihydrobenzophenanthridine oxidase.

Based on previous studies of magnoflorine, sanguinarine, and berberine [61, 64, 67–69], we proposed the biosynthetic pathways of BIAs in *A. contorta*. As shown in Fig. 5a, the common biosynthetic pathway of BIAs (orange) begins with the condensation of L-tyrosine derivatives, dopamine and 4-hydroxyphenylacetaldehyde, to yield (*S*)-norcoclaurine through the catalysis of NCS [70, 71]. The important intermediate (*S*)-reticuline that acts as the central branch point of several BIAs is then synthesized from (*S*)-norcoclaurine through the catalysis of four enzymes: 6OMT, CNMT, NMCH, and 4′OMT [67, 72–76]. Finally, magnoflorine (yellow), sanguinarine (blue), and berberine (green) are produced from (*S*)-reticuline through the catalysis of multiple enzymes [15, 64, 77]. Genome-wide analysis predicted 91 nonredundant genes that were probably involved in the biosynthesis of BIAs in *A. contorta* (Table S10). Transcriptome analysis of *A. contorta* roots, stems, leaves, and flowers showed that the identified candidate genes were differentially expressed (Fig. 5b). The expression of fifteen candidate genes was validated by qRT-PCR (Fig. S14). These data provide the basis for functional analysis of genes involved in BIA biosynthetic pathways.

### Enzyme assay of norcoclaurine 6-*O*-methyltransferase

(*S*)-norcoclaurine-6-*O*-methyltransferase (6OMT) is a critical rate-limiting enzyme that participates in the biosynthesis of reticuline, the versatile intermediate shared by various end-products of BIA biosynthesis [52]. To identify the *6OMT* genes in *A. contorta*, seven of the nine *6OMT* candidate genes (Fig. 5b), which were highly expressed in at least one analyzed tissue, were cloned and introduced into *Escherichia coli* cells to obtain recombinant protein. *In vitro* assays showed that *E. coli* cells produced a main band at the theoretical molecular mass of His-tagged AcOMTs (~47 kDa) after IPTG induction (Figs. S15 and S16). With the exception of *AcOMT3-*transformed *E. coli*, this band was clearly observed in the supernatant of the cell lysate, indicating the production of abundant soluble AcOMT protein. SDS-PAGE analysis of purified soluble AcOMT1, AcOMT2, AcOMT5, and AcOMT7 proteins and renatured inclusion bodies of AcOMT3 are shown in Figs. S15, S17, and S18. Norcoclaurine was tested as a potential substrate. The enzyme activity of AcOMTs was analyzed in Tris–HCl buffer and detected using UPLC. As shown in Fig. 3d, a coclaurine peak was detected in the reaction of AcOMT1 and AcOMT2 enzymes. UPLC analyses demonstrated that AcOMT1 and AcOMT2 could convert norcoclaurine into coclaurine (Fig. 3d). An unknown peak with a retention time of 3.08 min was observed in the assay of AcOMT1 enzyme. Based on the functional characteristics of *O*-methyltransferase, this peak may have resulted from the conversion of the hydroxyl group at the 7 or 4′ position to methoxy. Thus, *AcOMT1* and *AcOMT2* probably encode 6OMT in *A. contorta*, whereas the functions of the other *AcOMT* genes remain to be elucidated.

### Possible biosynthetic pathways of AAs in *A. contorta*

Based on genomic and transcriptomic data from *A. contorta* and the results obtained from previous BIA biosynthetic pathway studies, we deduced and predicted possible pathways and candidate genes for AA biosynthesis in *A. contorta.* AAs are biogenetically derived from BIA precursors, and magnoflorine is always identified together with AAs in various *Aristolochia* species [11]. In addition, norlaudanosoline has been considered to be an intermediate of AA biosynthetic pathways for several decades [10–14]. However, the distribution of norlaudanosoline in plants is narrow: it has only been found in *M. cordata* [15]. Thus, the important intermediate compound in the AA biosynthetic pathway could be (*S*)-norcoclaurine but not norlaudanosoline. Based on information from BIA biosynthetic pathway analysis, we propose that the upstream portion of AA biosynthetic pathways is essentially the same as that of the BIA biosynthetic pathways shown in Fig. 5a (orange). It involves enzymes from the norcoclaurine synthase (NCS) family, *O*-methyltransferase (OMT) family, *N*-methyltransferase (NMT) family’ and cytochrome P450 (CYP) superfamily and leads to the formation of the intermediate (*S*)-reticuline (Fig. 5a). Then, a novel C–C bond is formed between carbon 8 and carbon 2′ on the benzyl of (*S*)-reticuline through a C–C phenol-coupling reaction, resulting in the production of the basic skeleton of aporphine alkaloids. Subsequently, the methoxy and hydroxyl groups on carbon 6 and carbon 7 are condensed to generate the methylenedioxy bridge, and the oxidative ring-opening of the nitrogen heterocycle occurs to form the final backbone of AAs. Substitutions of different types of functional groups at different positions form different types of AAs, such as aristolochic acids A, B, C, and D. Because laurelliptine, nandigerine, anonaine, and dehydroaporheine were identified in the metabolome (Table S9), we speculate that (*S*)-coclaurine and (*S*)-*N*-methylcoclaurine [78] may also serve as intermediates for the C–C phenol-coupling reaction, methylenedioxy bridge generation, and other subsequent reactions (Fig. 5a). Based on gene expression patterns and AA contents in different tissues, 29 nonredundant genes probably involved in AA biosynthesis were identified for further validation (Fig. 5b, shown in red).

## Conclusions

A chromosome-level reference genome assembly of *A. contorta* was constructed using the Nanopore and Hi-C technologies. The assembled genome is 209.27 Mb in size, with contig and scaffold N50 sizes of 2.63 Mb and 30.38 Mb, respectively. The sequence was anchored into 7 pseudochromosomes. Phylogenetic analysis showed conflicting results for the taxonomic status of magnoliids. Synteny analysis, *K*s distribution, and 4DTv rates suggested the absence of lineage-specific WGD events in *A. contorta*. Comparative genomic analysis identified 11 contracted and 25 expanded gene families and indicated the importance of *A. contorta* OMTs and CYPs in the biosynthesis of AAs and BIAs. Genome-wide analysis identified 25 *AcOMT* and 241 *AcCYP* genes in *A. contorta*. In addition, 91 genes encoding enzymes likely to be involved in BIA biosynthetic pathways were predicted. Among them, *AcOMT1* and *AcOMT2* were experimentally confirmed to encode 6OMT in *A. contorta*. AA biosynthetic pathways were also proposed for further validation. *Aristolochia* species include a variety of medicinal plants and have been used in Asia and other regions for a long time. AAs and their derivatives are one of the most important groups of compounds produced in *Aristolochia* species. To date, significant attention has been paid to *Aristolochia*-derived herbal drugs because of aristolochic acid nephropathy (AAN) [79, 80]. This chromosome-level genome assembly of *A. contorta* provides a high-quality reference genome for AA-containing plant species and provides insights into the phylogenomics of magnoliids and the molecular breeding of Aristolochiaceae.

## Materials and methods

### Sample collection and genome sequencing

DNA was extracted from young leaves of *A. contorta* collected from the Medicinal Botanical Garden of the Institute of Medicinal Plant Development. Two 350-bp Illumina DNA libraries were constructed and sequenced using an Illumina sequencer with a read length of 150 bp. The nanopore library was constructed with high-molecular-weight DNA according to the ONT protocol using the Ligation Sequencing kit (SQK-LSK109) and sequenced using the PromethION Flow Cell Priming Kit (EXP-FLP001.PRO.6) (Oxford Nanopore Technologies, UK) according to the manufacturer’s instructions. The ONT reads were self-corrected using Canu [81], and the corrected reads were assembled into contigs using SMARTdenovo (https://github.com/ruanjue/smartdenovo). The assembled contigs were polished using Racon [82] and Pilon [83]. The quality of the genome assembly was assessed by BUSCO (https://busco.ezlab.org/) and CEGMA [84] analyses.

Hi-C fragment libraries were constructed and sequenced using the Illumina platform with insert sizes of 300–700 bp. The raw reads were trimmed to obtain clean Hi-C reads, which were then mapped to the genome assembly using BWA [85] (version 0.7.10-r789). The uniquely alignable paired reads (mapping quality>20) were retained for scaffold assembly using LACHESIS [86] with the parameters “CLUSTER_MIN_RE_SITES, 60; CLUSTER_MAX_LINK_DENSITY, 2; ORDER_MIN_N_RES_IN_TRUN, 58; ORDER_MIN_N_RES_IN_SHREDS, 55” after filtering invalid reads with HiC-Pro [87] v2.8.1.

### Gene prediction and functional annotation

To predict repeated sequences in the *A. contorta* genome, a *de novo* repeat library was constructed using LTR_FINDER [88] and RepeatScout [89]. A repeat database was built using PASTEClassifier [90] and Repbase [91]. Repetitive sequences were predicted using RepeatMasker [92]. The Rfam [93] database was used to predict rRNAs and miRNAs, and tRNAscan-SE [94] v1.3.1 software was used to predict tRNAs. GeneWise [95] was used to predict pseudogene homolog sequences.

Protein-coding genes were predicted using a combination of *de novo* gene prediction, homolog prediction, and unigene sequence prediction. GENSCAN [96], Augustus [97] v2.4, GlimmerHMM [98] v3.0.4, geneid [99] v1.4, and SNAP v1.3.1 were used for *de novo* gene prediction. GeMoMa [100] v1.3.1 was used for homolog prediction. HISAT [101] v2.0.4, StringTie [102] v1.2.3, TransDecoder v2.0, GeneMarkS-T [103] v5.1, and PASA [104] v2.0.2 were used to predict unigene sequences from transcriptome data. EVM 1.1.1 and PASA v2.0.2 were used to combine and revise all of the results. The predicted protein-coding genes were annotated by BLAST [105] (v2.2.31) analysis against the NR [106], KOG [107], GO, KEGG [108], and TrEMBL [109] databases.

### Comparative analysis of genomes

For phylogenetic analysis, an evolutionary tree was constructed with single-copy orthologous genes from at least 13 of the 18 analyzed species using IQ-TREE v1.6.11 [110]. Gene sequences were aligned using MAFFT v7.205 [111] with the parameters -localpair -maxiterate 1000. Conversion of protein alignments into codon alignments was performed using PAL2NAL v14 [112]. Poorly aligned regions were removed using Gblocks v0.91b [113] (−b5 = h). All of the remaining gene family sequences were connected to obtain a supergene. ModelFinder [114] in IQ-TREE was used to perform model detection, and the best model was determined to be JTT + F + I + G4. An evolutionary tree was constructed using the maximum likelihood (ML) method with 1000 bootstraps. *G. biloba* served as the outgroup to obtain a rooted evolutionary tree. Divergence times were calculated using PAML v4.9i [115]. Published data from *A. trichopoda - V. vinifera* (173–199 MYA), *P. somniferum - N. nucifera* (127.9–139.4 MYA), and *L. chinense – C. kanehirae* (117–130 MYA) were used to calibrate the divergence times.

The complete chloroplast genomes of 36 species were downloaded from the NCBI nucleotide database (Table S11). Whole sequences were aligned using MAFFT v7.487 [111], and gaps were removed using Gblocks v0.91b [116] (−b5 = n). The best-fit model was determined to be GTR + F + R5 using ModelFinder [114] in IQ-TREE v2.1.4_beta [113]. The ML tree was constructed with 1000 bootstraps. *G. biloba* served as the outgroup for the phylogenomic tree.

For comparative genomic analysis, a phylogenetic tree was constructed for 12 species based on 1199 single-copy genes in at least 10 species using the same method described above. Protein sequences of *A. contorta* and other 11 species, *A. trichopoda*, *N. colorata*, *O. sativa*, *Z. mays*, *P. nigrum*, *L. chinense*, *C. kanehirae*, *N. nucifera*, *P. somniferum*, *M. cordata*, and *V. vinifera*, were classified by the software Orthofinder v2.4 [116]. The obtained gene families were annotated with the PANTHER V15 database [117]. GO and KEGG enrichment analyses of gene families unique to *A. contorta* were performed using clusterProfiler v3.14.0 [118]. The numbers of expanded and contracted gene families on each phylogenetic tree branch were calculated based on differentiation times and gene family clustering using CAFE v4.2 [119]. Positive selection analysis was performed using the CodeML program in PAML. Single-copy genes from *A. contorta*, *L. chinense*, *C. micranthum*, *M. cordata*, and *N. nucifera* were analyzed using MAFFT, PAL2NAL, model A and the null model of PAML, and the branch-site model of CodeML to perform likelihood ratio tests (*P* < 0.01). The Bayes method was used to identify genes that were subjected to significant positive selection. Single-copy genes in *A. contorta* with a *K*a/*K*s ratio > 1 and posterior probability >0.95 were considered to be under significant positive selection.

### Synteny and WGD event analysis

Gene sequences from two species were compared, and similar gene pairs were determined using Diamond v0.9.29.130 with *E*-value<1 × 10^−5^ and C score > 0.5. The C score value was filtered by JCVI software [120]. MCScanX [121] and the gff3 file for the genome assembly were used to determine whether similar gene pairs were adjacent on the chromosome. The maximum gap between the anchor genes was set to 30 to obtain all syntenic gene blocks. Whole genome duplication (WGD) events were identified by a *K*s distribution analysis using wgd v1.1.1 [122], and fourfold synonymous third-codon transversion (4DTv) rate was determined using a Perl script (https://github.com/JinfengChen/Scripts).

### Gene identification and functional characterization of AcOMTs

The hidden Markov model (HMM) corresponding to the *O*-methyltransferase (OMT) domain, including methyltransf_2 (PF00891) and methyltransf_3 (PF01596), was acquired from the Pfam database (http://pfam.sanger.ac.uk/). *OMT* genes were identified using HMMER 3.0 in the *A. contorta* genome. Default parameters were used, and the threshold was set to 1e^−5^. Six OMTs, Ps6OMT, Tf6OMT, Cj6OMT, Ps4′OMT1, Ps4′OMT2, and Cj4′OMT, were used as the initial query sequences to search the *A. contorta* protein database using the BLASTP program. The results of the HMM and BLASTP programs were merged. After deleting all redundant sequences, the hypothetical OMT protein sequences were submitted to CDD (https://www.ncbi.nlm.nih.gov/Structure/bwrpsb/bwrpsb.cgi) and Pfam to confirm the presence of the conserved OMT domain. MEME (https://meme-suite.org/meme/tools/meme) was used to analyze conserved motif structures.

Full-length cDNAs of the *AcOMTs* were acquired by PCR amplification with the primers listed in Table S12. The cDNAs were cloned into the pET30a vector and introduced into BL21(DE3). Transformed *E. coli* cells were cultured in LB liquid medium supplemented with 50 μg mL^−1^ kanamycin and induced with 0.5 mM IPTG at 16°C for 5 hours at a low speed. The cells were collected by centrifugation and vortexed in binding buffer. After that, the suspension was sonicated at 200 W. Cell debris was eliminated by centrifugation at 13 000 rpm for 8 min. The soluble protein was purified using Ni-NTA Resin (Transgen Biotech, Beijing) according to the manufacturer’s instructions. To isolate AcOMT3 protein, the purified inclusion body was denatured with 6 M guanidine hydrochloride, renatured by dialysis, and then dissolved in Tris–HCl. SDS-PAGE and Coomassie blue staining were used to confirm the purity of the His-tagged proteins. The purified proteins were analyzed with the BCA Protein Assay kit (Takara Biomedical Technology, Beijing) to determine the protein concentration.

The enzyme assay for OMT activity was performed in a 50-μl reaction system that comprised 25 mM Tris–HCl (pH 8.0), 25 mM sodium ascorbate (Sigma), 0.1 mM *S*-adenosyl-_L_-methionine (Sigma), 100 mM substrate, and 10 μg purified enzyme. The reactions were incubated at 37°C for 1 h and terminated by adding 100 μL of methanol. Controls were performed with total protein from *E. coli* transformed with the empty pET-30a vector [52, 56]. After centrifugation, 10-μL supernatants were injected into the ACQUITY UPLC system (Waters, Milford, MA, USA). The samples were separated on an ACQUITY UPLC BEH C18 column (1.7 μm, 100 × 2.1 mm) at 35°C. The mobile phases were methanol (A) and water containing 0.1% formic acid (B). The mobile phases changed according to the following gradient with a flow rate of 0.3 mL min^−1^: 0–0.5 min, holding at 5% A; 0.5–4 min, increasing from 5% A to 95% A; 4–6 min, holding at 95% A; 6–6.1 min, falling to 5% A; 6.1–8 min, holding at 5% A. Coclaurine was detected using a photodiode array detector at an absorbance of 280 nm.

### Cytochrome P450 gene superfamily identification and analysis

The HMM file for cytochrome P450 (PF00067) was downloaded from the Pfam database (http://pfam.xfam.org/) and used to search for *CYP* genes in the *A. contorta* genome with hmmsearch (HMMER 3.2.1). The *A. thaliana CYP* genes were downloaded from the Arabidopsis Information Resource (https://www.arabidopsis.org/) and used to search for *CYP* genes in the *A. contorta* genome using BLASTP with *E*-value≤1e^−40^. After removing redundant sequences from the HMM and BLASTP search results, the presence of the conserved CYP domain in the putative CYP protein sequences was confirmed using CDD (https://www.ncbi.nlm.nih.gov/Structure/bwrpsb/bwrpsb.cgi) and SMART (http://smart.embl-heidelberg.de/#). A manual analysis was performed to identify full-length sequences. Based on the criteria of the CYP nomenclature system [123], in which amino acid sequence similarity of P450 families is >40% and that of subfamilies is >55% (https://www.arabidopsis.org/browse/genefamily/p450.jsp), the families and subfamilies of the *A. contorta CYP* genes were named. MEME was used to analyze conserved motif structures. Multiple sequence alignment of *A. contorta* and *A. thaliana* CYPs was performed with MUSCLE included in MEGA7. The neighbor-joining (NJ) tree was constructed using MEGA7 with 1000 bootstrap replications and a site coverage cutoff of 50%. The NJ tree was visualized using iTOL (https://itol.embl.de/).

### RNA sequencing and gene expression analysis

Total RNA was isolated from four tissues of *A. contorta*: roots, stems, leaves, and flowers. Sequencing libraries were generated by mRNA isolation, fragmentation, first-strand cDNA synthesis, second-strand cDNA synthesis, and purification using the NEBNext Ultra RNA Library Prep kit according to the manufacturer’s instructions. The library preparations were sequenced on an Illumina platform (NEB, USA). Clean reads were obtained after removing adaptor sequences and low-quality sequence reads from the raw data and then mapped to the draft genome of *A. contorta* using HISAT2. Gene expression levels were estimated by FPKM values (fragments per kilobase of transcript per million fragments mapped) using StringTie [124].

### Metabolite analyses

Roots, stems, leaves, and fruits of *A. contorta* were used for metabolite analysis on an LC/MS system. The UHPLC separation was performed using a 1290 Infinity series UHPLC System (Agilent Technologies). MS/MS spectra were obtained using the TripleTOF 6600 mass spectrometry system (AB Sciex) on an information-dependent basis (IDA). Analyst TF 1.7 (AB Sciex), ProteoWizard, and XCMS [125] software were used for data analysis.

### Validation of gene expression levels by quantitative real-time PCR (qRT-PCR)

To validate the digital expression data, the transcript levels of 15 selected genes in different tissues were measured by qRT-PCR. Total RNA was extracted using the EASYspin Plus Complex Plant RNA kit (Aidlab, Beijing, China). First-strand cDNA was synthesized using the PrimeScript RT reagent kit with gDNA Eraser (Takara). The qRT-PCR primers designed using Oligo 7 are shown in Table S13. *AcActin* was used as the reference gene. qRT-PCR reactions were performed using TB Green Premix Ex Taq II (Takara) on a Bio-Rad CFX96 Real-Time system. Data for gene expression levels in different tissues were analyzed using the 2^−ΔΔCt^ method.

### Identification of candidates in the BIAs pathway of *A. contorta*

Local BLASTP was used to identify candidate genes involved in the BIA pathways. Reference protein sequences from *P. somniferum*, *C. japonica*, *M. cordata*, and *Eschscholzia californica* were obtained from the NCBI and UniProt databases and aligned against the *A. contorta* genome using BLASTP with a maximum *E*-value of 1e^−10^. The results were filtered by identity >40% and coverage >50% to allow for distant species relationships. Based on the gene family characteristics of NCS sequences, the identity value was set to 30% [71]. A heatmap was generated using TBtools with column scaling [126].

### Aristolochic acid content analysis

*A. contorta* tissues were ground in liquid nitrogen. Samples of powder (0.1 g) were dissolved in 2 mL of 70% methanol. The mixture was allowed to stand at room temperature for 15 min and then sonicated for 30 min. After centrifugation, the supernatant was collected. The sediment was dissolved in 70% methanol and then sonicated for 30 min. This supernatant was collected after centrifugation and combined with the previous supernatant. All supernatants were filtered through a 0.22-μm membrane and injected into the UPLC. Aristolochic acids A, B, C, and D were quantitatively detected via an ACQUITY UPLC system (Waters, Milford, MA, USA) with an ACQUITY UPLC BEH C18 column (1.7 μm, 100 × 2.1 mm) at 30°C. The mobile phases were acetonitrile and water containing 0.2% acetic acid. The elution was carried out at a flow rate of 0.4 mL min^−1^ with 40% acetonitrile. AAs were detected using a photodiode array detector at 254 nm absorbance.

## Supplementary Material

Web_Material_uhac005

## Data Availability

The assembled *A. contorta* genome sequence and raw transcriptome sequencing data are available from the NCBI database under project ID PRJNA738351 and PRJNA742069. The annotation information has been uploaded to the CoGe website ( https://genomevolution.org/coge/SearchResults.pl?s=Aristolochia%20contorta&p=genome).
