# Supplementary material for: Chromosome-level genome assembly of Aristolochia contorta provides insights into the biosynthesis of benzylisoquinoline alkaloids and aristolochic acids
Source: Hortic Res. 2022 Feb 11;9:uhac005. doi: 10.1093/hr/uhac005 (PMC8973263; doi:10.1093/hr/uhac005)
Supplement: Web_Material_uhac005 [file web_material_uhac005.zip › FigS1-11-1014.docx]

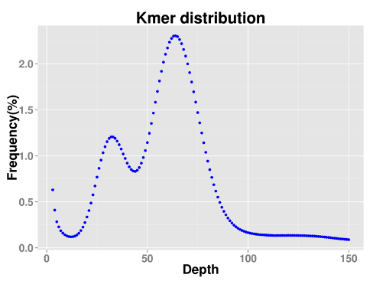


**﻿ Fig. S1 Evaluation of *A. contorta* genome size by k-mer analysis (k=19).**

**
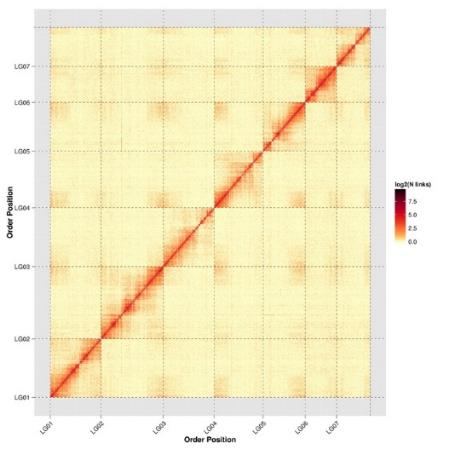
**

**Fig. S2 Hi–C heat map of chromosome interactions.** LG01**–**07 represent the 7 chromosomes. Both x-axis and y-axis represent the order of each bin on the corresponding chromosome group. The color of the bar on the right represents the strength of interaction.


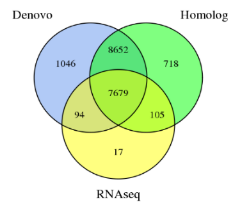


**Fig. S3 Genes derived from the distribution map of the three prediction methods.**


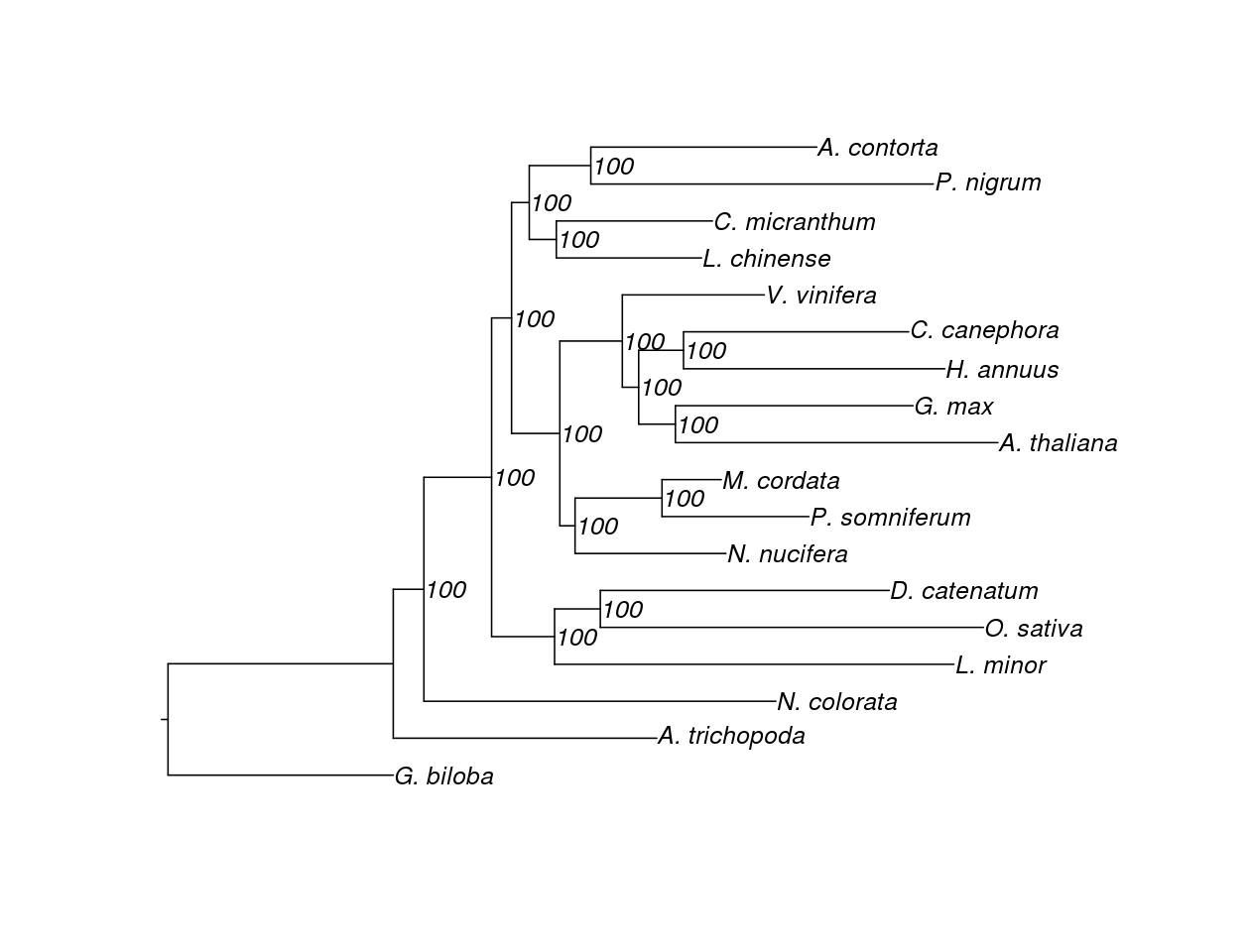


**Fig. S4 Phylogenetic tree with bootstrap support values of each node among 18 plant species.**


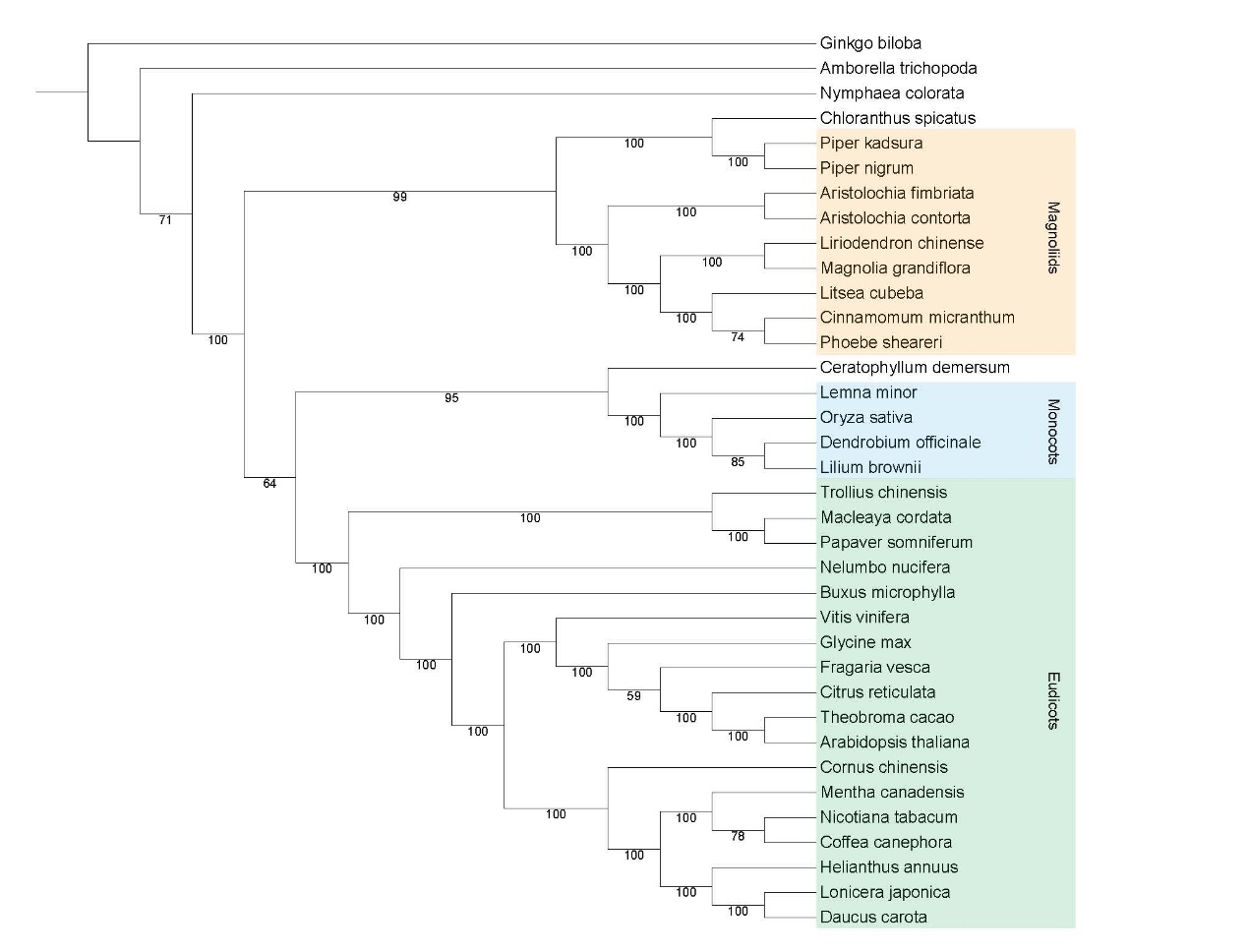


**Fig. S5 Phylogenetic tree of chloroplast genomes among 36 species using ML method.**

**
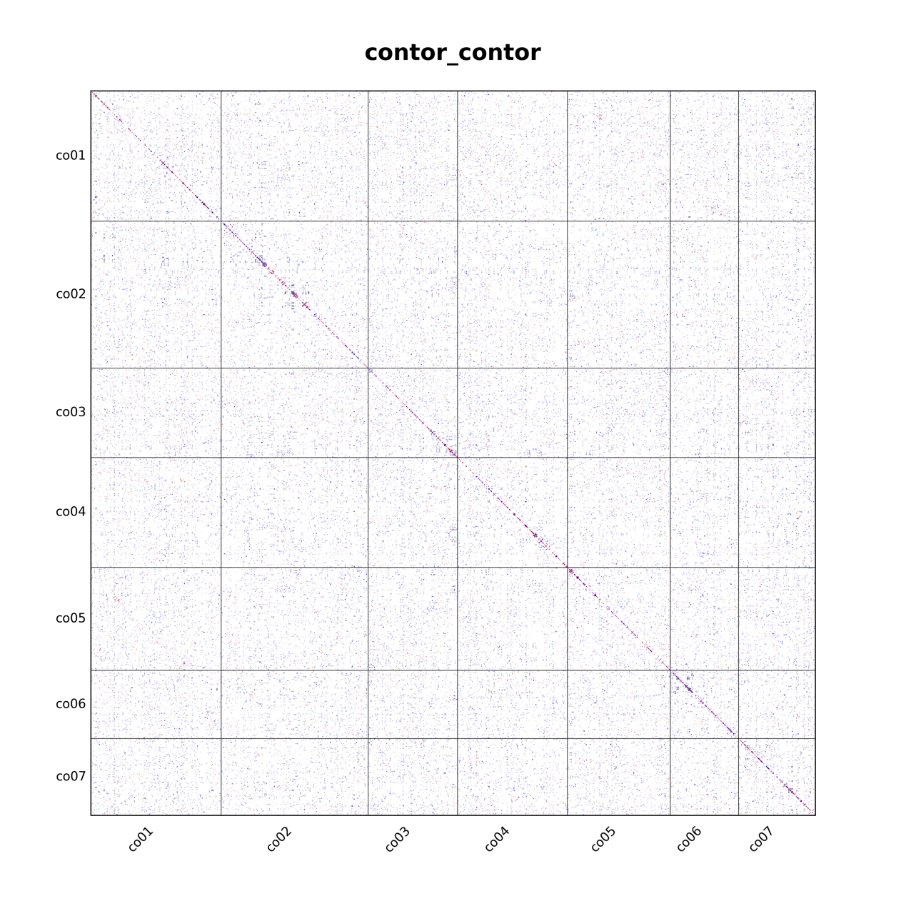
**

**﻿Fig. S6 ﻿The synteny of *A. contorta* paralogous gene pairs.** The paralogs displayed by dot plot matrix in *A. contorta*.


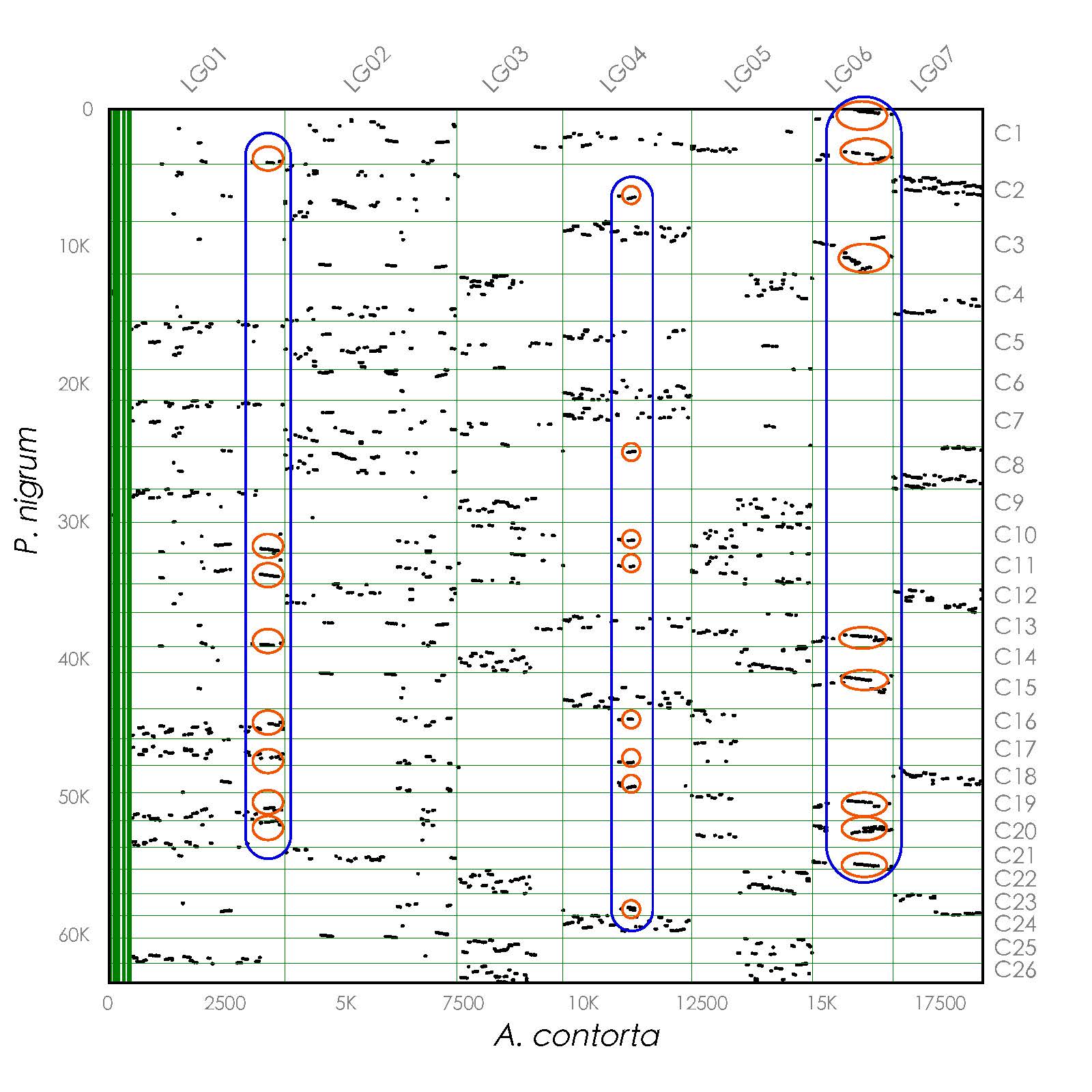


**Fig. S7 ﻿Syntenic dotplot between the *A. contorta* and *P. nigrum* genomes**


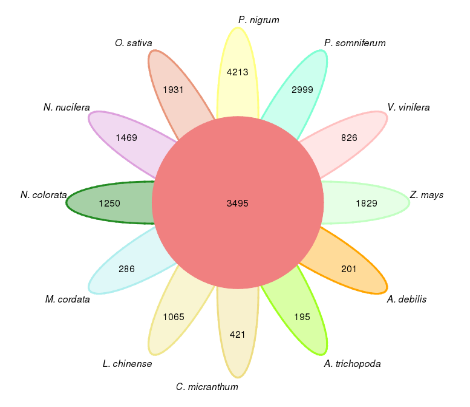


**Fig. S8 Gene family cluster petal diagram.**

**
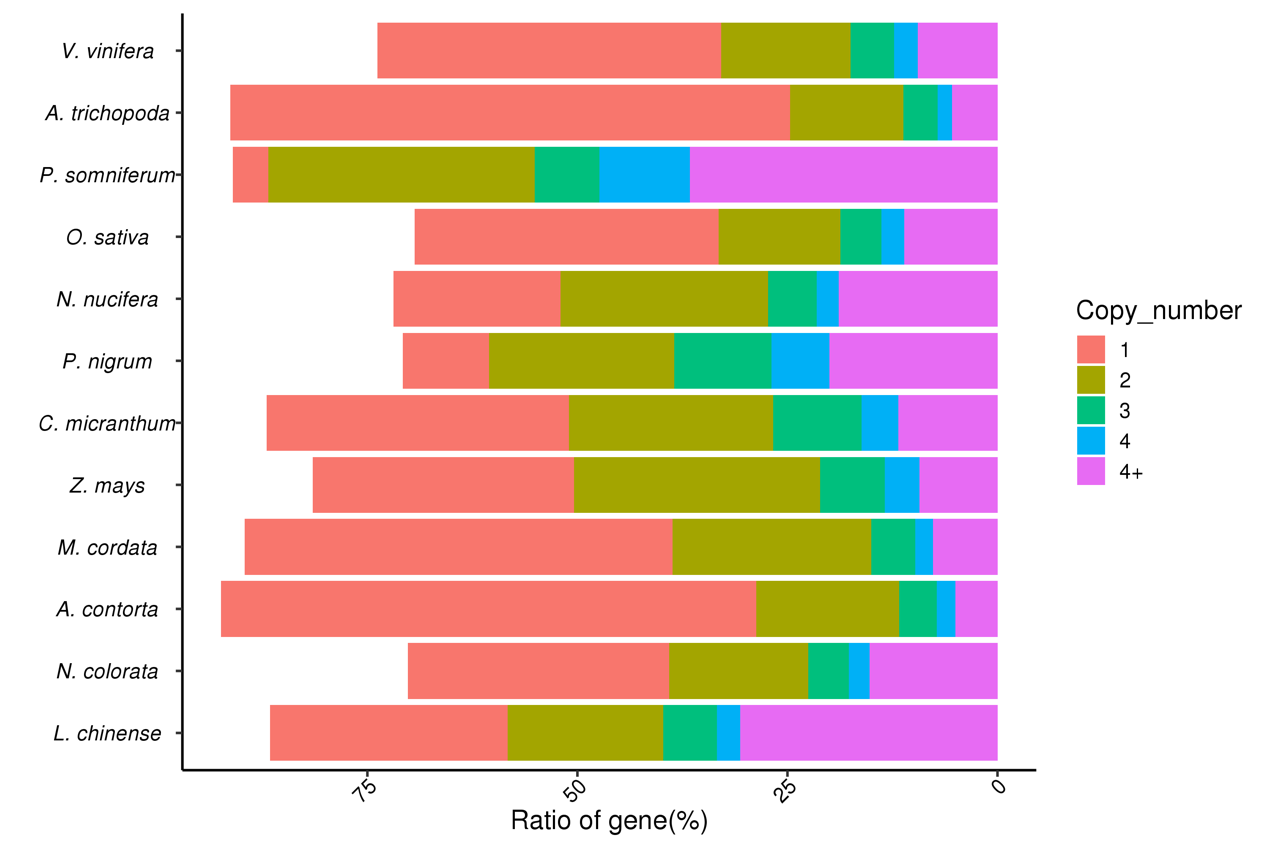
**

**Fig. S9 Gene copy number distribution in *A. contorta* and 11 other sequenced plant genomes.**


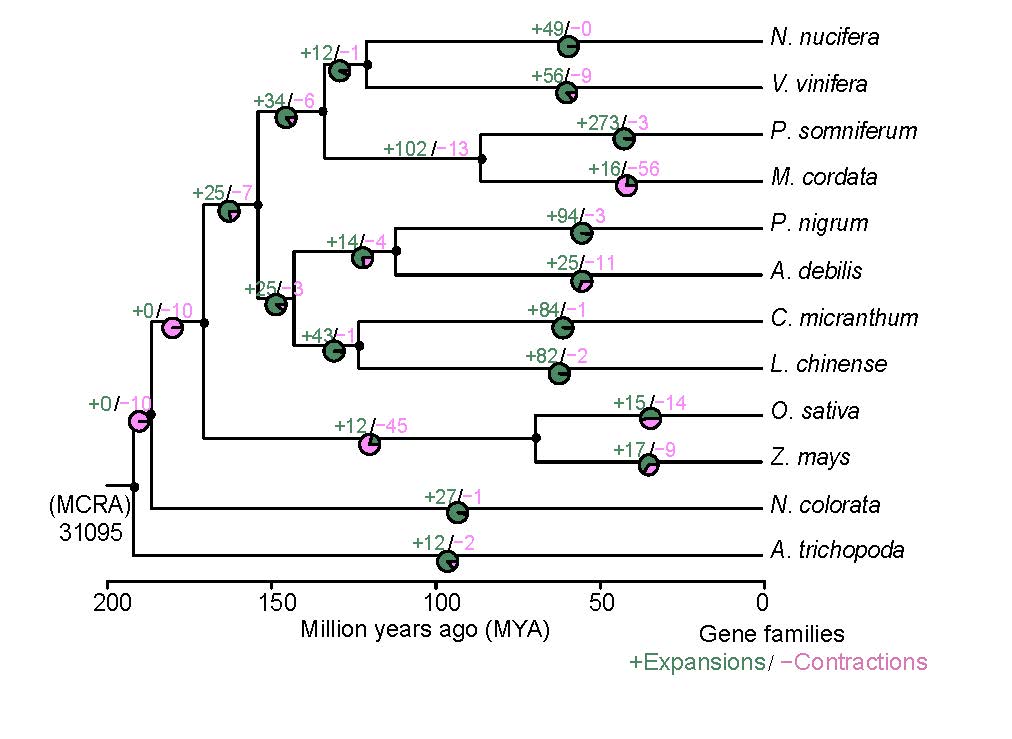


**Fig. S10 Phylogenetic tree with overview of the expansion and contraction of the gene family of 12 plant species.**


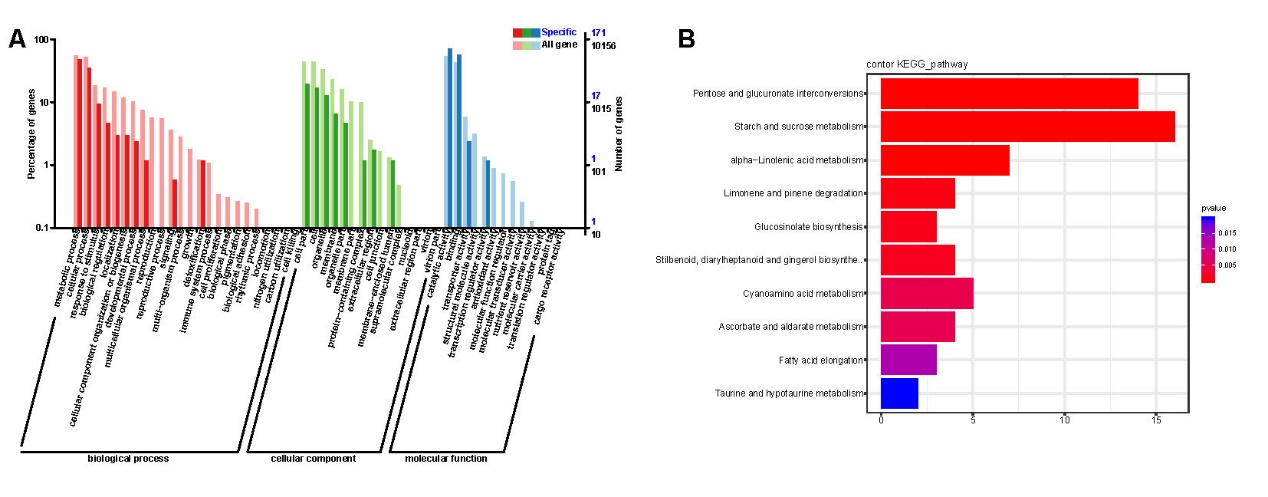


**Fig. S11 ﻿﻿A****(GO) and B(KEGG) annotation of *A. contorta* specific gene families.**
