# Supplementary material for: Chromosome-level genome assembly of Aristolochia contorta provides insights into the biosynthesis of benzylisoquinoline alkaloids and aristolochic acids
Source: Hortic Res. 2022 Feb 11;9:uhac005. doi: 10.1093/hr/uhac005 (PMC8973263; doi:10.1093/hr/uhac005)
Supplement: Web_Material_uhac005 [file web_material_uhac005.zip › FigS1-11-1014.pdf]

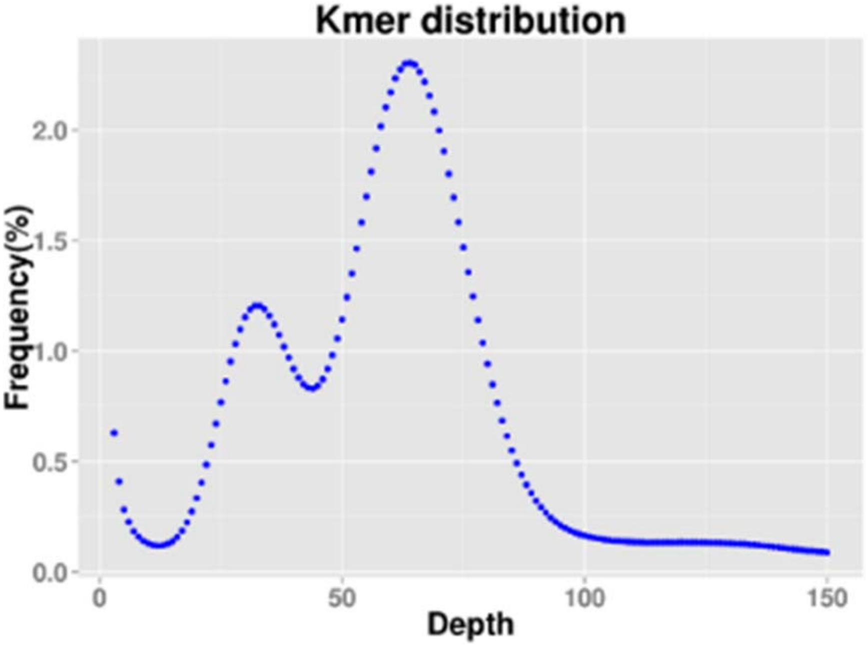

Fig. S1 Evaluation of *A. contorta* genome size by k-mer analysis (k=19).

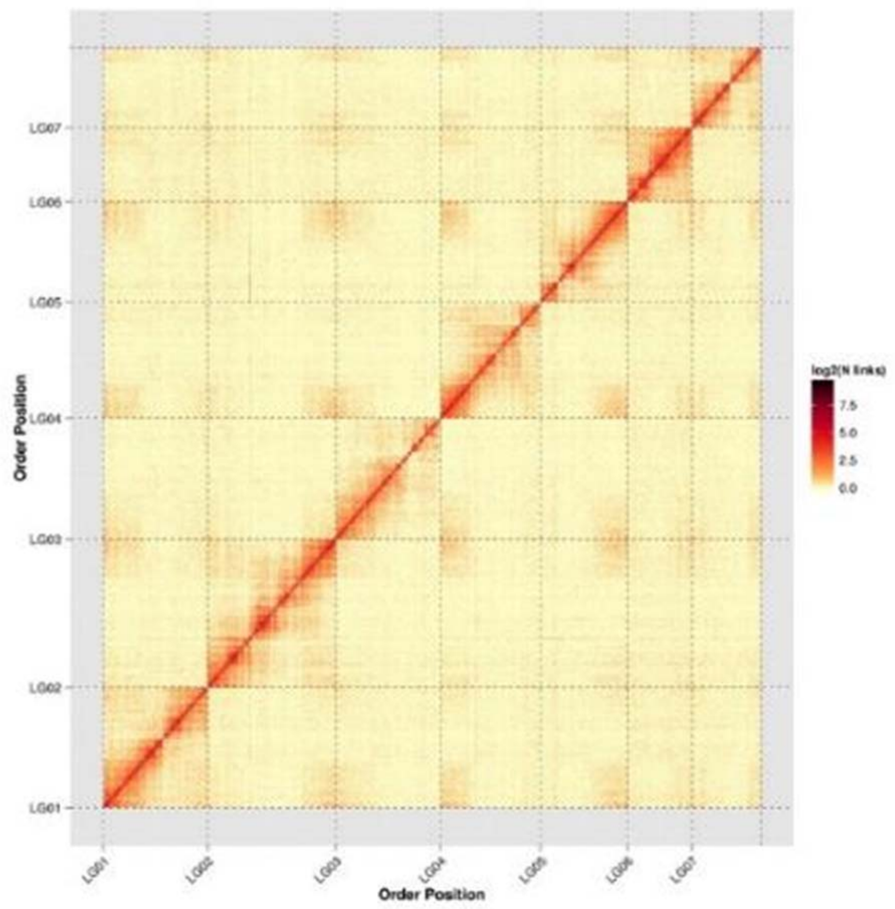

Fig. S2 Hi–C heat map of chromosome interactions. LG01–07 represent the 7 chromosomes. Both x-axis and y-axis represent the order of each bin on the corresponding chromosome group. The color of the bar on the right represents the strength of interaction.

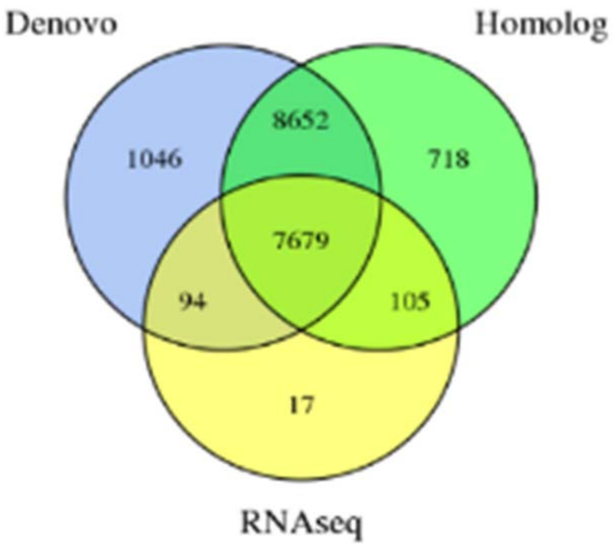

Fig. S3 Genes derived from the distribution map of the three prediction methods.

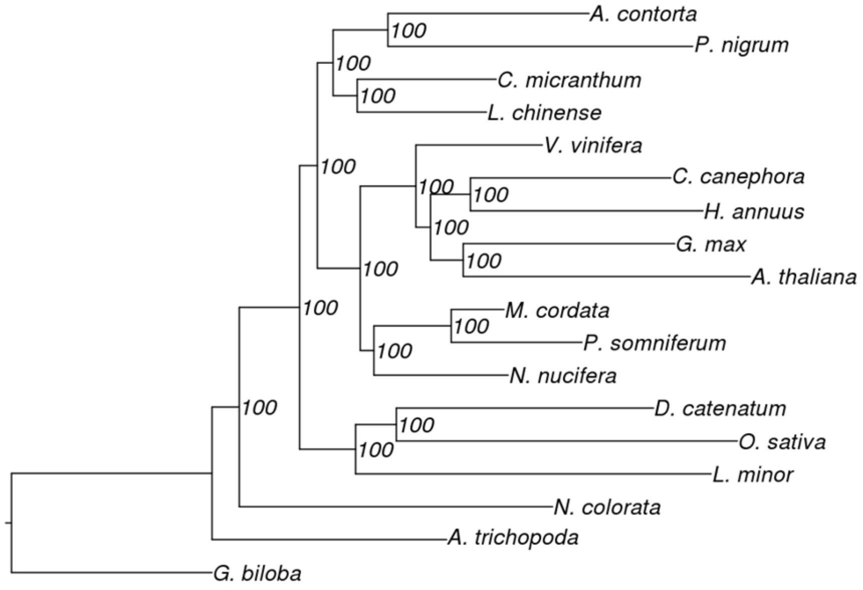

Fig. S4 Phylogenetic tree with bootstrap support values of each node among 18 plant species.

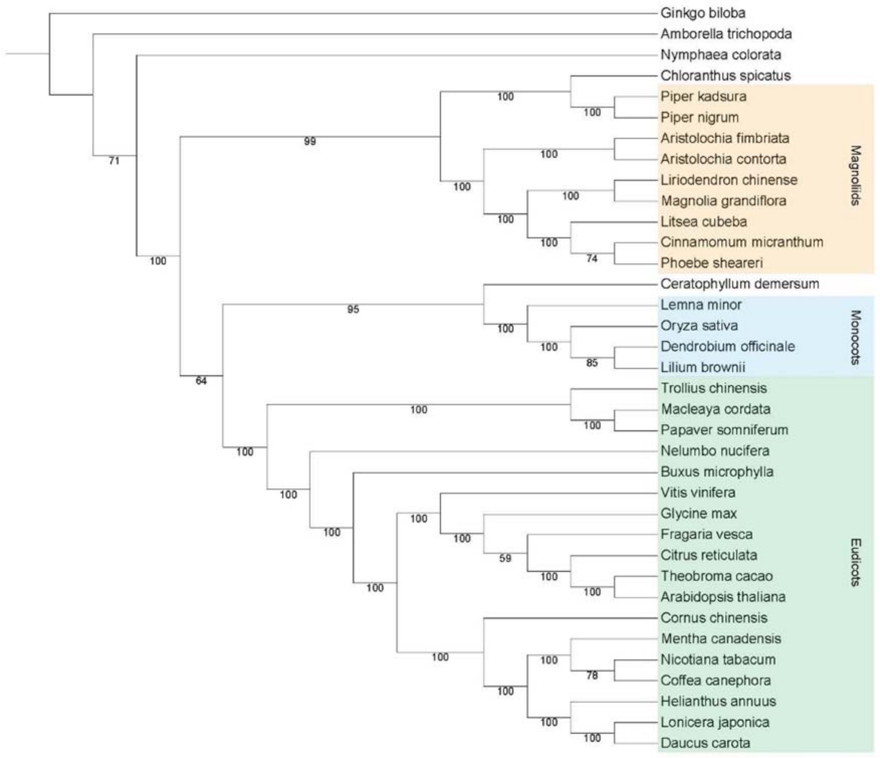

Fig. S5 Phylogenetic tree of chloroplast genomes among 36 species using ML method.

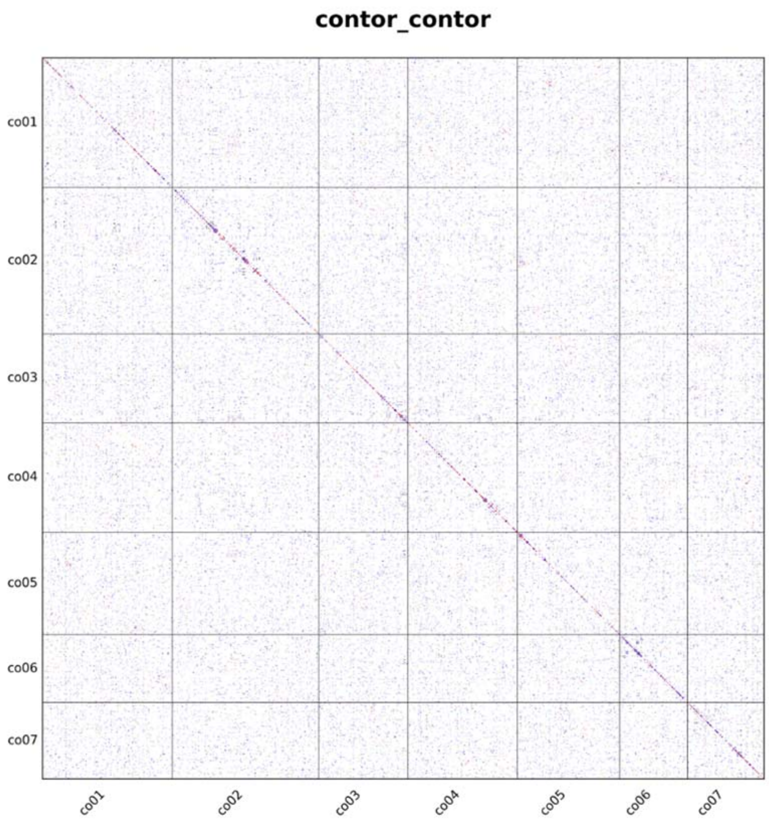

Fig. S6 The synteny of *A. contorta* paralogous gene pairs. The paralogs displayed by dot plot matrix in *A. contorta*.

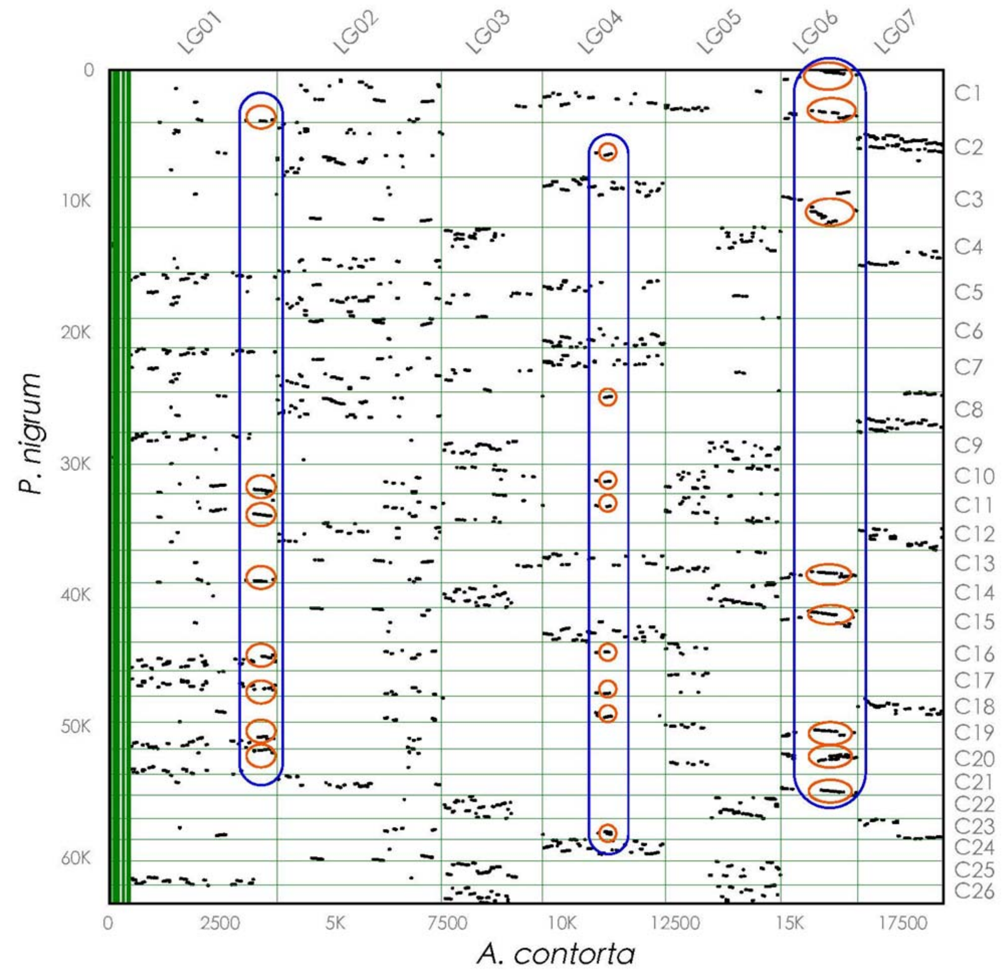

Fig. S7 Syntenic dotplot between the *A. contorta* and *P. nigrum* genomes

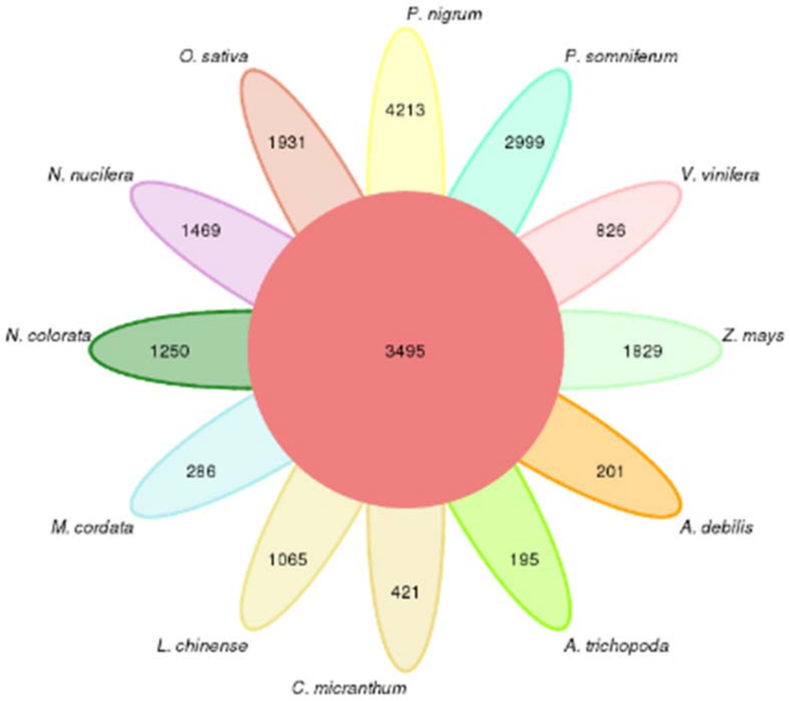

Fig. S8 Gene family cluster petal diagram.

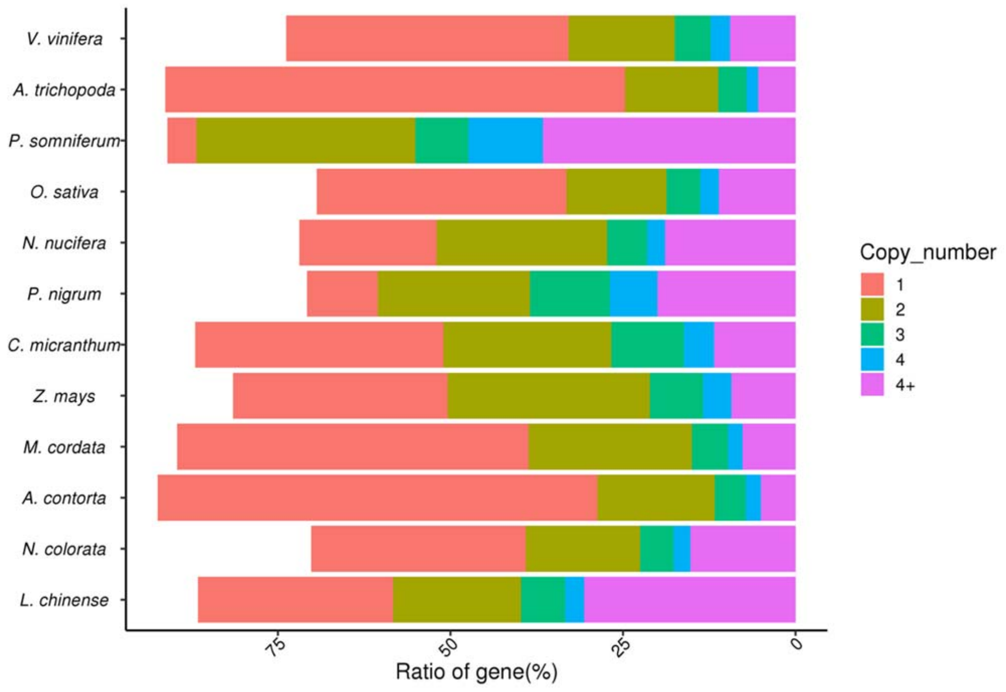

Fig. S9 Gene copy number distribution in *A. contorta* and 11 other sequenced plant genomes.

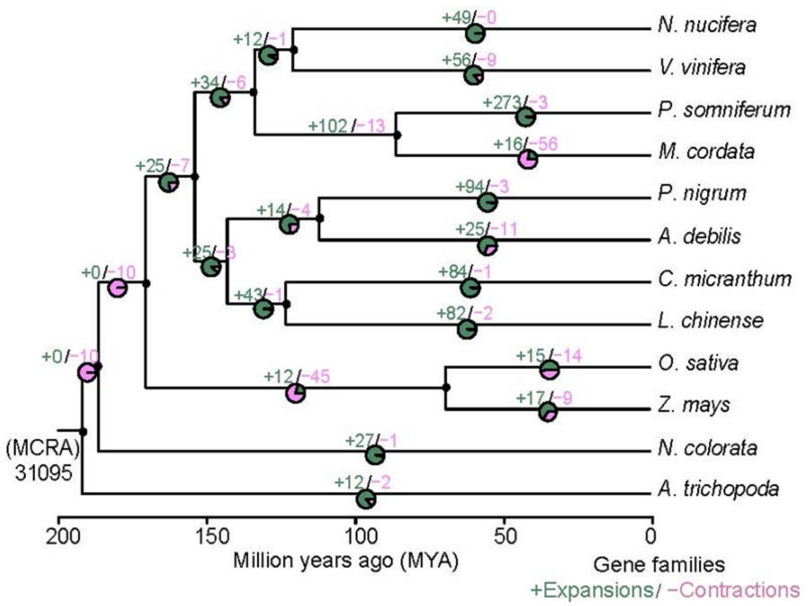

Fig. S10 Phylogenetic tree with overview of the expansion and contraction of the gene family of 12 plant species.

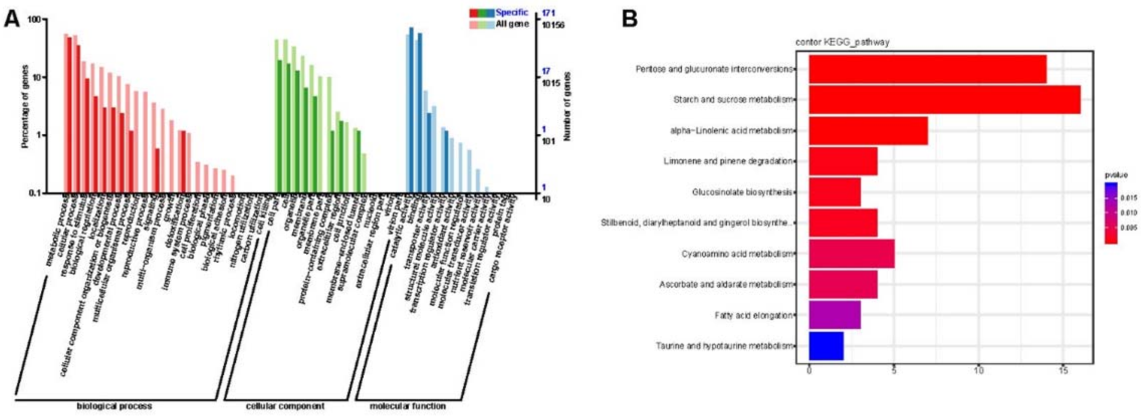

Fig. S11 A(GO) and B(KEGG) annotation of *A. contorta* specific gene families.
