# Supplementary material for: Chromosome-level genome assembly of Aristolochia contorta provides insights into the biosynthesis of benzylisoquinoline alkaloids and aristolochic acids
Source: Hortic Res. 2022 Feb 11;9:uhac005. doi: 10.1093/hr/uhac005 (PMC8973263; doi:10.1093/hr/uhac005)
Supplement: Web_Material_uhac005 [file web_material_uhac005.zip › FigS12-14.docx]

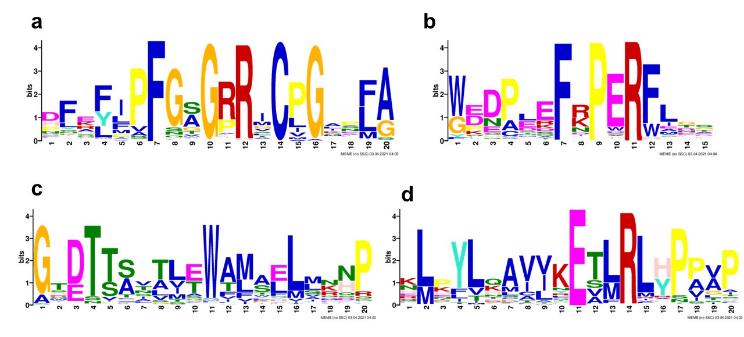


**Fig. S12 Weblogos of the conserved motifs identified in CYP proteins from *A. contorta*.** **a** heme-binding motif, **b** PERF motif, **c** I-helix region, **d** K-helix region.

**Fig. S13 Schematic representations of the *A. contorta* CYPs location on pseudochromosomes.** The picture was carried out by TBtools.

**Fig. S14 The expression patterns of *A. contorta* genes in roots, stems, leaves, and flowers examined by qRT-PCR.** X-axis represents the different tissues of A. contorta and y-axis represents the relative expression level for genes. Error bar indicates the standard deviation of three biological replicates.
