# Supplementary material for: Chromosome-level genome assembly of Aristolochia contorta provides insights into the biosynthesis of benzylisoquinoline alkaloids and aristolochic acids
Source: Hortic Res. 2022 Feb 11;9:uhac005. doi: 10.1093/hr/uhac005 (PMC8973263; doi:10.1093/hr/uhac005)
Supplement: Web_Material_uhac005 [file web_material_uhac005.zip › FigS15-18.docx]

**
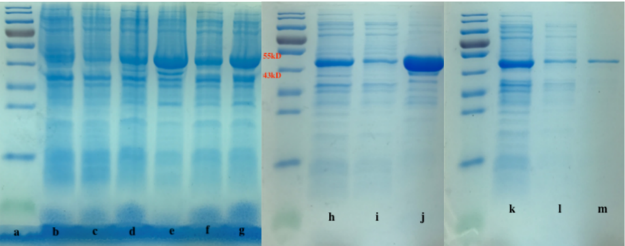
**

**Fig. S15 SDS-PAGE analysis of AcOMT1 and AcOMT2.**

Lane a: Marker; Lane b: Empty pET30a vector (uninduced); Lane c: Empty pET30a vector (induced); Lane d: pET30a- AcOMT2 (uninduced); Lane e/h: pET30a- AcOMT2 (induced); Lane f: pET30a- AcOMT1 (uninduced); Lane g/k: pET30a- AcOMT1 (induced); Lane i: ﻿Soluble AcOMT2; Lane j: Purified ﻿AcOMT2 protein; Lane l: ﻿Soluble AcOMT1; Lane j: Purified ﻿AcOMT1 protein.

**
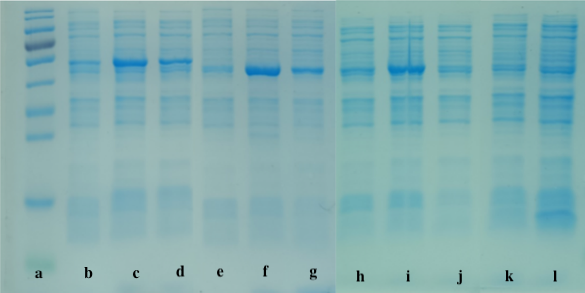
**

**Fig. S16 SDS-PAGE analysis of AcOMT3, AcOMT5 and AcOMT7.**

Lane a: Marker; Lane b: pET30a- AcOMT5 (uninduced); Lane c: pET30a- AcOMT5 (induced); Lane d: ﻿Soluble AcOMT5; Lane e: pET30a- AcOMT7 (uninduced); Lane f: pET30a- AcOMT7 (induced); Lane g: ﻿Soluble AcOMT7; Lane h: pET30a- AcOMT5 (uninduced); Lane i: pET30a- AcOMT5 (induced); Lane j: ﻿Soluble AcOMT5; Lane k: Empty pET30a vector (uninduced); Lane l: Empty pET30a vector (induced).

**
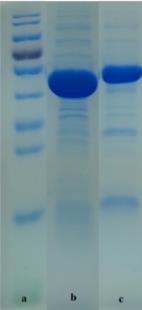
**

**Fig. S17 SDS-PAGE analysis of purified AcOMT5 and AcOMT7.**

Lane a: Marker; Lane b: purified AcOMT5; Lane c: purified AcOMT7.

**
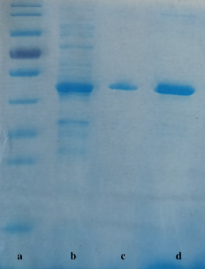
**

**Fig. S18 SDS-PAGE analysis of purified AcOMT3.**

Lane a: Marker; Lane b: pET30a- AcOMT3 (induced); Lane c: Soluble protein in 6M Guanidine hydrochloride. Lane d: Renatured protein.
