# Supplementary material for: Chromosome-level genome assembly of Aristolochia contorta provides insights into the biosynthesis of benzylisoquinoline alkaloids and aristolochic acids
Source: Hortic Res. 2022 Feb 11;9:uhac005. doi: 10.1093/hr/uhac005 (PMC8973263; doi:10.1093/hr/uhac005)
Supplement: Web_Material_uhac005 [file web_material_uhac005.zip › TableS1-13.docx]

| Table S1. Hi-C Assembly data statistics | |  |  |  |
| --- | --- | --- | --- | --- |
| **Group** | **Cluster Num** | **Cluster Len(bp)** | **Order Num** | **Order Len(bp)** |
| LG01 | 18 | 32,422,454 | 11 | 31,427,636 |
| LG02 | 42 | 39,786,356 | 31 | 38,740,436 |
| LG03 | 55 | 33,945,346 | 38 | 31,589,686 |
| LG04 | 29 | 31,661,893 | 22 | 30,379,727 |
| LG05 | 26 | 28,091,832 | 17 | 26,144,098 |
| LG06 | 17 | 19,748,185 | 14 | 19,487,744 |
| LG07 | 27 | 23,609,743 | 14 | 20,791,271 |
| Total(Ratio %) | 214(95.54) | 209265809(99.4) | 147(68.69) | 198560598(94.88) |

| Table S2. Post-Hi-C genome statistics | |
| --- | --- |
| Parameter | Number/Length |
| Scaffold number | 84 |
| Scaffold length (bp) | 210,540,769 |
| Scaffold N50 (bp) | 30,381,827 |
| Scaffold N90 (bp) | 19,489,044 |
| Scaffold max (bp) | 38,743,436 |
| Gap total length (bp) | 14,000 |
| Contig number | 224 |
| Contig length (bp) | 210,526,769 |
| Contig N50 (bp) | 2,315,928 |
| Contig N90 (bp) | 388,540 |
| Contig max (bp) | 12,320,100 |
| GC content (%) | 39.39 |

| Table S3. Validation of genome assembly using BUSCO and CEGMA method | | | | | |
| --- | --- | --- | --- | --- | --- |
| **Species** | **Complete BUSCOs** | **Complete and single-copy BUSCOs** | **Complete and duplicated BUSCOs** | **Fragmented BUSCOs** | **Missing BUSCOs** |
| *A. contorta* | 1,300 (90.28%) | 1,233 (85.62%) | 67 (4.65%) | 41 (2.85%) | 99 (6.88%) |
|  |  |  |  |  |  |
| **Species** | **Number of 458 CEGs* present in assembly** | **% of 458 CEGs present in assemblies** | **Number of 248 highly conserved CEGs present** | **% of 248 highly conserved CEGs present** | |
| *A. contorta* | 446 | 97.38% | 224 | 90.32% |  |

| Table S4. Repeat sequence statistics | | |  |
| --- | --- | --- | --- |
| Type | **Number** | **Length** | **Rate(%)** |
| ClassI | 130,737 | 58,442,626 | 27.76 |
| ClassI/DIRS | 8,025 | 4,477,176 | 2.13 |
| ClassI/LINE | 19,091 | 10,292,880 | 4.89 |
| ClassI/LTR | 392 | 190,944 | 0.09 |
| ClassI/LTR/Copia | 44,815 | 21,996,014 | 10.45 |
| ClassI/LTR/Gypsy | 18,389 | 10,867,633 | 5.16 |
| ClassI/PLE\|LARD | 35,272 | 13,382,265 | 6.36 |
| ClassI/SINE | 2,453 | 420,386 | 0.2 |
| ClassI/SINE\|TRIM | 38 | 6,621 | 0 |
| ClassI/TRIM | 1,030 | 290,407 | 0.14 |
| ClassI/Unknown | 1,232 | 336,302 | 0.16 |
| ClassII | 25,054 | 9,546,083 | 4.53 |
| ClassII/Crypton | 1 | 49 | 0 |
| ClassII/Helitron | 399 | 32,036 | 0.02 |
| ClassII/MITE | 1,292 | 201,801 | 0.1 |
| ClassII/Maverick | 2,045 | 550,014 | 0.26 |
| ClassII/TIR | 19,832 | 8,698,883 | 4.13 |
| ClassII/Unknown | 1,485 | 131,631 | 0.06 |
| PotentialHostGene | 8,446 | 2,394,407 | 1.14 |
| Total | 165,811 | 80,555,983 | 38.26 |

| Table S5. Statistical of gene prediction | | | |
| --- | --- | --- | --- |
| **Method** | **Software** | **Species** | **Gene number** |
| Ab initio | Genscan | - | 19,156 |
|  | Augustus | - | 13,113 |
|  | GlimmerHMM | - | 22,245 |
|  | GeneID | - | 30,495 |
|  | SNAP | - | 25,902 |
| Homology-based | GeMoMa | Arabidopsis_thaliana | 16,226 |
|  |  | Oryza sativa Japonica | 15,903 |
|  |  | Cinnamomum kanehirae | 16,642 |
|  |  |  |  |
|  |  | Olea europaea | 19,790 |
|  |  |  |  |
| RNAseq | PASA | - | 8,839 |
| Integration | EVM | - | 18,311 |

Table S6. Statistical of genetic information

| Parameter | Value |
| --- | --- |
| Gene Num | 18,311 |
| GeneLen (bp) | 80,906,219 |
| AveGeneLen (bp) | 4,418 |
| ExonLen (bp) | 27,945,818 |
| AveExonLen(bp) | 1526 |
| IntronLen (bp) | 52,960,401 |
| AveIntronLen(bp) | 2,892 |

Table S7. Founctional analysis

| Parameter | Number | Percentage (%) |
| --- | --- | --- |
| Total repetitive sequences | 165,811 | 38.26 |
| Protein-coding genes | 18,311 | / |
| rRNA | 404 | / |
| tRNA | 475 | / |
| miRNA | 46 | / |
| Pseudogenes | 770 | / |
| All functional annotations | 17,765 | 97.02% |
| GO_annotations | 10,156 | 55.46% |
| KEGG_annotations | 6,703 | 36.61% |
| KOG_annotations | 10,553 | 57.63% |
| TrEMBL_annotations | 17,732 | 96.84% |
| Nr_annotations | 17,749 | 96.93% |
| Unannotated | 546 | 2.98% |

| Table S8. Characteristics of full-length CYP450s of *A. contorta* identified. | | | | |
| --- | --- | --- | --- | --- |
| Gene ID | Type | CYP Clan | CYP Family | CYP Subfamily |
| EVM0014314 | A | 71 | CYP71 | CYP71A |
| EVM0016207 | A | 71 | CYP71 | CYP71D |
| EVM0009965 | A | 71 | CYP71 | CYP71A |
| EVM0014133 | A | 71 | CYP71 |  |
| EVM0001806 | A | 71 | CYP71 | CYP71D |
| EVM0004015 | A | 71 | CYP71 |  |
| EVM0011884 | A | 71 | CYP71 | CYP71D |
| EVM0011970 | A | 71 | CYP71 | CYP71D |
| EVM0016622 | A | 71 | CYP71 | CYP71A |
| EVM0009150 | A | 71 | CYP71 | CYP71A |
| EVM0004982 | A | 71 | CYP71 | CYP71D |
| EVM0000777 | A | 71 | CYP71 |  |
| EVM0013011 | A | 71 | CYP71 | CYP71D |
| EVM0004726 | A | 71 | CYP71 |  |
| EVM0003623 | A | 71 | CYP71 |  |
| EVM0006325 | A | 71 | CYP71 | CYP71D |
| EVM0006200 | A | 71 | CYP71 | CYP71D |
| EVM0000692 | A | 71 | CYP92 |  |
| EVM0016570 | A | 71 | CYP71 |  |
| EVM0003085 | A | 71 | CYP71 |  |
| EVM0005024 | A | 71 | CYP71 |  |
| EVM0002075_1 | A | 71 | CYP93 | CYP93A |
| EVM0002075_2 | A | 71 | CYP93 | CYP93A |
| EVM0008943 | A | 71 | CYP71 | CYP71A |
| EVM0016545 | A | 71 | CYP71 | CYP71D |
| EVM0007823 | A | 71 | CYP71 |  |
| EVM0017651 | A | 71 | CYP736 | CYP736A |
| EVM0010960 | A | 71 | CYP76 |  |
| EVM0009341 | A | 71 | CYP76 |  |
| EVM0010458 | A | 71 | CYP71 | CYP71D |
| EVM0005055 | A | 71 | CYP71 |  |
| EVM0006204 | A | 71 | CYP93 | CYP93A |
| EVM0001459 | A | 71 | CYP76 | CYP76F |
| EVM0007937 | A | 71 | CYP76 | CYP76F |
| EVM0001519 | A | 71 | CYP706 |  |
| EVM0009949 | A | 71 | CYP84 | CYP84A |
| EVM0007442 | A | 71 | CYP84 | CYP84A |
| EVM0000033 | A | 71 | CYP76 | CYP76F |
| EVM0018211 | A | 71 | CYP76 |  |
| EVM0017918 | A | 71 | CYP76 |  |
| EVM0017445 | A | 71 | CYP71 |  |
| EVM0016340 | A | 71 | CYP76 |  |
| EVM0004815 | A | 71 | CYP76 |  |
| EVM0008552 | A | 71 | CYP76 |  |
| EVM0008759 | A | 71 | CYP706 |  |
| EVM0015272 | A | 71 | CYP706 |  |
| EVM0004747 | A | 71 | CYP706 |  |
| EVM0007827 | A | 71 | CYP706 |  |
| EVM0000865 | A | 71 | CYP703 | CYP703A |
| EVM0002446 | A | 71 | CYP706 |  |
| EVM0008424 | A | 71 | CYP98 | CYP98A |
| EVM0008104_1 | A | 71 | CYP81 | CYP81Q |
| EVM0008104_2 | A | 71 | CYP81 | CYP81Q |
| EVM0018306 | A | 71 | CYP706 |  |
| EVM0005076 | A | 71 | CYP706 |  |
| EVM0006218 | A | 71 | CYP706 |  |
| EVM0009330 | A | 71 | CYP82 | CYP82C |
| EVM0001383 | A | 71 | CYP93 |  |
| EVM0012200 | A | 71 | CYP80 |  |
| EVM0018114 | A | 71 | CYP82 | CYP82C |
| EVM0006719 | A | 71 | CYP82 | CYP82C |
| EVM0004889 | A | 71 | CYP706 |  |
| EVM0015053 | A | 71 | CYP82 | CYP82C |
| EVM0005067 | A | 71 | CYP706 |  |
| EVM0010280 | A | 71 | CYP78 | CYP78A |
| EVM0014042 | A | 71 | CYP706 |  |
| EVM0004610 | A | 71 | CYP82 |  |
| EVM0001111 | A | 71 | CYP82 |  |
| EVM0016112 | A | 71 | CYP75 | CYP75B |
| EVM0001440 | A | 71 | CYP706 | CYP706 |
| EVM0009551 | A | 71 | CYP82 | CYP82C |
| EVM0005670 | A | 71 | CYP82 | CYP82C |
| EVM0018090 | A | 71 | CYP71 |  |
| EVM0000405 | A | 71 | CYP82 | CYP82C |
| EVM0006780 | A | 71 | CYP706 |  |
| EVM0013435 | A | 71 | CYP706 |  |
| EVM0012438 | A | 71 | CYP82 |  |
| EVM0003486 | A | 71 | CYP82 | CYP82C |
| EVM0004485 | A | 71 | CYP82 |  |
| EVM0000695 | A | 71 | CYP71 |  |
| EVM0006589 | A | 71 | CYP71 |  |
| EVM0009476 | A | 71 | CYP82 |  |
| EVM0017491 | A | 71 | CYP82 |  |
| EVM0001635 | A | 71 | CYP80 |  |
| EVM0007338 | A | 71 | CYP82 |  |
| EVM0007794 | A | 71 | CYP82 |  |
| EVM0004589 | A | 71 | CYP82 | CYP82C |
| EVM0006707 | A | 71 | CYP706 |  |
| EVM0009697 | A | 71 | CYP98 | CYP98A |
| EVM0013783 | A | 71 | CYP706 |  |
| EVM0008748 | A | 71 | CYP82 |  |
| EVM0000886 | A | 71 | CYP82 |  |
| EVM0017761 | A | 71 | CYP82 | CYP82C |
| EVM0013344 | A | 71 | CYP706 |  |
| EVM0008901 | A | 71 | CYP80 | CYP80G |
| EVM0017004 | A | 71 | CYP82 |  |
| EVM0002629 | A | 71 | CYP81 |  |
| EVM0011795 | A | 71 | CYP706 |  |
| EVM0002156 | A | 71 | CYP82 | CYP82C |
| EVM0002703 | A | 71 | CYP80 | CYP80B |
| EVM0009777 | A | 71 | CYP706 |  |
| EVM0008024 | A | 71 | CYP81 |  |
| EVM0005930 | A | 71 | CYP82 | CYP82C |
| EVM0000505 | A | 71 | CYP706 |  |
| EVM0000340 | A | 71 | CYP82 | CYP82C |
| EVM0001287 | A | 71 | CYP78 | CYP78A |
| EVM0014504 | A | 71 | CYP78 | CYP78A |
| EVM0012792 | A | 71 | CYP82 |  |
| EVM0010384 | A | 71 | CYP706 |  |
| EVM0010174 | A | 71 | CYP706 |  |
| EVM0016799 | A | 71 | CYP82 | CYP82C |
| EVM0000480 | A | 71 | CYP706 |  |
| EVM0012519 | A | 71 | CYP82 |  |
| EVM0002746 | A | 71 | CYP706 |  |
| EVM0011802 | A | 71 | CYP82 |  |
| EVM0002489 | A | 71 | CYP78 | CYP78A |
| EVM0009511 | A | 71 | CYP73 | CYP73A |
| EVM0005040 | A | 71 | CYP706 |  |
| EVM0002723 | A | 71 | CYP71 |  |
| EVM0007706 | A | 71 | CYP706 |  |
| EVM0005523 | A | 71 | CYP82 |  |
| EVM0011934 | A | 71 | CYP80 |  |
| EVM0017664 | A | 71 | CYP89 | CYP89A |
| EVM0013208 | A | 71 | CYP706 |  |
| EVM0001794 | A | 71 | CYP706 |  |
| EVM0003110_1 | A | 71 | CYP89 | CYP89A |
| EVM0003110_2 | A | 71 | CYP89 |  |
| EVM0003110_3 | A | 71 | CYP89 | CYP89A |
| EVM0013609 | A | 71 | CYP79 | CYP79A |
| EVM0000133 | A | 71 | CYP82 |  |
| EVM0013068 | A | 71 | CYP706 |  |
| EVM0007461 | A | 71 | CYP82 | CYP82C |
| EVM0004494 | A | 71 | CYP719 |  |
| EVM0017199 | A | 71 | CYP77 | CYP77B |
| EVM0011707 | A | 71 | CYP706 |  |
| EVM0005435 | A | 71 | CYP706 |  |
| EVM0010452 | A | 71 | CYP79 | CYP79A |
| EVM0016237 | A | 71 | CYP79 | CYP79A |
| EVM0011985 | A | 71 | CYP79 | CYP79A |
| EVM0011393 | A | 71 | CYP77 | CYP77A |
| EVM0008616 | A | 71 | CYP701 | CYP701A |
| EVM0005442 | A | 71 | CYP79 | CYP79A |
| EVM0002942 | A | 71 | CYP79 | CYP79A |
| EVM0008747 | A | 71 | CYP79 | CYP79A |
| EVM0006852 | A | 71 | CYP706 |  |
| EVM0012874 | A | 71 | CYP79 | CYP79A |
| EVM0015787 | A | 71 | CYP706 |  |
| EVM0011719 | A | 71 | CYP76 |  |
| EVM0011218 | A | 71 | CYP79 |  |
| EVM0001119 | A | 71 | CYP706 |  |
| EVM0010225 | A | 71 | CYP89 |  |
| EVM0015757 | A | 71 | CYP82 |  |
| EVM0000266 | A | 71 | CYP706 |  |
| EVM0006725 | A | 71 | CYP706 |  |
| EVM0007439 | A | 71 | CYP706 |  |
| EVM0016865_1 | non-A | 72 | CYP72 | CYP72A |
| EVM0016865_2 | non-A | 72 | CYP72 | CYP72A |
| EVM0017387 | non-A | 72 | CYP72 | CYP72A |
| EVM0016899 | non-A | 72 | CYP72 | CYP72A |
| EVM0008717 | non-A | 72 | CYP72 | CYP72A |
| EVM0014026 | non-A | 72 | CYP72 | CYP72A |
| EVM0004920 | non-A | 72 | CYP72 | CYP72A |
| EVM0007454 | non-A | 72 | CYP72 | CYP72A |
| EVM0016830 | non-A | 72 | CYP72 | CYP72A |
| EVM0012251 | non-A | 72 | CYP72 | CYP72A |
| EVM0012555 | non-A | 72 | CYP734 | CYP734A |
| EVM0011493 | non-A | 72 | CYP72 | CYP72A |
| EVM0007334 | non-A | 72 | CYP721 | CYP721A |
| EVM0011281 | non-A | 72 | CYP721 | CYP721A |
| EVM0004562 | non-A | 72 | CYP735 | CYP735A |
| EVM0002491 | non-A | 72 | CYP709 | CYP709B |
| EVM0003857 | non-A | 72 | CYP709 | CYP709B |
| EVM0005381 | non-A | 72 | CYP709 | CYP709B |
| EVM0003097 | non-A | 72 | CYP715 |  |
| EVM0017376 | non-A | 72 | CYP714 |  |
| EVM0001498_1 | non-A | 72 | CYP714 |  |
| EVM0015687 | non-A | 72 | CYP714 | CYP714C |
| EVM0001498_2 | non-A | 72 | CYP714 |  |
| EVM0004011 | non-A | 72 | CYP714 |  |
| EVM0010168 | non-A | 72 | CYP714 |  |
| EVM0014128 | non-A | 72 | CYP714 |  |
| EVM0002410 | non-A | 72 | CYP714 |  |
| EVM0008936 | non-A | 74 | CYP74 | CYP74A |
| EVM0008472 | non-A | 74 | CYP74 | CYP74B |
| EVM0011703 | non-A | 85 | CYP85 | CYP85A |
| EVM0006238 | non-A | 85 | CYP85 | CYP85A |
| EVM0009290 | non-A | 85 | CYP90 | CYP90D |
| EVM0005573 | non-A | 85 | CYP87 | CYP87A |
| EVM0002089 | non-A | 85 | CYP90 | CYP90A |
| EVM0013807 | non-A | 85 | CYP90 | CYP90B |
| EVM0007709 | non-A | 85 | CYP87 | CYP87A |
| EVM0010503 | non-A | 85 | CYP87 | CYP87A |
| EVM0010777 | non-A | 85 | CYP724 | CYP724B |
| EVM0005890 | non-A | 85 | CYP720 | CYP720A |
| EVM0010408 | non-A | 85 | CYP724 | CYP724B |
| EVM0004053 | non-A | 85 | CYP724 | CYP724B |
| EVM0016312 | non-A | 85 | CYP707 | CYP707A |
| EVM0017535 | non-A | 85 | CYP707 | CYP707A |
| EVM0012763 | non-A | 85 | CYP729 |  |
| EVM0009910 | non-A | 85 | CYP707 | CYP707A |
| EVM0005736 | non-A | 85 | CYP716 | CYP716B |
| EVM0008283 | non-A | 85 | CYP90 | CYP90C |
| EVM0005688 | non-A | 85 | CYP88 | CYP88A |
| EVM0001010 | non-A | 85 | CYP90 |  |
| EVM0010938 | non-A | 85 | CYP733 |  |
| EVM0005940 | non-A | 85 | CYP716 | CYP716B |
| EVM0016151 | non-A | 85 | CYP722 |  |
| EVM0002396 | non-A | 85 | CYP722 |  |
| EVM0010801 | non-A | 86 | CYP86 | CYP86A |
| EVM0014530 | non-A | 86 | CYP86 | CYP86A |
| EVM0004350 | non-A | 86 | CYP86 | CYP86B |
| EVM0017114 | non-A | 86 | CYP86 | CYP86C |
| EVM0016761 | non-A | 86 | CYP94 | CYP94A |
| EVM0017500 | non-A | 86 | CYP94 | CYP94B |
| EVM0010671_1 | non-A | 86 | CYP94 | CYP94A |
| EVM0010671_2 | non-A | 86 | CYP94 | CYP94A |
| EVM0009397 | non-A | 86 | CYP94 | CYP94B |
| EVM0017164 | non-A | 86 | CYP94 | CYP94B |
| EVM0004697 | non-A | 86 | CYP94 | CYP94B |
| EVM0002442 | non-A | 86 | CYP94 | CYP94B |
| EVM0011679 | non-A | 86 | CYP94 | CYP94B |
| EVM0017670 | non-A | 86 | CYP94 | CYP94C |
| EVM0007829_1 | non-A | 86 | CYP96 |  |
| EVM0001329 | non-A | 86 | CYP96 |  |
| EVM0001396 | non-A | 86 | CYP96 |  |
| EVM0004731 | non-A | 86 | CYP96 |  |
| EVM0008867 | non-A | 86 | CYP704 | CYP704A |
| EVM0011064 | non-A | 86 | CYP96 |  |
| EVM0001231 | non-A | 86 | CYP96 |  |
| EVM0007829_2 | non-A | 86 | CYP96 |  |
| EVM0007996 | non-A | 86 | CYP96 |  |
| EVM0005263 | non-A | 86 | CYP704 | CYP704A |
| EVM0017105 | non-A | 86 | CYP96 |  |
| EVM0009878 | non-A | 86 | CYP704 | CYP704B |
| EVM0003066 | non-A | 97 | CYP97 | CYP97A |
| EVM0012894 | non-A | 97 | CYP97 | CYP97C |
| EVM0007861 | non-A | 97 | CYP97 | CYP97B |
| EVM0014428 | non-A | 710 | CYP710 | CYP710A |
| EVM0008671 | non-A | 711 | CYP711 | CYP711A |
| EVM0011753 | non-A | 727 | CYP727 |  |
| EVM0010488 | non-A | 51 | CYP51 | CYP51G |

**Table. S9 BIAs and its derivatives in metabolome profiling results**

| ﻿NO. | ﻿Metabolites | ﻿Formula | Structure |
| --- | --- | --- | --- |
| 1 | Reticuline | C19H23NO4 |  |
| 2 | Cepharadione A | C18H11NO4 |  |
| 3 | *N*-Methylcoclaurine | C18H21NO3 |  |
| 4 | Secoisotetrandrine | C38H40N2O8 |  |
| 5 | Norcorydine | C19H21NO4 |  |
| 6 | Isocorydine | C20H23NO4 |  |
| 7 | *O*-Methylarmepavine | C20H25NO3 |  |
| 8 | Annocherine A | C17H15NO4 |  |
| 9 | Annocherine B | C18H17NO4 |  |
| 10 | *N*-Acetylanonaine | C19H17NO3 |  |
| 11 | Anonaine | C17H15NO2 |  |
| 12 | Aristolodione | C18H13NO4 |  |
| 13 | Laurelliptine | C18H19NO4 |  |
| 14 | 6-Acetylmorphine | C19H21NO4 |  |
| 15 | Genomorphine | C17H19NO4 |  |
| 16 | Morphine | C17H19NO3 |  |
| 17 | Nandigerine | C18H17NO4 |  |
| 18 | Norsanguinarine | C19H11NO4 |  |
| 19 | Oxonantenine | C19H13NO5 |  |
| 20 | Noscapine | C22H23NO7 |  |
| 21 | Protopine | C20H19NO5 |  |
| 22 | Morphinan | C19H21NO3 |  |
| 23 | Dehydroaporheine | C18H15NO2 |  |
| 24 | 3'-Hydroxy-*N*-Methylcoclaurine | C18H21NO4 |  |

Table S10. 91 predicted nonredundant genes that were probably involved in the biosynthesis of BIAs in *A. contorta.*

| query ID | subject ID | species | putative function | identity | alignment length | mismatches | gap openings | q. start | q. end | s. start | s. end | e-value | bit score |
| --- | --- | --- | --- | --- | --- | --- | --- | --- | --- | --- | --- | --- | --- |
| O82415.2 | EVM0009250.1 | Papaver somniferum | TYDC | 77.255 | 510 | 110 | 2 | 1 | 510 | 1 | 504 | 0 | 838 |
| O82415.2 | EVM0004514.1 | Papaver somniferum | TYDC | 60.041 | 493 | 183 | 3 | 19 | 511 | 91 | 569 | 0 | 654 |
| O82415.2 | EVM0008727.1 | Papaver somniferum | TYDC | 55.212 | 518 | 220 | 4 | 1 | 510 | 1 | 514 | 0 | 613 |
| ADC33123.1 | EVM0014610.1 | Papaver somniferum | TyrAT | 62.103 | 409 | 151 | 2 | 1 | 407 | 1 | 407 | 0 | 525 |
| OVA00542.1 | EVM0006564.1 | Macleaya cordata | 3OHase | 61.663 | 433 | 139 | 6 | 4 | 411 | 1 | 431 | 0 | 532 |
| OVA00542.1 | EVM0013678.1 | Macleaya cordata | 3OHase | 66.116 | 363 | 116 | 5 | 55 | 411 | 60 | 421 | 1.10E-171 | 494 |
| OVA00542.1 | EVM0017300.1 | Macleaya cordata | 3OHase | 49.068 | 322 | 153 | 7 | 96 | 411 | 82 | 398 | 9.91E-106 | 324 |
| OVA00542.1 | EVM0015372.1 | Macleaya cordata | 3OHase | 47.091 | 361 | 168 | 12 | 66 | 411 | 76 | 428 | 1.70E-103 | 313 |
| OVA00542.1 | EVM0005329.1 | Macleaya cordata | 3OHase | 45.257 | 369 | 176 | 12 | 63 | 411 | 95 | 457 | 6.23E-91 | 287 |
| OVA02291.1 | EVM0004514.1 | Macleaya cordata | 4HPPDC | 77.686 | 484 | 108 | 0 | 36 | 519 | 86 | 569 | 0 | 810 |
| OVA02291.1 | EVM0009250.1 | Macleaya cordata | 4HPPDC | 58.98 | 490 | 193 | 1 | 39 | 520 | 17 | 506 | 0 | 633 |
| OVA02291.1 | EVM0008727.1 | Macleaya cordata | 4HPPDC | 53.333 | 495 | 218 | 2 | 39 | 520 | 22 | 516 | 0 | 565 |
| Q4QTJ2.1 | EVM0001864.1 | Papaver somniferum | NCS1 | 38.71 | 155 | 88 | 4 | 39 | 190 | 1 | 151 | 7.33E-30 | 108 |
| Q4QTJ2.1 | EVM0010147.1 | Papaver somniferum | NCS1 | 37.419 | 155 | 90 | 4 | 39 | 190 | 1 | 151 | 2.42E-28 | 104 |
| Q4QTJ2.1 | EVM0005050.1 | Papaver somniferum | NCS1 | 37.87 | 169 | 93 | 5 | 30 | 193 | 148 | 309 | 8.09E-26 | 101 |
| Q4QTJ2.1 | EVM0014712.1 | Papaver somniferum | NCS1 | 35.714 | 154 | 94 | 3 | 39 | 190 | 1 | 151 | 1.87E-25 | 97.1 |
| Q4QTJ2.1 | EVM0011227.1 | Papaver somniferum | NCS1 | 35.065 | 154 | 95 | 3 | 39 | 190 | 1 | 151 | 3.26E-24 | 93.6 |
| Q4QTJ2.1 | EVM0011298.1 | Papaver somniferum | NCS1 | 35.2 | 125 | 78 | 3 | 48 | 171 | 50 | 172 | 3.72E-22 | 89.4 |
| Q4QTJ2.1 | EVM0000274.1 | Papaver somniferum | NCS1 | 33.766 | 154 | 87 | 5 | 10 | 163 | 144 | 282 | 1.78E-19 | 84.3 |
| Q4QTJ2.1 | EVM0007329.1 | Papaver somniferum | NCS1 | 33.766 | 154 | 87 | 5 | 10 | 163 | 144 | 282 | 2.25E-19 | 84.3 |
| Q4QTJ2.1 | EVM0012460.1 | Papaver somniferum | NCS1 | 34.167 | 120 | 71 | 4 | 79 | 192 | 3 | 120 | 1.97E-15 | 70.1 |
| Q6WUC1.1 | EVM0008621.1 | Papaver somniferum | 6OMT | 54.802 | 354 | 149 | 6 | 1 | 346 | 1 | 351 | 1.27E-140 | 402 |
| Q6WUC1.1 | EVM0011328.1 | Papaver somniferum | 6OMT | 53.803 | 355 | 152 | 7 | 1 | 346 | 1 | 352 | 1.11E-134 | 387 |
| Q6WUC1.1 | EVM0008782.1 | Papaver somniferum | 6OMT | 46.957 | 345 | 171 | 7 | 12 | 346 | 29 | 371 | 1.96E-121 | 353 |
| Q6WUC1.1 | EVM0014836.1 | Papaver somniferum | 6OMT | 45.455 | 341 | 179 | 5 | 8 | 345 | 4 | 340 | 2.05E-113 | 332 |
| Q6WUC1.1 | EVM0014567.1 | Papaver somniferum | 6OMT | 46.11 | 347 | 172 | 10 | 8 | 345 | 15 | 355 | 1.44E-104 | 310 |
| Q6WUC1.1 | EVM0012543.1 | Papaver somniferum | 6OMT | 43.228 | 347 | 168 | 10 | 8 | 346 | 15 | 340 | 6.75E-95 | 285 |
| Q6WUC1.1 | EVM0005978.1 | Papaver somniferum | 6OMT | 41.36 | 353 | 187 | 8 | 8 | 345 | 3 | 350 | 2.07E-87 | 266 |
| Q6WUC1.1 | EVM0006633.1 | Papaver somniferum | 6OMT | 42.9 | 331 | 173 | 11 | 8 | 328 | 15 | 339 | 5.47E-86 | 262 |
| Q6WUC1.1 | EVM0004237.1 | Papaver somniferum | 6OMT | 40.793 | 353 | 189 | 9 | 8 | 345 | 3 | 350 | 3.75E-82 | 253 |
| Q7XB08.1 | EVM0002778.1 | Papaver somniferum | CNMT | 59.026 | 349 | 142 | 1 | 3 | 351 | 8 | 355 | 1.49E-157 | 445 |
| Q7XB08.1 | EVM0000927_1 | Papaver somniferum | CNMT | 59.026 | 349 | 143 | 0 | 3 | 351 | 8 | 356 | 9.20E-157 | 443 |
| Q7XB08.1 | EVM0006100.1 | Papaver somniferum | CNMT | 54.046 | 346 | 159 | 0 | 6 | 351 | 8 | 353 | 2.08E-138 | 396 |
| Q7XB08.1 | EVM0003935_1 | Papaver somniferum | CNMT | 53.179 | 346 | 162 | 0 | 6 | 351 | 14 | 359 | 4.92E-137 | 393 |
| Q7XB08.1 | EVM0017526.1 | Papaver somniferum | CNMT | 53.314 | 347 | 161 | 1 | 6 | 351 | 8 | 354 | 5.89E-134 | 385 |
| Q7XB08.1 | EVM0015233.1 | Papaver somniferum | CNMT | 52.45 | 347 | 164 | 1 | 6 | 351 | 8 | 354 | 4.69E-133 | 383 |
| Q7XB08.1 | EVM0014166.1 | Papaver somniferum | CNMT | 51.594 | 345 | 167 | 0 | 7 | 351 | 16 | 360 | 5.27E-130 | 375 |
| Q7XB08.1 | EVM0002645.1 | Papaver somniferum | CNMT | 52.312 | 346 | 165 | 0 | 6 | 351 | 8 | 353 | 3.70E-129 | 373 |
| Q7XB08.1 | EVM0012281.1 | Papaver somniferum | CNMT | 51.312 | 343 | 164 | 2 | 9 | 351 | 17 | 356 | 4.89E-125 | 362 |
| Q7XB08.1 | EVM0005266.1 | Papaver somniferum | CNMT | 49.854 | 343 | 169 | 2 | 9 | 351 | 17 | 356 | 8.86E-119 | 347 |
| Q7XB08.1 | EVM0012664.1 | Papaver somniferum | CNMT | 48.462 | 260 | 100 | 6 | 92 | 351 | 35 | 260 | 5.62E-70 | 218 |
| Q9SP06.1 | EVM0002703.1 | Papaver somniferum | NMCH | 64.358 | 491 | 153 | 5 | 12 | 480 | 18 | 508 | 0 | 624 |
| Q9SP06.1 | EVM0012200.1 | Papaver somniferum | NMCH | 50 | 474 | 231 | 4 | 10 | 480 | 13 | 483 | 2.89E-178 | 508 |
| Q9SP06.1 | EVM0008901.1 | Papaver somniferum | NMCH | 46.567 | 466 | 240 | 6 | 19 | 481 | 24 | 483 | 2.26E-160 | 462 |
| Q9SP06.1 | EVM0001635.1 | Papaver somniferum | NMCH | 45.396 | 467 | 237 | 5 | 21 | 478 | 33 | 490 | 1.18E-149 | 435 |
| Q9SP06.1 | EVM0009341.1 | Papaver somniferum | NMCH | 40.161 | 498 | 279 | 7 | 1 | 481 | 6 | 501 | 1.37E-135 | 400 |
| Q9SP06.1 | EVM0016340.1 | Papaver somniferum | NMCH | 40.606 | 495 | 275 | 8 | 2 | 481 | 7 | 497 | 3.25E-134 | 396 |
| Q9SP06.1 | EVM0001459.1 | Papaver somniferum | NMCH | 40.161 | 498 | 277 | 8 | 1 | 481 | 6 | 499 | 1.37E-133 | 394 |
| Q9SP06.1 | EVM0011934.1 | Papaver somniferum | NMCH | 44.946 | 465 | 239 | 7 | 25 | 480 | 47 | 503 | 3.70E-133 | 394 |
| Q9SP06.1 | EVM0007937.1 | Papaver somniferum | NMCH | 40.461 | 477 | 272 | 6 | 12 | 478 | 24 | 498 | 3.92E-132 | 390 |
| Q7XB10.1 | EVM0008782.1 | Papaver somniferum | 4'OMT2 | 54.81 | 343 | 149 | 3 | 20 | 357 | 29 | 370 | 1.70E-143 | 410 |
| Q7XB10.1 | EVM0008621.1 | Papaver somniferum | 4'OMT2 | 47.059 | 340 | 178 | 1 | 20 | 357 | 11 | 350 | 3.59E-121 | 353 |
| Q7XB10.1 | EVM0011328.1 | Papaver somniferum | 4'OMT2 | 47.076 | 342 | 176 | 3 | 20 | 357 | 11 | 351 | 1.34E-118 | 346 |
| Q7XB10.1 | EVM0014567.1 | Papaver somniferum | 4'OMT2 | 44.321 | 361 | 185 | 7 | 6 | 357 | 2 | 355 | 2.46E-108 | 320 |
| Q7XB10.1 | EVM0014836.1 | Papaver somniferum | 4'OMT2 | 43.824 | 340 | 182 | 5 | 20 | 357 | 8 | 340 | 4.31E-107 | 316 |
| Q7XB10.1 | EVM0012543.1 | Papaver somniferum | 4'OMT2 | 41.595 | 351 | 178 | 6 | 12 | 357 | 11 | 339 | 7.27E-95 | 286 |
| Q7XB10.1 | EVM0006633.1 | Papaver somniferum | 4'OMT2 | 41.908 | 346 | 178 | 8 | 6 | 338 | 2 | 337 | 3.10E-91 | 276 |
| Q7XB10.1 | EVM0005978.1 | Papaver somniferum | 4'OMT2 | 40.634 | 347 | 194 | 6 | 20 | 357 | 7 | 350 | 1.33E-90 | 275 |
| Q7XB10.1 | EVM0004237.1 | Papaver somniferum | 4'OMT2 | 40.346 | 347 | 195 | 6 | 20 | 357 | 7 | 350 | 3.73E-89 | 271 |
| Q7XB10.1 | EVM0016742.1 | Papaver somniferum | 4'OMT2 | 40.616 | 357 | 191 | 9 | 17 | 357 | 12 | 363 | 1.24E-74 | 234 |
| AAC61839.1 | EVM0011203.1 | Papaver somniferum | BBE | 46.729 | 535 | 270 | 8 | 3 | 526 | 4 | 534 | 6.05E-165 | 479 |
| AAC61839.1 | EVM0006678.1 | Papaver somniferum | BBE | 49.813 | 534 | 233 | 9 | 33 | 532 | 52 | 584 | 1.27E-164 | 479 |
| AAC61839.1 | EVM0000976.1 | Papaver somniferum | BBE | 48.444 | 514 | 241 | 10 | 31 | 523 | 28 | 538 | 3.94E-147 | 432 |
| AAC61839.1 | EVM0008977.1 | Papaver somniferum | BBE | 47.654 | 405 | 203 | 4 | 127 | 523 | 1 | 404 | 1.12E-123 | 368 |
| B5UAQ8.1 | EVM0004494.1 | Eschscholzia californica | CFS | 45.902 | 488 | 243 | 8 | 18 | 487 | 24 | 508 | 9.74E-153 | 444 |
| Q50LH4.1 | EVM0004494.1 | Eschscholzia californica | SPS | 45 | 500 | 252 | 8 | 17 | 495 | 12 | 509 | 2.16E-151 | 441 |
| Q108P1.1 | EVM0000927_1 | Papaver somniferum | TNMT | 50.725 | 345 | 169 | 1 | 14 | 358 | 13 | 356 | 3.16E-129 | 373 |
| Q108P1.1 | EVM0002778.1 | Papaver somniferum | TNMT | 49.855 | 345 | 171 | 1 | 14 | 358 | 13 | 355 | 8.7E-129 | 372 |
| Q108P1.1 | EVM0003935_1 | Papaver somniferum | TNMT | 48.696 | 345 | 176 | 1 | 14 | 358 | 16 | 359 | 8.85E-124 | 360 |
| Q108P1.1 | EVM0006100.1 | Papaver somniferum | TNMT | 47.578 | 351 | 183 | 1 | 8 | 358 | 4 | 353 | 4.51E-122 | 355 |
| Q108P1.1 | EVM0015233.1 | Papaver somniferum | TNMT | 46.875 | 352 | 185 | 2 | 8 | 358 | 4 | 354 | 1.68E-119 | 348 |
| Q108P1.1 | EVM0017526.1 | Papaver somniferum | TNMT | 46.591 | 352 | 186 | 2 | 8 | 358 | 4 | 354 | 3.21E-118 | 345 |
| Q108P1.1 | EVM0002645.1 | Papaver somniferum | TNMT | 47.11 | 346 | 182 | 1 | 13 | 358 | 9 | 353 | 1.46E-114 | 336 |
| Q108P1.1 | EVM0014166.1 | Papaver somniferum | TNMT | 45.584 | 351 | 190 | 1 | 8 | 358 | 11 | 360 | 5.27E-114 | 335 |
| Q108P1.1 | EVM0012281.1 | Papaver somniferum | TNMT | 45.481 | 343 | 183 | 2 | 16 | 358 | 18 | 356 | 2.83E-106 | 315 |
| Q108P1.1 | EVM0005266.1 | Papaver somniferum | TNMT | 46.939 | 343 | 178 | 2 | 16 | 358 | 18 | 356 | 1.13E-103 | 308 |
| Q108P1.1 | EVM0012664.1 | Papaver somniferum | TNMT | 40.702 | 285 | 131 | 9 | 76 | 358 | 12 | 260 | 2.98E-56 | 183 |
| L7X0L7.1 | EVM0007338.1 | Papaver somniferum | P6H | 42.495 | 553 | 281 | 10 | 7 | 537 | 3 | 540 | 1.37E-154 | 452 |
| L7X0L7.1 | EVM0006719.1 | Papaver somniferum | P6H | 45.267 | 486 | 254 | 6 | 55 | 532 | 40 | 521 | 1.43E-152 | 446 |
| L7X0L7.1 | EVM0009330.1 | Papaver somniferum | P6H | 42.202 | 545 | 286 | 10 | 7 | 538 | 4 | 532 | 1.56E-152 | 446 |
| L7X0L7.1 | EVM0005670.1 | Papaver somniferum | P6H | 45.062 | 486 | 255 | 6 | 55 | 532 | 40 | 521 | 5.52E-150 | 440 |
| L7X0L7.1 | EVM0002156.1 | Papaver somniferum | P6H | 45.528 | 492 | 257 | 8 | 54 | 537 | 37 | 525 | 1.97E-148 | 436 |
| L7X0L7.1 | EVM0000405.1 | Papaver somniferum | P6H | 43.487 | 522 | 280 | 9 | 22 | 537 | 35 | 547 | 2.07E-148 | 436 |
| L7X0L7.1 | EVM0018114.1 | Papaver somniferum | P6H | 44.19 | 525 | 271 | 12 | 18 | 537 | 7 | 514 | 1.38E-146 | 431 |
| L7X0L7.1 | EVM0015053.1 | Papaver somniferum | P6H | 44 | 500 | 266 | 9 | 22 | 515 | 11 | 502 | 8.3E-146 | 429 |
| L7X0L7.1 | EVM0009551.1 | Papaver somniferum | P6H | 42.505 | 527 | 283 | 11 | 20 | 537 | 16 | 531 | 1.29E-142 | 421 |
| L7X0L7.1 | EVM0017761.1 | Papaver somniferum | P6H | 45.695 | 453 | 235 | 6 | 55 | 501 | 4 | 451 | 1.61E-142 | 418 |
| L7X0L7.1 | EVM0004610.1 | Papaver somniferum | P6H | 42.095 | 525 | 280 | 10 | 20 | 537 | 16 | 523 | 2.47E-142 | 420 |
| L7X0L7.1 | EVM0007794.1 | Papaver somniferum | P6H | 41.935 | 527 | 279 | 10 | 21 | 538 | 13 | 521 | 1.12E-141 | 418 |
| L7X0L7.1 | EVM0009476.1 | Papaver somniferum | P6H | 40.805 | 522 | 292 | 7 | 20 | 537 | 7 | 515 | 1.73E-141 | 417 |
| L7X0L7.1 | EVM0005523.1 | Papaver somniferum | P6H | 43.086 | 499 | 259 | 7 | 45 | 537 | 35 | 514 | 1.1E-140 | 416 |
| L7X0L7.1 | EVM0000133.1 | Papaver somniferum | P6H | 44.266 | 497 | 245 | 9 | 48 | 537 | 32 | 503 | 1.96E-140 | 414 |
| L7X0L7.1 | EVM0017491.1 | Papaver somniferum | P6H | 41.746 | 527 | 283 | 9 | 21 | 538 | 13 | 524 | 1.19E-139 | 413 |
| L7X0L7.1 | EVM0005930.1 | Papaver somniferum | P6H | 45.183 | 436 | 224 | 7 | 58 | 482 | 44 | 475 | 1.65E-139 | 411 |
| L7X0L7.1 | EVM0016799.1 | Papaver somniferum | P6H | 41.758 | 546 | 295 | 12 | 1 | 532 | 1 | 537 | 2.39E-139 | 413 |
| L7X0L7.1 | EVM0003486.1 | Papaver somniferum | P6H | 44.534 | 494 | 259 | 8 | 55 | 538 | 40 | 528 | 3.73E-139 | 412 |
| L7X0L7.1 | EVM0017004.1 | Papaver somniferum | P6H | 42.254 | 497 | 261 | 9 | 46 | 537 | 27 | 502 | 2.41E-138 | 409 |
| L7X0L7.1 | EVM0004589.1 | Papaver somniferum | P6H | 43.408 | 493 | 266 | 6 | 55 | 538 | 40 | 528 | 4.66E-137 | 407 |
| L7X0L7.1 | EVM0012792.1 | Papaver somniferum | P6H | 41.4 | 500 | 257 | 9 | 7 | 484 | 3 | 488 | 1.05E-134 | 399 |
| L7X0L7.1 | EVM0000340.1 | Papaver somniferum | P6H | 41.551 | 503 | 265 | 11 | 40 | 538 | 21 | 498 | 2.38E-128 | 383 |
| L7X0L7.1 | EVM0013378.1 | Papaver somniferum | P6H | 41.017 | 295 | 128 | 10 | 269 | 538 | 2 | 275 | 2.54E-62 | 207 |
| L7X3S1.1 | EVM0006719.1 | Papaver somniferum | MSH | 48.77 | 488 | 237 | 8 | 45 | 524 | 39 | 521 | 7.91E-165 | 477 |
| L7X3S1.1 | EVM0005670.1 | Papaver somniferum | MSH | 49.18 | 488 | 235 | 8 | 45 | 524 | 39 | 521 | 1.1E-163 | 474 |
| L7X3S1.1 | EVM0009330.1 | Papaver somniferum | MSH | 46.226 | 530 | 264 | 10 | 12 | 524 | 1 | 526 | 2.39E-162 | 471 |
| L7X3S1.1 | EVM0003486.1 | Papaver somniferum | MSH | 48.697 | 499 | 240 | 9 | 36 | 524 | 30 | 522 | 9.31E-158 | 459 |
| L7X3S1.1 | EVM0002156.1 | Papaver somniferum | MSH | 47.106 | 501 | 247 | 11 | 34 | 524 | 28 | 520 | 4.22E-155 | 452 |
| L7X3S1.1 | EVM0018114.1 | Papaver somniferum | MSH | 47.269 | 531 | 240 | 16 | 12 | 524 | 1 | 509 | 6.93E-154 | 449 |
| L7X3S1.1 | EVM0004589.1 | Papaver somniferum | MSH | 47.495 | 499 | 246 | 8 | 36 | 524 | 30 | 522 | 2.29E-153 | 447 |
| L7X3S1.1 | EVM0007338.1 | Papaver somniferum | MSH | 44.867 | 526 | 260 | 11 | 22 | 524 | 17 | 535 | 6.41E-152 | 444 |
| L7X3S1.1 | EVM0000405.1 | Papaver somniferum | MSH | 44.618 | 511 | 268 | 7 | 24 | 524 | 37 | 542 | 1.82E-149 | 438 |
| L7X3S1.1 | EVM0009551.1 | Papaver somniferum | MSH | 45.233 | 493 | 252 | 8 | 39 | 524 | 45 | 526 | 2.2E-149 | 437 |
| L7X3S1.1 | EVM0015053.1 | Papaver somniferum | MSH | 45.935 | 492 | 254 | 8 | 34 | 521 | 30 | 513 | 4.66E-149 | 437 |
| L7X3S1.1 | EVM0009476.1 | Papaver somniferum | MSH | 44.401 | 509 | 268 | 10 | 22 | 524 | 11 | 510 | 8.1E-148 | 433 |
| L7X3S1.1 | EVM0004610.1 | Papaver somniferum | MSH | 46.694 | 484 | 241 | 10 | 44 | 524 | 49 | 518 | 2.06E-147 | 432 |
| L7X3S1.1 | EVM0017761.1 | Papaver somniferum | MSH | 46.799 | 453 | 233 | 5 | 45 | 493 | 3 | 451 | 2E-145 | 425 |
| L7X3S1.1 | EVM0007794.1 | Papaver somniferum | MSH | 43.13 | 524 | 270 | 11 | 14 | 524 | 7 | 515 | 2.51E-145 | 427 |
| L7X3S1.1 | EVM0005523.1 | Papaver somniferum | MSH | 44.311 | 501 | 250 | 10 | 33 | 524 | 29 | 509 | 5.56E-145 | 426 |
| L7X3S1.1 | EVM0017004.1 | Papaver somniferum | MSH | 42.829 | 509 | 263 | 9 | 22 | 524 | 11 | 497 | 2.52E-141 | 416 |
| L7X3S1.1 | EVM0000340.1 | Papaver somniferum | MSH | 43.57 | 521 | 257 | 12 | 12 | 524 | 1 | 492 | 5.15E-138 | 407 |
| L7X3S1.1 | EVM0017491.1 | Papaver somniferum | MSH | 43.21 | 486 | 258 | 9 | 14 | 489 | 7 | 484 | 8.81E-138 | 408 |
| L7X3S1.1 | EVM0012792.1 | Papaver somniferum | MSH | 44.581 | 489 | 234 | 12 | 12 | 470 | 1 | 482 | 9.02E-137 | 404 |
| L7X3S1.1 | EVM0000133.1 | Papaver somniferum | MSH | 43.287 | 499 | 252 | 10 | 33 | 524 | 24 | 498 | 1.05E-136 | 404 |
| L7X3S1.1 | EVM0005930.1 | Papaver somniferum | MSH | 46.014 | 439 | 221 | 9 | 47 | 474 | 42 | 475 | 2.98E-136 | 402 |
| L7X3S1.1 | EVM0016799.1 | Papaver somniferum | MSH | 42.884 | 534 | 277 | 14 | 14 | 524 | 9 | 537 | 4.05E-132 | 394 |
| L7X3S1.1 | EVM0008748.1 | Papaver somniferum | MSH | 40.974 | 493 | 260 | 10 | 54 | 524 | 68 | 551 | 8.18E-127 | 381 |
| L7X3S1.1 | EVM0007461.1 | Papaver somniferum | MSH | 41.752 | 491 | 265 | 11 | 46 | 524 | 52 | 533 | 9.33E-118 | 357 |
| AGL44334.1 | EVM0016944.1 | Papaver somniferum | DBOX | 47.843 | 510 | 244 | 11 | 35 | 530 | 38 | 539 | 5.04E-158 | 461 |
| AGL44334.1 | EVM0006867.1 | Papaver somniferum | DBOX | 44.776 | 536 | 285 | 7 | 3 | 531 | 1 | 532 | 1.95E-155 | 454 |
| AGL44334.1 | EVM0010203.1 | Papaver somniferum | DBOX | 44.61 | 538 | 288 | 7 | 1 | 531 | 1 | 535 | 4.81E-155 | 453 |
| AGL44334.1 | EVM0016772.1 | Papaver somniferum | DBOX | 46.337 | 505 | 259 | 6 | 35 | 531 | 39 | 539 | 3.62E-154 | 451 |
| AGL44334.1 | EVM0013897.1 | Papaver somniferum | DBOX | 44.424 | 538 | 286 | 9 | 3 | 531 | 1 | 534 | 1.71E-149 | 439 |
| AGL44334.1 | EVM0004268.1 | Papaver somniferum | DBOX | 47.755 | 490 | 235 | 9 | 51 | 531 | 37 | 514 | 3.53E-146 | 431 |
| AGL44334.1 | EVM0004314.1 | Papaver somniferum | DBOX | 44.052 | 538 | 280 | 9 | 2 | 531 | 34 | 558 | 3.67E-146 | 431 |
| AGL44334.1 | EVM0009143.1 | Papaver somniferum | DBOX | 44.353 | 487 | 217 | 10 | 51 | 531 | 55 | 493 | 1.35E-122 | 370 |
| AGL44334.1 | EVM0008977.1 | Papaver somniferum | DBOX | 40.196 | 408 | 233 | 6 | 133 | 532 | 1 | 405 | 1.9E-92 | 288 |
| A0A1C9U5X5.1 | EVM0000927_1 | Papaver somniferum | RNMT | 50.852 | 352 | 167 | 4 | 10 | 358 | 8 | 356 | 2.74E-133 | 384 |
| A0A1C9U5X5.1 | EVM0003935_2 | Papaver somniferum | RNMT | 51.944 | 360 | 167 | 4 | 2 | 358 | 4 | 360 | 1.39E-132 | 382 |
| A0A1C9U5X5.1 | EVM0002778.1 | Papaver somniferum | RNMT | 49.716 | 352 | 170 | 4 | 10 | 358 | 8 | 355 | 1.37E-130 | 377 |
| A0A1C9U5X5.1 | EVM0014166.1 | Papaver somniferum | RNMT | 50.833 | 360 | 171 | 4 | 2 | 358 | 4 | 360 | 1.06E-125 | 365 |
| A0A1C9U5X5.1 | EVM0006100.1 | Papaver somniferum | RNMT | 51.009 | 347 | 164 | 4 | 15 | 358 | 10 | 353 | 5.32E-125 | 363 |
| A0A1C9U5X5.1 | EVM0015233.1 | Papaver somniferum | RNMT | 51.149 | 348 | 163 | 5 | 15 | 358 | 10 | 354 | 2.98E-124 | 361 |
| A0A1C9U5X5.1 | EVM0017526.1 | Papaver somniferum | RNMT | 50.287 | 348 | 166 | 5 | 15 | 358 | 10 | 354 | 2.30E-121 | 353 |
| A0A1C9U5X5.1 | EVM0002645.1 | Papaver somniferum | RNMT | 46.991 | 349 | 179 | 4 | 13 | 358 | 8 | 353 | 1.19E-112 | 332 |
| A0A1C9U5X5.1 | EVM0012281.1 | Papaver somniferum | RNMT | 43.109 | 341 | 187 | 4 | 20 | 358 | 21 | 356 | 1.83E-103 | 308 |
| A0A1C9U5X5.1 | EVM0005266.1 | Papaver somniferum | RNMT | 44.152 | 342 | 182 | 5 | 20 | 358 | 21 | 356 | 1.77E-101 | 303 |
| A0A1C9U5X5.1 | EVM0012664.1 | Papaver somniferum | RNMT | 40.293 | 273 | 124 | 7 | 90 | 358 | 23 | 260 | 2.04E-57 | 187 |
| I3V6A7 | EVM0000250.1 | Papaver somniferum | SOMT | 40.62 | 352 | 170 | 9 | 14 | 329 | 42 | 390 | 1.00E-76 | 233 |
| I3V6A7 | EVM0002781.1 | Papaver somniferum | SOMT | 42.08 | 366 | 188 | 7 | 2 | 346 | 28 | 390 | 5.00E-97 | 286 |
| 3V6A7 | EVM0013337.1 | Papaver somniferum | SOMT | 43.44 | 366 | 195 | 6 | 9 | 362 | 25 | 390 | 7.00E-101 | 297 |
| Q948Y1 | EVM0004494.1 | Coptis japonica | CAS | 50.63 | 478 | 216 | 8 | 35 | 510 | 32 | 491 | 2.00E-163 | 466 |
| BAJ40864.1 | EVM0009143.1 | Coptis japonica | STOX | 43.84 | 536 | 238 | 8 | 14 | 495 | 8 | 534 | 2.00E-133 | 392 |
| BAJ40864.1 | EVM0016772.1 | Coptis japonica | STOX | 47.37 | 513 | 255 | 8 | 33 | 539 | 29 | 532 | 3.00E-161 | 464 |
| BAJ40864.1 | EVM0016944.1 | Coptis japonica | STOX | 47.44 | 546 | 264 | 9 | 1 | 539 | 2 | 531 | 8.00E-168 | 481 |
| BAJ40864.1 | EVM0010203.1 | Coptis japonica | STOX | 47.64 | 508 | 253 | 8 | 33 | 535 | 33 | 532 | 4.00E-146 | 425 |
| BAJ40864.1 | EVM0004314.1 | Coptis japonica | STOX | 48.43 | 508 | 251 | 7 | 58 | 560 | 33 | 534 | 5.00E-155 | 449 |
| BAJ40864.1 | EVM0013897.1 | Coptis japonica | STOX | 48.62 | 508 | 250 | 7 | 34 | 536 | 33 | 534 | 2.00E-157 | 454 |
| BAJ40864.1 | EVM0006867.1 | Coptis japonica | STOX | 48.62 | 506 | 248 | 7 | 33 | 532 | 33 | 532 | 1.00E-155 | 449 |
| BAJ40864.1 | EVM0004268.1 | Coptis japonica | STOX | 48.66 | 524 | 249 | 7 | 3 | 516 | 21 | 534 | 2.00E-161 | 465 |

Table S11. GenBank accession numbers of complete chloroplast genomes of 36 species used for phylogenetic analysis.

| **Species** | **GenBank acc.** |
| --- | --- |
| *Ginkgo biloba* | MN443423.1 |
| *Amborella trichopoda* | NC_005086 |
| *Nymphaea colorata* | NC_057562.1 |
| *Chloranthus spicatus* | NC_009598 |
| *Aristolochia contorta* | NC_036152.1 |
| *Aristolochia fimbriata* | CM034085.1 |
| *Piper nigrum* | NC_034692 |
| *Piper kadsura* | NC_027941 |
| *Litsea cubeba* | NC_048954.1 |
| *Phoebe sheareri* | KX437773.1 |
| *Cinnamomum micranthum* | NC_035802.1 |
| *Magnolia grandiflora* | NC_020318.1 |
| *Liriodendron chinense* | NC_030504.1 |
| *Lemna minor* | NC_010109.1 |
| *Oryza sativa* | NC_031333.1 |
| *Lilium brownii* | NC_035588.1 |
| *Dendrobium officinale* | NC_024019.1 |
| *Ceratophyllum demersum* | NC_009962.1 |
| *Papaver somniferum* | NC_029434.1 |
| *Macleaya cordata* | MT178411.1 |
| *Trollius chinensis* | NC_031849 |
| *Nelumbo nucifera* | JQ336993.1 |
| *Buxus microphylla* | NC_009599.1 |
| *Vitis vinifera* | NC_007957.1 |
| *Glycine soja* | NC_022868 |
| *Fragaria vesca* | NC_015206.1 |
| *Citrus reticulata* | NC_034671.1 |
| *Arabidopsis thaliana* | NC_000932 |
| *Theobroma cacao* | NC_014676.2 |
| *Cornus chinensis* | NC_044815.1 |
| *Coffea canephora* | NC_030053.1 |
| *Mentha canadensis* | MN102358.1 |
| *Nicotiana tabacum* | NC_001879 |
| *Helianthus annuus* | NC_007977.1 |
| *Daucus carota* | NC_008325.1 |
| *Lonicera japonica* | NC_026839.1 |

| Table S12. P﻿rimers used for cloning AcOMTs | |
| --- | --- |
| ﻿Gene | Primer |
| AcOMT1 -F | GGATCCATGGAAACACCGAAAGGCG |
| AcOMT1-R | GCGGCCGCTTAGTAAGGAAATGCCTCAATCACAGA |
| AcOMT2 -F | GGATCCATGGAGACACCGAAAGACGATCA |
| AcOMT2-R | GCGGCCGCTTAGTAAGGAAATGCCTCAATCACAGA |
| AcOMT3 -F | GGATCCATGAAGATGGATATCATGAACCTGC |
| AcOMT3-R | GCGGCCGCATAGGGGAAGGCCTCGATCACAGATTG |
| AcOMT5 -F | GGATCCATGGCCGATCCAGTTGCAG |
| AcOMT5 -R | GCGGCCGCAGGATAAGCTTCAATTACAG |
| AcOMT7-F | GAGCTCATGGAGGCGGAAAAGGACGTTC |
| AcOMT7-R | GCGGCCGCAGGGAAGAGTTCGATAATTGATTCG |

| Table S13. Primer sequences used for qPCR | | |  |
| --- | --- | --- | --- |
| Gene ID | Gene Description | Forward Primer sequences (5'-3′) | Reverse Primer sequences (5'-3′) |
| EVM0012696 | Actin | TTCAATGTCCCAGCCATGTACGTT | ACCGGAATCCAGTACAATACCAG |
| EVM0008901 | CYP80G | CCTAGATAGCGCTGAAGTC | CGGAGAAGAAGGGTTTGAGC |
| EVM0002703 | CYP80B | CCTGCTTATGGAAATGTTCGG | GTCTCTGAGCCTTGACGTG |
| EVM0012200 | CYP80 | CAGCTACCTTCACCAATCC | CTTGGGAGAAGATGGGG |
| EVM0001635 | CYP80 | CAGTGGAGTTTAGCATACGG | CGTCTCTTGGTCGATAAAGTC |
| EVM0008024 | CYP81 | CAACCCATTGGAGGCAG | GCAGTACTGAAGCTCGCTC |
| EVM0004494 | CYP719 | GGAGAATATTGGTAAACCAC | CCAAATGGAAGGAAGGATC |
| EVM0011328 | OMT | GGAAGCTGTGCCCATAAAAGG | GGGTTTGGGGACGATCA |
| EVM0008782 | OMT | GCAGATGAGGAAAGGAGAGG | GCCGAAGATGTGTTTCCAAAG |
| EVM0014836 | OMT | GCTAAAGACCCCAAAGCTAACCAG | CATTAAGGATCCCGTCGCTCT |
| EVM0014567 | OMT | TATGGAAACTCACCCACGCCTTC | CGCCGTGGGAATGGATAATA |
| EVM0016813 | OMT | GCCATGCCCATTCTCGAT | TCCTTGTCCGCGTCCAC |
| EVM0011362 | OMT | GCTCTTCCATACCTCGACCAC | TAGTTCGTCTTGTCAGCGTCT |
| EVM0013337 | OMT | AGGCACCAATTTCGATCTGCAA | CTCAGCACTTGGAACTTTGACA |
| EVM0006633 | OMT | GATCAGTTGCACCGTCTTCG | GTCTCCTTTAACTCGAGCGAT |
| EVM0002543 | OMT | CTTGGTGCTGGTTATTCCGGGTA | CGCCATCCACATTGAAGGGT |
