# Supplementary material for: Chromosome-level genome assembly of Aristolochia contorta provides insights into the biosynthesis of benzylisoquinoline alkaloids and aristolochic acids
Source: Hortic Res. 2022 Feb 11;9:uhac005. doi: 10.1093/hr/uhac005 (PMC8973263; doi:10.1093/hr/uhac005)
Supplement: Web_Material_uhac005 [file web_material_uhac005.zip › TableS1-13.pdf]

Table S1. Hi-C Assembly data statistics

Group	Cluster Num	Cluster Len(bp)	Order Num	Order Len(bp)
LG01	18	32,422,454	11	31,427,636
LG02	42	39,786,356	31	38,740,436
LG03	55	33,945,346	38	31,589,686
LG04	29	31,661,893	22	30,379,727
LG05	26	28,091,832	17	26,144,098
LG06	17	19,748,185	14	19,487,744
LG07	27	23,609,743	14	20,791,271
Total(Ratio %)	214(95.54)	209265809(99.4)	147(68.69)	198560598(94.88)

Table S2. Post-Hi-C genome statistics

Parameter	Number/Length
Scaffold number	84
Scaffold length (bp)	210,540,769
Scaffold N50 (bp)	30,381,827
Scaffold N90 (bp)	19,489,044
Scaffold max (bp)	38,743,436
Gap total length (bp)	14,000
Contig number	224
Contig length (bp)	210,526,769
Contig N50 (bp)	2,315,928
Contig N90 (bp)	388,540
Contig max (bp)	12,320,100
GC content (%)	39.39

Table S3. Validation of genome assembly using BUSCO and CEGMA method

Species	Complete BUSCOs	Complete and single-copy BUSCOs	Complete and duplicated BUSCOs	Fragmented BUSCOs	Missing BUSCOs
A. contorta	1,300 (90.28%)	1,233 (85.62%)	67 (4.65%)	41 (2.85%)	99 (6.88%)

Species	Number of 458 CEGs* present in assembly	% of 458 CEGs present in assemblies	Number of 248 highly conserved CEGs present	% of 248 highly conserved CEGs present
A. contorta	446	97.38%	224	90.32%

Table S4. Repeat sequence statistics

Type	**Number**	**Length**	**Rate(%)**
ClassI	130,737	58,442,626	27.76
ClassI/DIRS	8,025	4,477,176	2.13
ClassI/LINE	19,091	10,292,880	4.89
ClassI/LTR	392	190,944	0.09
ClassI/LTR/Copia	44,815	21,996,014	10.45
ClassI/LTR/Gypsy	18,389	10,867,633	5.16
ClassI/PLE|LARD	35,272	13,382,265	6.36
ClassI/SINE	2,453	420,386	0.2
ClassI/SINE|TRIM	38	6,621	0
ClassI/TRIM	1,030	290,407	0.14
ClassI/Unknown	1,232	336,302	0.16
ClassII	25,054	9,546,083	4.53
ClassII/Crypton	1	49	0
ClassII/Helitron	399	32,036	0.02
ClassII/MITE	1,292	201,801	0.1
ClassII/Maverick	2,045	550,014	0.26
ClassII/TIR	19,832	8,698,883	4.13
ClassII/Unknown	1,485	131,631	0.06
PotentialHostGene	8,446	2,394,407	1.14
Total	165,811	80,555,983	38.26

Table S5. Statistical of gene prediction

**Method**	**Software**	**Species**	**Gene number**
Ab initio	Genscan	-	19,156
	Augustus	-	13,113
	GlimmerHMM	-	22,245
	GeneID	-	30,495
	SNAP	-	25,902
Homology-based	GeMoMa	Arabidopsis_thaliana	16,226
		Oryza sativa Japonica	15,903
		Cinnamomum kanehirae	16,642
		Olea europaea	19,790
RNAseq	PASA	-	8,839
Integration	EVM	-	18,311

Table S6. Statistical of genetic information

Parameter	Value
Gene Num	18,311
GeneLen (bp)	80,906,219
AveGeneLen (bp)	4,418
ExonLen (bp)	27,945,818
AveExonLen(bp)	1526
IntronLen (bp)	52,960,401
AveIntronLen(bp)	2,892

Table S7. Founctional analysis

Parameter	Number	Percentage (%)
Total repetitive sequences	165,811	38.26
Protein-coding genes	18,311	/
rRNA	404	/
tRNA	475	/
miRNA	46	/
Pseudogenes	770	/
All functional annotations	17,765	97.02%
GO_annotations	10,156	55.46%
KEGG_annotations	6,703	36.61%
KOG_annotations	10,553	57.63%
TrEMBL_annotations	17,732	96.84%
Nr_annotations	17,749	96.93%
Unannotated	546	2.98%

Table S8. Characteristics of full-length CYP450s of *A. contorta* identified.

Gene ID	Type	CYP Clan	CYP Family	CYP Subfamily
EVM0014314	A	71	CYP71	CYP71A
EVM0016207	A	71	CYP71	CYP71D
EVM0009965	A	71	CYP71	CYP71A
EVM0014133	A	71	CYP71	
EVM0001806	A	71	CYP71	CYP71D
EVM0004015	A	71	CYP71	
EVM0011884	A	71	CYP71	CYP71D
EVM0011970	A	71	CYP71	CYP71D
EVM0016622	A	71	CYP71	CYP71A
EVM0009150	A	71	CYP71	CYP71A
EVM0004982	A	71	CYP71	CYP71D
EVM0000777	A	71	CYP71	
EVM0013011	A	71	CYP71	CYP71D
EVM0004726	A	71	CYP71	
EVM0003623	A	71	CYP71	
EVM0006325	A	71	CYP71	CYP71D
EVM0006200	A	71	CYP71	CYP71D
EVM0000692	A	71	CYP92	
EVM0016570	A	71	CYP71	
EVM0003085	A	71	CYP71	
EVM0005024	A	71	CYP71	
EVM0002075_1	A	71	CYP93	CYP93A
EVM0002075_2	A	71	CYP93	CYP93A
EVM0008943	A	71	CYP71	CYP71A
EVM0016545	A	71	CYP71	CYP71D
EVM0007823	A	71	CYP71	
EVM0017651	A	71	CYP736	CYP736A
EVM0010960	A	71	CYP76	
EVM0009341	A	71	CYP76	
EVM0010458	A	71	CYP71	CYP71D
EVM0005055	A	71	CYP71	
EVM0006204	A	71	CYP93	CYP93A
EVM0001459	A	71	CYP76	CYP76F
EVM0007937	A	71	CYP76	CYP76F
EVM0001519	A	71	CYP706	
EVM0009949	A	71	CYP84	CYP84A
EVM0007442	A	71	CYP84	CYP84A
EVM0000033	A	71	CYP76	CYP76F

EVM0018211	A	71	CYP76	
EVM0017918	A	71	CYP76	
EVM0017445	A	71	CYP71	
EVM0016340	A	71	CYP76	
EVM0004815	A	71	CYP76	
EVM0008552	A	71	CYP76	
EVM0008759	A	71	CYP706	
EVM0015272	A	71	CYP706	
EVM0004747	A	71	CYP706	
EVM0007827	A	71	CYP706	
EVM0000865	A	71	CYP703	CYP703A
EVM0002446	A	71	CYP706	
EVM0008424	A	71	CYP98	CYP98A
EVM0008104_1	A	71	CYP81	CYP81Q
EVM0008104_2	A	71	CYP81	CYP81Q
EVM0018306	A	71	CYP706	
EVM0005076	A	71	CYP706	
EVM0006218	A	71	CYP706	
EVM0009330	A	71	CYP82	CYP82C
EVM0001383	A	71	CYP93	
EVM0012200	A	71	CYP80	
EVM0018114	A	71	CYP82	CYP82C
EVM0006719	A	71	CYP82	CYP82C
EVM0004889	A	71	CYP706	
EVM0015053	A	71	CYP82	CYP82C
EVM0005067	A	71	CYP706	
EVM0010280	A	71	CYP78	CYP78A
EVM0014042	A	71	CYP706	
EVM0004610	A	71	CYP82	
EVM0001111	A	71	CYP82	
EVM0016112	A	71	CYP75	CYP75B
EVM0001440	A	71	CYP706	CYP706
EVM0009551	A	71	CYP82	CYP82C
EVM0005670	A	71	CYP82	CYP82C
EVM0018090	A	71	CYP71	
EVM0000405	A	71	CYP82	CYP82C
EVM0006780	A	71	CYP706	
EVM0013435	A	71	CYP706	
EVM0012438	A	71	CYP82	
EVM0003486	A	71	CYP82	CYP82C
EVM0004485	A	71	CYP82	
EVM0000695	A	71	CYP71	
EVM0006589	A	71	CYP71	
EVM0009476	A	71	CYP82	

EVM0017491	A	71	CYP82	
EVM0001635	A	71	CYP80	
EVM0007338	A	71	CYP82	
EVM0007794	A	71	CYP82	
EVM0004589	A	71	CYP82	CYP82C
EVM0006707	A	71	CYP706	
EVM0009697	A	71	CYP98	CYP98A
EVM0013783	A	71	CYP706	
EVM0008748	A	71	CYP82	
EVM0000886	A	71	CYP82	
EVM0017761	A	71	CYP82	CYP82C
EVM0013344	A	71	CYP706	
EVM0008901	A	71	CYP80	CYP80G
EVM0017004	A	71	CYP82	
EVM0002629	A	71	CYP81	
EVM0011795	A	71	CYP706	
EVM0002156	A	71	CYP82	CYP82C
EVM0002703	A	71	CYP80	CYP80B
EVM0009777	A	71	CYP706	
EVM0008024	A	71	CYP81	
EVM0005930	A	71	CYP82	CYP82C
EVM0000505	A	71	CYP706	
EVM0000340	A	71	CYP82	CYP82C
EVM0001287	A	71	CYP78	CYP78A
EVM0014504	A	71	CYP78	CYP78A
EVM0012792	A	71	CYP82	
EVM0010384	A	71	CYP706	
EVM0010174	A	71	CYP706	
EVM0016799	A	71	CYP82	CYP82C
EVM0000480	A	71	CYP706	
EVM0012519	A	71	CYP82	
EVM0002746	A	71	CYP706	
EVM0011802	A	71	CYP82	
EVM0002489	A	71	CYP78	CYP78A
EVM0009511	A	71	CYP73	CYP73A
EVM0005040	A	71	CYP706	
EVM0002723	A	71	CYP71	
EVM0007706	A	71	CYP706	
EVM0005523	A	71	CYP82	
EVM0011934	A	71	CYP80	
EVM0017664	A	71	CYP89	CYP89A
EVM0013208	A	71	CYP706	
EVM0001794	A	71	CYP706	
EVM0003110_1	A	71	CYP89	CYP89A

EVM0003110_2	A	71	CYP89	
EVM0003110_3	A	71	CYP89	CYP89A
EVM0013609	A	71	CYP79	CYP79A
EVM0000133	A	71	CYP82	
EVM0013068	A	71	CYP706	
EVM0007461	A	71	CYP82	CYP82C
EVM0004494	A	71	CYP719	
EVM0017199	A	71	CYP77	CYP77B
EVM0011707	A	71	CYP706	
EVM0005435	A	71	CYP706	
EVM0010452	A	71	CYP79	CYP79A
EVM0016237	A	71	CYP79	CYP79A
EVM0011985	A	71	CYP79	CYP79A
EVM0011393	A	71	CYP77	CYP77A
EVM0008616	A	71	CYP701	CYP701A
EVM0005442	A	71	CYP79	CYP79A
EVM0002942	A	71	CYP79	CYP79A
EVM0008747	A	71	CYP79	CYP79A
EVM0006852	A	71	CYP706	
EVM0012874	A	71	CYP79	CYP79A
EVM0015787	A	71	CYP706	
EVM0011719	A	71	CYP76	
EVM0011218	A	71	CYP79	
EVM0001119	A	71	CYP706	
EVM0010225	A	71	CYP89	
EVM0015757	A	71	CYP82	
EVM0000266	A	71	CYP706	
EVM0006725	A	71	CYP706	
EVM0007439	A	71	CYP706	
EVM0016865_1	non-A	72	CYP72	CYP72A
EVM0016865_2	non-A	72	CYP72	CYP72A
EVM0017387	non-A	72	CYP72	CYP72A
EVM0016899	non-A	72	CYP72	CYP72A
EVM0008717	non-A	72	CYP72	CYP72A
EVM0014026	non-A	72	CYP72	CYP72A
EVM0004920	non-A	72	CYP72	CYP72A
EVM0007454	non-A	72	CYP72	CYP72A
EVM0016830	non-A	72	CYP72	CYP72A
EVM0012251	non-A	72	CYP72	CYP72A
EVM0012555	non-A	72	CYP734	CYP734A
EVM0011493	non-A	72	CYP72	CYP72A
EVM0007334	non-A	72	CYP721	CYP721A
EVM0011281	non-A	72	CYP721	CYP721A
EVM0004562	non-A	72	CYP735	CYP735A

EVM0002491	non-A	72	CYP709	CYP709B
EVM0003857	non-A	72	CYP709	CYP709B
EVM0005381	non-A	72	CYP709	CYP709B
EVM0003097	non-A	72	CYP715	
EVM0017376	non-A	72	CYP714	
EVM0001498_1	non-A	72	CYP714	
EVM0015687	non-A	72	CYP714	CYP714C
EVM0001498_2	non-A	72	CYP714	
EVM0004011	non-A	72	CYP714	
EVM0010168	non-A	72	CYP714	
EVM0014128	non-A	72	CYP714	
EVM0002410	non-A	72	CYP714	
EVM0008936	non-A	74	CYP74	CYP74A
EVM0008472	non-A	74	CYP74	CYP74B
EVM0011703	non-A	85	CYP85	CYP85A
EVM0006238	non-A	85	CYP85	CYP85A
EVM0009290	non-A	85	CYP90	CYP90D
EVM0005573	non-A	85	CYP87	CYP87A
EVM0002089	non-A	85	CYP90	CYP90A
EVM0013807	non-A	85	CYP90	CYP90B
EVM0007709	non-A	85	CYP87	CYP87A
EVM0010503	non-A	85	CYP87	CYP87A
EVM0010777	non-A	85	CYP724	CYP724B
EVM0005890	non-A	85	CYP720	CYP720A
EVM0010408	non-A	85	CYP724	CYP724B
EVM0004053	non-A	85	CYP724	CYP724B
EVM0016312	non-A	85	CYP707	CYP707A
EVM0017535	non-A	85	CYP707	CYP707A
EVM0012763	non-A	85	CYP729	
EVM0009910	non-A	85	CYP707	CYP707A
EVM0005736	non-A	85	CYP716	CYP716B
EVM0008283	non-A	85	CYP90	CYP90C
EVM0005688	non-A	85	CYP88	CYP88A
EVM0001010	non-A	85	CYP90	
EVM0010938	non-A	85	CYP733	
EVM0005940	non-A	85	CYP716	CYP716B
EVM0016151	non-A	85	CYP722	
EVM0002396	non-A	85	CYP722	
EVM0010801	non-A	86	CYP86	CYP86A
EVM0014530	non-A	86	CYP86	CYP86A
EVM0004350	non-A	86	CYP86	CYP86B
EVM0017114	non-A	86	CYP86	CYP86C
EVM0016761	non-A	86	CYP94	CYP94A
EVM0017500	non-A	86	CYP94	CYP94B

EVM0010671_1	non-A	86	CYP94	CYP94A
EVM0010671_2	non-A	86	CYP94	CYP94A
EVM0009397	non-A	86	CYP94	CYP94B
EVM0017164	non-A	86	CYP94	CYP94B
EVM0004697	non-A	86	CYP94	CYP94B
EVM0002442	non-A	86	CYP94	CYP94B
EVM0011679	non-A	86	CYP94	CYP94B
EVM0017670	non-A	86	CYP94	CYP94C
EVM0007829_1	non-A	86	CYP96	
EVM0001329	non-A	86	CYP96	
EVM0001396	non-A	86	CYP96	
EVM0004731	non-A	86	CYP96	
EVM0008867	non-A	86	CYP704	CYP704A
EVM0011064	non-A	86	CYP96	
EVM0001231	non-A	86	CYP96	
EVM0007829_2	non-A	86	CYP96	
EVM0007996	non-A	86	CYP96	
EVM0005263	non-A	86	CYP704	CYP704A
EVM0017105	non-A	86	CYP96	
EVM0009878	non-A	86	CYP704	CYP704B
EVM0003066	non-A	97	CYP97	CYP97A
EVM0012894	non-A	97	CYP97	CYP97C
EVM0007861	non-A	97	CYP97	CYP97B
EVM0014428	non-A	710	CYP710	CYP710A
EVM0008671	non-A	711	CYP711	CYP711A
EVM0011753	non-A	727	CYP727	
EVM0010488	non-A	51	CYP51	CYP51G

Table. S9 BIAs and its derivatives in metabolome profiling results

NO.	Metabolites	Formula	Structure
1	Reticuline	C19H23NO4	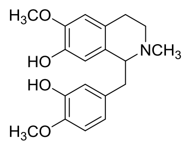
2	Cepharadione A	C18H11NO4	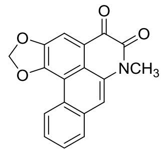
3	*N*-Methylcoclaurine	C18H21NO3	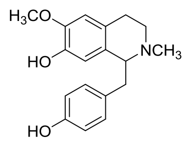
4	Secoisotetrandrine	C38H40N2O8	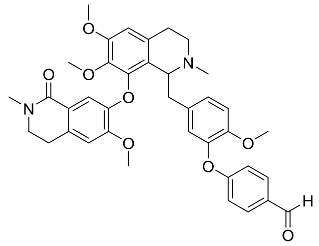
5	Norcorydine	C19H21NO4	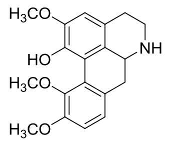
6	Isocorydine	C20H23NO4	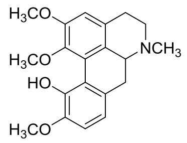
7	*O*-Methylarmepavine	C20H25NO3	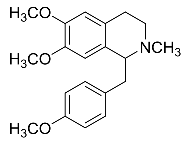
8	Annocherine A	C17H15NO4	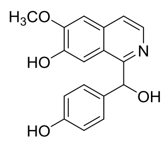
9	Annocherine B	C18H17NO4	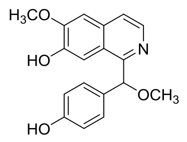
10	*N*-Acetylanonaine	C19H17NO3	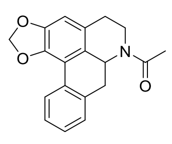

11	Anonaine	C17H15NO2	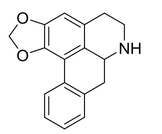
12	Aristolodione	C18H13NO4	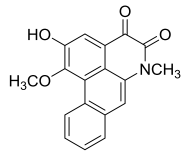
13	Laurelliptine	C18H19NO4	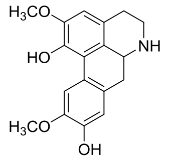
14	6-Acetylmorphine	C19H21NO4	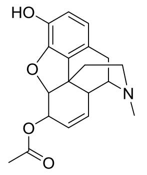
15	Genomorphine	C17H19NO4	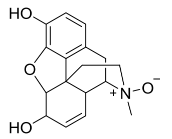
16	Morphine	C17H19NO3	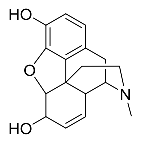
17	Nandigerine	C18H17NO4	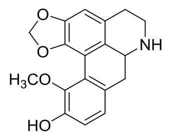
18	Norsanguinarine	C19H11NO4	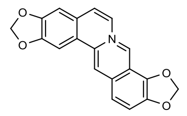
19	Oxonantenine	C19H13NO5	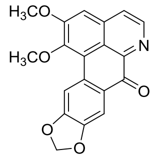
20	Noscapine	C22H23NO7	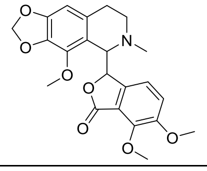
21	Protopine	C20H19NO5	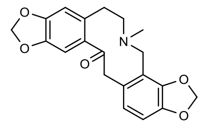

22	Morphinan	C19H21NO3	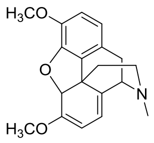
23	Dehydroaporheine	C18H15NO2	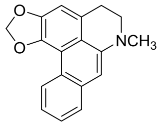
24	3'-Hydroxy-*N*-Methylcoclaurine	C18H21NO4	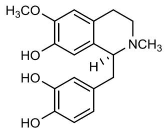

Table S10. 91 predicted nonredundant genes that were probably involved in the biosynthesis of BIAs in *A. contorta*.

query ID	subject ID	species	putative function	identity	alignment length	mismatches	gap openings	q. start	q. end	s. start	s. end	e-value	bit score
O82415.2	EVM0009250.1	Papaver somniferum	TYDC	77.255	510	110	2	1	510	1	504	0	838
O82415.2	EVM0004514.1	Papaver somniferum	TYDC	60.041	493	183	3	19	511	91	569	0	654
O82415.2	EVM0008727.1	Papaver somniferum	TYDC	55.212	518	220	4	1	510	1	514	0	613
ADC33123.1	EVM0014610.1	Papaver somniferum	TyrAT	62.103	409	151	2	1	407	1	407	0	525
OVA00542.1	EVM0006564.1	Macleaya cordata	3OHase	61.663	433	139	6	4	411	1	431	0	532
OVA00542.1	EVM0013678.1	Macleaya cordata	3OHase	66.116	363	116	5	55	411	60	421	1.10E-171	494
OVA00542.1	EVM0017300.1	Macleaya cordata	3OHase	49.068	322	153	7	96	411	82	398	9.91E-106	324
OVA00542.1	EVM0015372.1	Macleaya cordata	3OHase	47.091	361	168	12	66	411	76	428	1.70E-103	313
OVA00542.1	EVM0005329.1	Macleaya cordata	3OHase	45.257	369	176	12	63	411	95	457	6.23E-91	287
OVA02291.1	EVM0004514.1	Macleaya cordata	4HPPDC	77.686	484	108	0	36	519	86	569	0	810
OVA02291.1	EVM0009250.1	Macleaya cordata	4HPPDC	58.98	490	193	1	39	520	17	506	0	633
OVA02291.1	EVM0008727.1	Macleaya	4HPPDC	53.333	495	218	2	39	520	22	516	0	565

		cordata											
Q4QTJ2.1	EVM0001864.1	Papaver somniferum	NCS1	38.71	155	88	4	39	190	1	151	7.33E-30	108
Q4QTJ2.1	EVM0010147.1	Papaver somniferum	NCS1	37.419	155	90	4	39	190	1	151	2.42E-28	104
Q4QTJ2.1	EVM0005050.1	Papaver somniferum	NCS1	37.87	169	93	5	30	193	148	309	8.09E-26	101
Q4QTJ2.1	EVM0014712.1	Papaver somniferum	NCS1	35.714	154	94	3	39	190	1	151	1.87E-25	97.1
Q4QTJ2.1	EVM0011227.1	Papaver somniferum	NCS1	35.065	154	95	3	39	190	1	151	3.26E-24	93.6
Q4QTJ2.1	EVM0011298.1	Papaver somniferum	NCS1	35.2	125	78	3	48	171	50	172	3.72E-22	89.4
Q4QTJ2.1	EVM0000274.1	Papaver somniferum	NCS1	33.766	154	87	5	10	163	144	282	1.78E-19	84.3
Q4QTJ2.1	EVM0007329.1	Papaver somniferum	NCS1	33.766	154	87	5	10	163	144	282	2.25E-19	84.3
Q4QTJ2.1	EVM0012460.1	Papaver somniferum	NCS1	34.167	120	71	4	79	192	3	120	1.97E-15	70.1
Q6WUC1.1	EVM0008621.1	Papaver somniferum	6OMT	54.802	354	149	6	1	346	1	351	1.27E-140	402
Q6WUC1.1	EVM0011328.1	Papaver somniferum	6OMT	53.803	355	152	7	1	346	1	352	1.11E-134	387
Q6WUC1.1	EVM0008782.1	Papaver somniferum	6OMT	46.957	345	171	7	12	346	29	371	1.96E-121	353
Q6WUC1.1	EVM0014836.1	Papaver	6OMT	45.455	341	179	5	8	345	4	340	2.05E-113	332

		somniferum											
Q6WUC1.1	EVM0014567.1	Papaver somniferum	6OMT	46.11	347	172	10	8	345	15	355	1.44E-104	310
Q6WUC1.1	EVM0012543.1	Papaver somniferum	6OMT	43.228	347	168	10	8	346	15	340	6.75E-95	285
Q6WUC1.1	EVM0005978.1	Papaver somniferum	6OMT	41.36	353	187	8	8	345	3	350	2.07E-87	266
Q6WUC1.1	EVM0006633.1	Papaver somniferum	6OMT	42.9	331	173	11	8	328	15	339	5.47E-86	262
Q6WUC1.1	EVM0004237.1	Papaver somniferum	6OMT	40.793	353	189	9	8	345	3	350	3.75E-82	253
Q7XB08.1	EVM0002778.1	Papaver somniferum	CNMT	59.026	349	142	1	3	351	8	355	1.49E-157	445
Q7XB08.1	EVM0000927_1	Papaver somniferum	CNMT	59.026	349	143	0	3	351	8	356	9.20E-157	443
Q7XB08.1	EVM0006100.1	Papaver somniferum	CNMT	54.046	346	159	0	6	351	8	353	2.08E-138	396
Q7XB08.1	EVM0003935_1	Papaver somniferum	CNMT	53.179	346	162	0	6	351	14	359	4.92E-137	393
Q7XB08.1	EVM0017526.1	Papaver somniferum	CNMT	53.314	347	161	1	6	351	8	354	5.89E-134	385
Q7XB08.1	EVM0015233.1	Papaver somniferum	CNMT	52.45	347	164	1	6	351	8	354	4.69E-133	383
Q7XB08.1	EVM0014166.1	Papaver somniferum	CNMT	51.594	345	167	0	7	351	16	360	5.27E-130	375
Q7XB08.1	EVM0002645.1	Papaver	CNMT	52.312	346	165	0	6	351	8	353	3.70E-129	373

		somniferum											
Q7XB08.1	EVM0012281.1	Papaver somniferum	CNMT	51.312	343	164	2	9	351	17	356	4.89E-125	362
Q7XB08.1	EVM0005266.1	Papaver somniferum	CNMT	49.854	343	169	2	9	351	17	356	8.86E-119	347
Q7XB08.1	EVM0012664.1	Papaver somniferum	CNMT	48.462	260	100	6	92	351	35	260	5.62E-70	218
Q9SP06.1	EVM0002703.1	Papaver somniferum	NMCH	64.358	491	153	5	12	480	18	508	0	624
Q9SP06.1	EVM0012200.1	Papaver somniferum	NMCH	50	474	231	4	10	480	13	483	2.89E-178	508
Q9SP06.1	EVM0008901.1	Papaver somniferum	NMCH	46.567	466	240	6	19	481	24	483	2.26E-160	462
Q9SP06.1	EVM0001635.1	Papaver somniferum	NMCH	45.396	467	237	5	21	478	33	490	1.18E-149	435
Q9SP06.1	EVM0009341.1	Papaver somniferum	NMCH	40.161	498	279	7	1	481	6	501	1.37E-135	400
Q9SP06.1	EVM0016340.1	Papaver somniferum	NMCH	40.606	495	275	8	2	481	7	497	3.25E-134	396
Q9SP06.1	EVM0001459.1	Papaver somniferum	NMCH	40.161	498	277	8	1	481	6	499	1.37E-133	394
Q9SP06.1	EVM0011934.1	Papaver somniferum	NMCH	44.946	465	239	7	25	480	47	503	3.70E-133	394
Q9SP06.1	EVM0007937.1	Papaver somniferum	NMCH	40.461	477	272	6	12	478	24	498	3.92E-132	390
Q7XB10.1	EVM0008782.1	Papaver	4'OMT2	54.81	343	149	3	20	357	29	370	1.70E-143	410

		somniferum											
Q7XB10.1	EVM0008621.1	Papaver somniferum	4'OMT2	47.059	340	178	1	20	357	11	350	3.59E-121	353
Q7XB10.1	EVM0011328.1	Papaver somniferum	4'OMT2	47.076	342	176	3	20	357	11	351	1.34E-118	346
Q7XB10.1	EVM0014567.1	Papaver somniferum	4'OMT2	44.321	361	185	7	6	357	2	355	2.46E-108	320
Q7XB10.1	EVM0014836.1	Papaver somniferum	4'OMT2	43.824	340	182	5	20	357	8	340	4.31E-107	316
Q7XB10.1	EVM0012543.1	Papaver somniferum	4'OMT2	41.595	351	178	6	12	357	11	339	7.27E-95	286
Q7XB10.1	EVM0006633.1	Papaver somniferum	4'OMT2	41.908	346	178	8	6	338	2	337	3.10E-91	276
Q7XB10.1	EVM0005978.1	Papaver somniferum	4'OMT2	40.634	347	194	6	20	357	7	350	1.33E-90	275
Q7XB10.1	EVM0004237.1	Papaver somniferum	4'OMT2	40.346	347	195	6	20	357	7	350	3.73E-89	271
Q7XB10.1	EVM0016742.1	Papaver somniferum	4'OMT2	40.616	357	191	9	17	357	12	363	1.24E-74	234
AAC61839.1	EVM0011203.1	Papaver somniferum	BBE	46.729	535	270	8	3	526	4	534	6.05E-165	479
AAC61839.1	EVM0006678.1	Papaver somniferum	BBE	49.813	534	233	9	33	532	52	584	1.27E-164	479
AAC61839.1	EVM0000976.1	Papaver somniferum	BBE	48.444	514	241	10	31	523	28	538	3.94E-147	432
AAC61839.1	EVM0008977.1	Papaver	BBE	47.654	405	203	4	127	523	1	404	1.12E-123	368

		somniferum											
B5UAQ8.1	EVM0004494.1	Eschscholzia californica	CFS	45.902	488	243	8	18	487	24	508	9.74E-153	444
Q50LH4.1	EVM0004494.1	Eschscholzia californica	SPS	45	500	252	8	17	495	12	509	2.16E-151	441
Q108P1.1	EVM0000927_1	Papaver somniferum	TNMT	50.725	345	169	1	14	358	13	356	3.16E-129	373
Q108P1.1	EVM0002778.1	Papaver somniferum	TNMT	49.855	345	171	1	14	358	13	355	8.7E-129	372
Q108P1.1	EVM0003935_1	Papaver somniferum	TNMT	48.696	345	176	1	14	358	16	359	8.85E-124	360
Q108P1.1	EVM0006100.1	Papaver somniferum	TNMT	47.578	351	183	1	8	358	4	353	4.51E-122	355
Q108P1.1	EVM0015233.1	Papaver somniferum	TNMT	46.875	352	185	2	8	358	4	354	1.68E-119	348
Q108P1.1	EVM0017526.1	Papaver somniferum	TNMT	46.591	352	186	2	8	358	4	354	3.21E-118	345
Q108P1.1	EVM0002645.1	Papaver somniferum	TNMT	47.11	346	182	1	13	358	9	353	1.46E-114	336
Q108P1.1	EVM0014166.1	Papaver somniferum	TNMT	45.584	351	190	1	8	358	11	360	5.27E-114	335
Q108P1.1	EVM0012281.1	Papaver somniferum	TNMT	45.481	343	183	2	16	358	18	356	2.83E-106	315
Q108P1.1	EVM0005266.1	Papaver somniferum	TNMT	46.939	343	178	2	16	358	18	356	1.13E-103	308
Q108P1.1	EVM0012664.1	Papaver	TNMT	40.702	285	131	9	76	358	12	260	2.98E-56	183

		somniferum											
L7X0L7.1	EVM0007338.1	Papaver somniferum	P6H	42.495	553	281	10	7	537	3	540	1.37E-154	452
L7X0L7.1	EVM0006719.1	Papaver somniferum	P6H	45.267	486	254	6	55	532	40	521	1.43E-152	446
L7X0L7.1	EVM0009330.1	Papaver somniferum	P6H	42.202	545	286	10	7	538	4	532	1.56E-152	446
L7X0L7.1	EVM0005670.1	Papaver somniferum	P6H	45.062	486	255	6	55	532	40	521	5.52E-150	440
L7X0L7.1	EVM0002156.1	Papaver somniferum	P6H	45.528	492	257	8	54	537	37	525	1.97E-148	436
L7X0L7.1	EVM0000405.1	Papaver somniferum	P6H	43.487	522	280	9	22	537	35	547	2.07E-148	436
L7X0L7.1	EVM0018114.1	Papaver somniferum	P6H	44.19	525	271	12	18	537	7	514	1.38E-146	431
L7X0L7.1	EVM0015053.1	Papaver somniferum	P6H	44	500	266	9	22	515	11	502	8.3E-146	429
L7X0L7.1	EVM0009551.1	Papaver somniferum	P6H	42.505	527	283	11	20	537	16	531	1.29E-142	421
L7X0L7.1	EVM0017761.1	Papaver somniferum	P6H	45.695	453	235	6	55	501	4	451	1.61E-142	418
L7X0L7.1	EVM0004610.1	Papaver somniferum	P6H	42.095	525	280	10	20	537	16	523	2.47E-142	420
L7X0L7.1	EVM0007794.1	Papaver somniferum	P6H	41.935	527	279	10	21	538	13	521	1.12E-141	418
L7X0L7.1	EVM0009476.1	Papaver	P6H	40.805	522	292	7	20	537	7	515	1.73E-141	417

		somniferum											
L7X0L7.1	EVM0005523.1	Papaver somniferum	P6H	43.086	499	259	7	45	537	35	514	1.1E-140	416
L7X0L7.1	EVM0000133.1	Papaver somniferum	P6H	44.266	497	245	9	48	537	32	503	1.96E-140	414
L7X0L7.1	EVM0017491.1	Papaver somniferum	P6H	41.746	527	283	9	21	538	13	524	1.19E-139	413
L7X0L7.1	EVM0005930.1	Papaver somniferum	P6H	45.183	436	224	7	58	482	44	475	1.65E-139	411
L7X0L7.1	EVM0016799.1	Papaver somniferum	P6H	41.758	546	295	12	1	532	1	537	2.39E-139	413
L7X0L7.1	EVM0003486.1	Papaver somniferum	P6H	44.534	494	259	8	55	538	40	528	3.73E-139	412
L7X0L7.1	EVM0017004.1	Papaver somniferum	P6H	42.254	497	261	9	46	537	27	502	2.41E-138	409
L7X0L7.1	EVM0004589.1	Papaver somniferum	P6H	43.408	493	266	6	55	538	40	528	4.66E-137	407
L7X0L7.1	EVM0012792.1	Papaver somniferum	P6H	41.4	500	257	9	7	484	3	488	1.05E-134	399
L7X0L7.1	EVM0000340.1	Papaver somniferum	P6H	41.551	503	265	11	40	538	21	498	2.38E-128	383
L7X0L7.1	EVM0013378.1	Papaver somniferum	P6H	41.017	295	128	10	269	538	2	275	2.54E-62	207
L7X3S1.1	EVM0006719.1	Papaver somniferum	MSH	48.77	488	237	8	45	524	39	521	7.91E-165	477
L7X3S1.1	EVM0005670.1	Papaver	MSH	49.18	488	235	8	45	524	39	521	1.1E-163	474

		somniferum											
L7X3S1.1	EVM0009330.1	Papaver somniferum	MSH	46.226	530	264	10	12	524	1	526	2.39E-162	471
L7X3S1.1	EVM0003486.1	Papaver somniferum	MSH	48.697	499	240	9	36	524	30	522	9.31E-158	459
L7X3S1.1	EVM0002156.1	Papaver somniferum	MSH	47.106	501	247	11	34	524	28	520	4.22E-155	452
L7X3S1.1	EVM0018114.1	Papaver somniferum	MSH	47.269	531	240	16	12	524	1	509	6.93E-154	449
L7X3S1.1	EVM0004589.1	Papaver somniferum	MSH	47.495	499	246	8	36	524	30	522	2.29E-153	447
L7X3S1.1	EVM0007338.1	Papaver somniferum	MSH	44.867	526	260	11	22	524	17	535	6.41E-152	444
L7X3S1.1	EVM0000405.1	Papaver somniferum	MSH	44.618	511	268	7	24	524	37	542	1.82E-149	438
L7X3S1.1	EVM0009551.1	Papaver somniferum	MSH	45.233	493	252	8	39	524	45	526	2.2E-149	437
L7X3S1.1	EVM0015053.1	Papaver somniferum	MSH	45.935	492	254	8	34	521	30	513	4.66E-149	437
L7X3S1.1	EVM0009476.1	Papaver somniferum	MSH	44.401	509	268	10	22	524	11	510	8.1E-148	433
L7X3S1.1	EVM0004610.1	Papaver somniferum	MSH	46.694	484	241	10	44	524	49	518	2.06E-147	432
L7X3S1.1	EVM0017761.1	Papaver somniferum	MSH	46.799	453	233	5	45	493	3	451	2E-145	425
L7X3S1.1	EVM0007794.1	Papaver	MSH	43.13	524	270	11	14	524	7	515	2.51E-145	427

		somniferum											
L7X3S1.1	EVM0005523.1	Papaver somniferum	MSH	44.311	501	250	10	33	524	29	509	5.56E-145	426
L7X3S1.1	EVM0017004.1	Papaver somniferum	MSH	42.829	509	263	9	22	524	11	497	2.52E-141	416
L7X3S1.1	EVM0000340.1	Papaver somniferum	MSH	43.57	521	257	12	12	524	1	492	5.15E-138	407
L7X3S1.1	EVM0017491.1	Papaver somniferum	MSH	43.21	486	258	9	14	489	7	484	8.81E-138	408
L7X3S1.1	EVM0012792.1	Papaver somniferum	MSH	44.581	489	234	12	12	470	1	482	9.02E-137	404
L7X3S1.1	EVM0000133.1	Papaver somniferum	MSH	43.287	499	252	10	33	524	24	498	1.05E-136	404
L7X3S1.1	EVM0005930.1	Papaver somniferum	MSH	46.014	439	221	9	47	474	42	475	2.98E-136	402
L7X3S1.1	EVM0016799.1	Papaver somniferum	MSH	42.884	534	277	14	14	524	9	537	4.05E-132	394
L7X3S1.1	EVM0008748.1	Papaver somniferum	MSH	40.974	493	260	10	54	524	68	551	8.18E-127	381
L7X3S1.1	EVM0007461.1	Papaver somniferum	MSH	41.752	491	265	11	46	524	52	533	9.33E-118	357
AGL44334.1	EVM0016944.1	Papaver somniferum	DBOX	47.843	510	244	11	35	530	38	539	5.04E-158	461
AGL44334.1	EVM0006867.1	Papaver somniferum	DBOX	44.776	536	285	7	3	531	1	532	1.95E-155	454
AGL44334.1	EVM0010203.1	Papaver	DBOX	44.61	538	288	7	1	531	1	535	4.81E-155	453

		somniferum											
AGL44334.1	EVM0016772.1	Papaver somniferum	DBOX	46.337	505	259	6	35	531	39	539	3.62E-154	451
AGL44334.1	EVM0013897.1	Papaver somniferum	DBOX	44.424	538	286	9	3	531	1	534	1.71E-149	439
AGL44334.1	EVM0004268.1	Papaver somniferum	DBOX	47.755	490	235	9	51	531	37	514	3.53E-146	431
AGL44334.1	EVM0004314.1	Papaver somniferum	DBOX	44.052	538	280	9	2	531	34	558	3.67E-146	431
AGL44334.1	EVM0009143.1	Papaver somniferum	DBOX	44.353	487	217	10	51	531	55	493	1.35E-122	370
AGL44334.1	EVM0008977.1	Papaver somniferum	DBOX	40.196	408	233	6	133	532	1	405	1.9E-92	288
A0A1C9U5X5.1	EVM0000927_1	Papaver somniferum	RNMT	50.852	352	167	4	10	358	8	356	2.74E-133	384
A0A1C9U5X5.1	EVM0003935_2	Papaver somniferum	RNMT	51.944	360	167	4	2	358	4	360	1.39E-132	382
A0A1C9U5X5.1	EVM0002778.1	Papaver somniferum	RNMT	49.716	352	170	4	10	358	8	355	1.37E-130	377
A0A1C9U5X5.1	EVM0014166.1	Papaver somniferum	RNMT	50.833	360	171	4	2	358	4	360	1.06E-125	365
A0A1C9U5X5.1	EVM0006100.1	Papaver somniferum	RNMT	51.009	347	164	4	15	358	10	353	5.32E-125	363
A0A1C9U5X5.1	EVM0015233.1	Papaver somniferum	RNMT	51.149	348	163	5	15	358	10	354	2.98E-124	361
A0A1C9U5X5.1	EVM0017526.1	Papaver	RNMT	50.287	348	166	5	15	358	10	354	2.30E-121	353

		somniferum											
A0A1C9U5X5.1	EVM0002645.1	Papaver somniferum	RNMT	46.991	349	179	4	13	358	8	353	1.19E-112	332
A0A1C9U5X5.1	EVM0012281.1	Papaver somniferum	RNMT	43.109	341	187	4	20	358	21	356	1.83E-103	308
A0A1C9U5X5.1	EVM0005266.1	Papaver somniferum	RNMT	44.152	342	182	5	20	358	21	356	1.77E-101	303
A0A1C9U5X5.1	EVM0012664.1	Papaver somniferum	RNMT	40.293	273	124	7	90	358	23	260	2.04E-57	187
I3V6A7	EVM0000250.1	Papaver somniferum	SOMT	40.62	352	170	9	14	329	42	390	1.00E-76	233
I3V6A7	EVM0002781.1	Papaver somniferum	SOMT	42.08	366	188	7	2	346	28	390	5.00E-97	286
3V6A7	EVM0013337.1	Papaver somniferum	SOMT	43.44	366	195	6	9	362	25	390	7.00E-101	297
Q948Y1	EVM0004494.1	Coptis japonica	CAS	50.63	478	216	8	35	510	32	491	2.00E-163	466
BAJ40864.1	EVM0009143.1	Coptis japonica	STOX	43.84	536	238	8	14	495	8	534	2.00E-133	392
BAJ40864.1	EVM0016772.1	Coptis japonica	STOX	47.37	513	255	8	33	539	29	532	3.00E-161	464
BAJ40864.1	EVM0016944.1	Coptis japonica	STOX	47.44	546	264	9	1	539	2	531	8.00E-168	481
BAJ40864.1	EVM0010203.1	Coptis japonica	STOX	47.64	508	253	8	33	535	33	532	4.00E-146	425
BAJ40864.1	EVM0004314.1	Coptis	STOX	48.43	508	251	7	58	560	33	534	5.00E-155	449

		japonica											
BAJ40864.1	EVM0013897.1	Coptis japonica	STOX	48.62	508	250	7	34	536	33	534	2.00E-157	454
BAJ40864.1	EVM0006867.1	Coptis japonica	STOX	48.62	506	248	7	33	532	33	532	1.00E-155	449
BAJ40864.1	EVM0004268.1	Coptis japonica	STOX	48.66	524	249	7	3	516	21	534	2.00E-161	465

Table S11. GenBank accession numbers of complete chloroplast genomes of 36 species used for phylogenetic analysis.

**Species**	**GenBank acc.**
Ginkgo biloba	MN443423.1
Amborella trichopoda	NC_005086
Nymphaea colorata	NC_057562.1
Chloranthus spicatus	NC_009598
Aristolochia contorta	NC_036152.1
Aristolochia fimbriata	CM034085.1
Piper nigrum	NC_034692
Piper kadsura	NC_027941
Litsea cubeba	NC_048954.1
Phoebe sheareri	KX437773.1
Cinnamomum micranthum	NC_035802.1
Magnolia grandiflora	NC_020318.1
Liriodendron chinense	NC_030504.1
Lemna minor	NC_010109.1
Oryza sativa	NC_031333.1
Lilium brownii	NC_035588.1
Dendrobium officinale	NC_024019.1
Ceratophyllum demersum	NC_009962.1
Papaver somniferum	NC_029434.1
Macleaya cordata	MT178411.1
Trollius chinensis	NC_031849
Nelumbo nucifera	JQ336993.1
Buxus microphylla	NC_009599.1
Vitis vinifera	NC_007957.1

Glycine soja	NC_022868
Fragaria vesca	NC_015206.1
Citrus reticulata	NC_034671.1
Arabidopsis thaliana	NC_000932
Theobroma cacao	NC_014676.2
Cornus chinensis	NC_044815.1
Coffea canephora	NC_030053.1
Mentha canadensis	MN102358.1
Nicotiana tabacum	NC_001879
Helianthus annuus	NC_007977.1
Daucus carota	NC_008325.1
Lonicera japonica	NC_026839.1

Table S12. Primers used for cloning AcOMTs

Gene	Primer
AcOMT1 -F	GGATCCATGGAAACACCGAAAGGCG
AcOMT1-R	GCGGCCGCTTAGTAAGGAAATGCCTCAATCACAGA
AcOMT2 -F	GGATCCATGGAGACACCGAAAGACGATCA
AcOMT2-R	GCGGCCGCTTAGTAAGGAAATGCCTCAATCACAGA
AcOMT3 -F	GGATCCATGAAGATGGATATCATGAACCTGC
AcOMT3-R	GCGGCCGCATAGGGGAAGGCCTCGATCACAGATTG
AcOMT5 -F	GGATCCATGGCCGATCCAGTTGCAG
AcOMT5 -R	GCGGCCGCAGGATAAGCTTCAATTACAG
AcOMT7-F	GAGCTCATGGAGGCGGAAAAGGACGTTC
AcOMT7-R	GCGGCCGCAGGGAAGAGTTCGATAATTGATTCG

Table S13. Primer sequences used for qPCR

Gene ID	Gene Description	Forward Primer sequences (5'-3′)	Reverse Primer sequences (5'-3′)
EVM0012696	Actin	TTCAATGTCCCAGCCATGTACGTT	ACCGGAATCCAGTACAATACCAG
EVM0008901	CYP80G	CCTAGATAGCGCTGAAGTC	CGGAGAAGAAGGGTTTGAGC
EVM0002703	CYP80B	CCTGCTTATGGAAATGTTCGG	GTCTCTGAGCCTTGACGTG
EVM0012200	CYP80	CAGCTACCTTCACCAATCC	CTTGGGAGAAGATGGGG
EVM0001635	CYP80	CAGTGGAGTTTAGCATACGG	CGTCTCTTGGTCGATAAAGTC
EVM0008024	CYP81	CAACCCATTGGAGGCAG	GCAGTACTGAAGCTCGCTC
EVM0004494	CYP719	GGAGAATATTGGTAAACCAC	CCAAATGGAAGGAAGGATC
EVM0011328	OMT	GGAAGCTGTGCCCATAAAAGG	GGGTTTGGGGACGATCA
EVM0008782	OMT	GCAGATGAGGAAAGGAGAGG	GCCGAAGATGTGTTTCCAAAG
EVM0014836	OMT	GCTAAAGACCCCAAAGCTAACCAG	CATTAAGGATCCCGTCGCTCT
EVM0014567	OMT	TATGGAAACTCACCCACGCCTTC	CGCCGTGGGAATGGATAATA
EVM0016813	OMT	GCCATGCCCATTCTCGAT	TCCTTGTCCGCGTCCAC
EVM0011362	OMT	GCTCTTCCATACCTCGACCAC	TAGTTCGTCTTGTCAGCGTCT
EVM0013337	OMT	AGGCACCAATTTCGATCTGCAA	CTCAGCACTTGGAACTTTGACA
EVM0006633	OMT	GATCAGTTGCACCGTCTTCG	GTCTCCTTTAACTCGAGCGAT
EVM0002543	OMT	CTTGGTGCTGGTTATTCCGGGTA	CGCCATCCACATTGAAGGGT
